# Pd-Catalyzed asymmetric allylic amination with isatin using a P,olefin-type chiral ligand with C–N bond axial chirality

**DOI:** 10.3762/bjoc.21.83

**Published:** 2025-05-23

**Authors:** Natsume Akimoto, Kaho Takaya, Yoshio Kasashima, Kohei Watanabe, Yasushi Yoshida, Takashi Mino

**Affiliations:** 1 Graduate School of Engineering, Chiba University, 1-33 Yayoi-cho, Inage-ku, Chiba 263-8522, Japan; 2 Education Center, Chiba Institute of Technology, 2-2-1 Shibazono, Narashino, Chiba 275-0023, Japan; 3 Faculty of Education, Chiba University, 1-33 Yayoi-cho, Inage-ku, Chiba 263-8522, Japan; 4 Molecular Chirality Research Center, Chiba University, 1-33 Yayoi-cho, Inage-ku, Chiba 263-8522, Japan; 5 Soft Molecular Activation Research Center, Chiba University, 1-33 Yayoi-cho, Inage-ku, Chiba 263-8522, Japan; 6 Institute for Advanced Academic Research (IAAR), Chiba University, 1-33 Yayoi-cho, Inage-ku, Chiba 263-8522, Japan

**Keywords:** asymmetric allylic amination, axial chirality, isatin, palladium catalysis, P,olefin-type chiral ligand

## Abstract

In this study, we implemented the P,olefin-type chiral ligand (a*R*)-(−)-**6**, which contains a cyclohexyl group and a cinnamoyl group on the nitrogen atom, in the Pd-catalyzed asymmetric allylic amination of allylic esters with isatin derivatives **11** as nucleophiles. The reaction proceeds efficiently, yielding the products (*S*)-**13** with good-to-high enantioselectivity. A scale-up reaction was also successfully conducted at a 1 mmol scale. Additionally, when malononitrile was added to the resulting product (*S*)-**13a** in the presence of FeCl_3_ as the catalyst, the corresponding malononitrile derivative (*S*)-**16** was obtained without any loss in optical purity.

## Introduction

**Figure 1:**
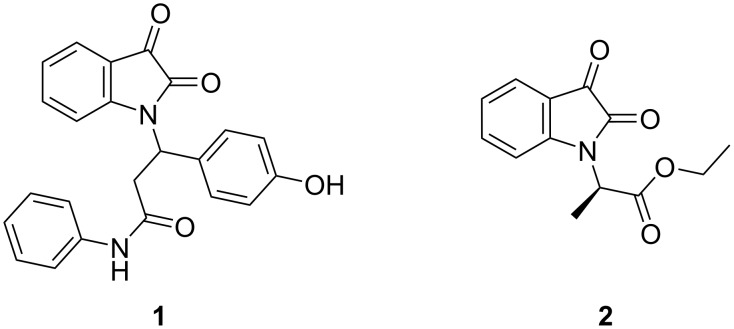
Compound **1** and **2**.

Isatin is a well-known natural indole derivative. Due to the broad biological activities of its derivatives, extensive research has been conducted on their synthesis. Furthermore, the isatin framework is a versatile starting material for various transformations, including multicomponent reactions and the synthesis of spirocyclic compounds [1–3]. The nucleophilicity of isatin at the nitrogen atom allows it to participate in reactions such as alkylation [4], arylation [5], and *aza*-Michael addition [6–8]. However, the products obtained from these reactions are primarily achiral or racemic, and only a few studies have reported the use of isatin as a nucleophile in asymmetric reactions [9–11]. On the other hand, it has been revealed that compounds in which the carbon bonded to the nitrogen atom of newly constructed *N*-substituted isatin becomes a chiral center exhibit pharmacological properties in medicinal chemistry. For example, racemic compound **1** (Figure 1) was evaluated for its cytotoxicity against human breast cancer cells (MCF7) in comparison to the standard doxorubicin and exhibited excellent activity against the MCF7 cell line [12]. The optically active compound **2** also showed activity against Huh7.5-FGR-JC1-Rluc2A cells, which carry HCV gt 2a [13].

**Figure 2:**
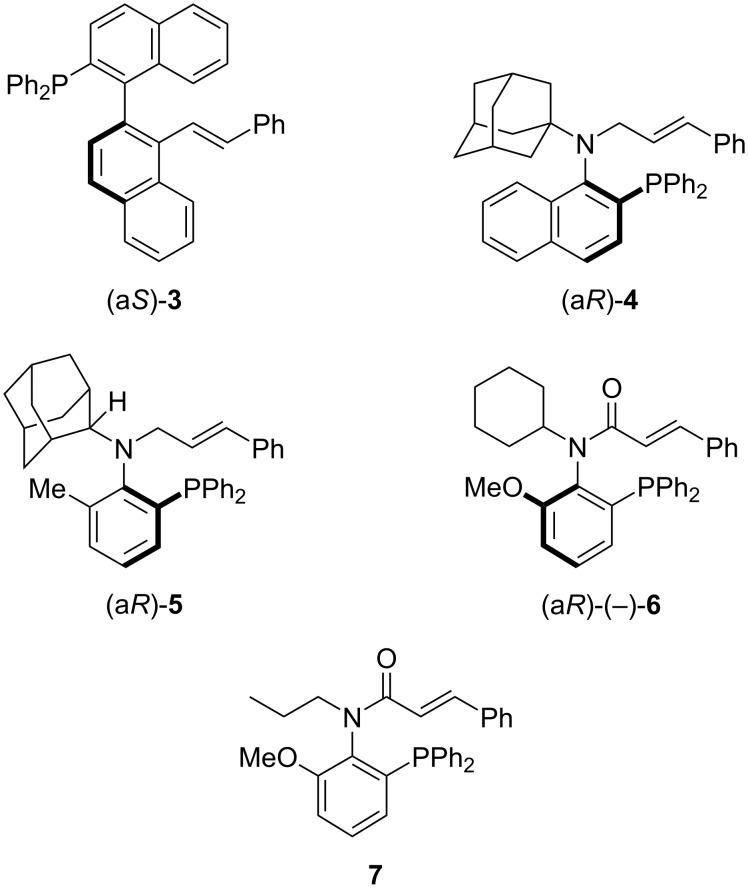
Chiral ligands **3**–**7**.

Therefore, developing asymmetric reactions that simultaneously form a carbon–nitrogen bond and construct a chiral center is of great importance. Although a relatively large number of asymmetric allylic amination reactions using palladium catalysts with amines as nucleophiles have been reported [14–25], there have been only a few reports on the *N*-substitution of isatin using asymmetric methods. Recently, Wolf’s group reported a transition-metal-catalyzed (Pd-catalyzed) asymmetric allylic amination of allyl esters using isatin as a nucleophile. In this reaction, bisphosphine-type ligands such as BINAP and SEGPHOS derivatives, as well as P,N-type ligands like oxazoline-type ligands, were utilized as chiral ligands [26]. On the other hand, several groups have recently reported new chiral ligands with axial chirality for Pd-catalyzed asymmetric allylic substitution reactions. For example, the Zhou group reported a P,olefin-type chiral ligand **3** with C–C bond axial chirality for this reaction (Figure 2) [27]. Additionally, we have recently reported chiral ligands with C–N bond axial chirality, such as *N*-alkyl-*N*-cinnamyl-type chiral ligands **4** [28–29] and **5** [30], and a P,olefin-type chiral ligand **6** [31] with a cinnamoyl group instead of a cinnamyl group. In particular, the chiral ligand **6** is effective in the Pd-catalyzed asymmetric allylic substitution reaction of allylic esters with indoles. Here, we describe the Pd-catalyzed asymmetric allylic amination of allylic esters with isatin as a nucleophile using chiral ligand **6** and its derivative **7**. Compared to chiral ligand **6**, which has a secondary alkyl group (cyclohexyl) as a substituent on the nitrogen and has already been reported, compound **7** has a primary alkyl group (*n*-propyl). This difference reduces steric hindrance and lowers the rotational barrier around the carbon–nitrogen bond, increasing the likelihood of racemization.

## Results and Discussion

**Scheme 1:**
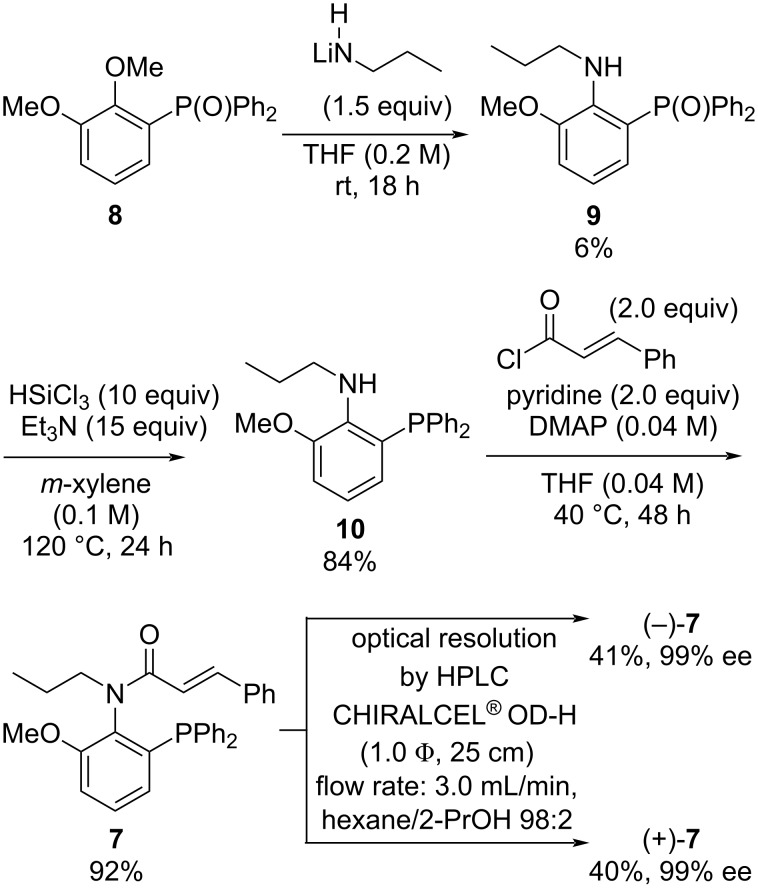
Preparation and optical resolution of **7**.

*N*-Propyl-*N*-cinnamoylamide **7** was prepared from phosphine oxide **8** [32] via an S_N_Ar reaction with nucleophilic lithium amide from *n*-propylamine, the reduction of phosphine oxide **9** by trichlorosilane/triethylamine, and the *N*-acylation of **10** with cinnamoyl chloride in three steps (Scheme 1). We also analyzed amide compound **7** by HPLC analysis using a chiral stationary phase column with a CD detector and found that the C(aryl)–N(amide) bond axial chirality exists in amide compound **7**. We attempted the optical resolution of racemic compound (±)-**7** and obtained (+)-**7** and (−)-**7** using a semi-preparative chiral HPLC on 50 milligram scales. We also investigated the racemization process associated with the axial chirality of compound **7** (see Supporting Information File 1). The racemization barrier (Δ*G*^‡^_rac_) of (−)-**7** in *n*-dodecane was determined to be 25.0 kcal/mol at 25 °C, as calculated using the Arrhenius and Eyring equations [33–35]. Therefore, the half-life of racemization of ligand (−)-**7** at 25 °C in *n*-dodecane is approximately 1.3 days, which is faster compared to ligand **6**, which has a half-life of about 3.7 days [31].

We next investigated the ability of optically active amides (a*R*)-(−)-**6** and (−)-**7** as chiral ligands for the Pd-catalyzed asymmetric allylic amination of allylic acetate, such as a 1,3-diphenyl-2-propenyl acetate (**12**) with isatin (**11a**). We began the investigation under conditions using 5 mol % of [Pd(C_3_H_5_)Cl]_2_ (Pd = 10 mol %) and 12 mol % of chiral ligands (Table 1).

**Table 1:** Optimization of conditions for the Pd-catalyzed asymmetric allylic amination of acetate **12** with isatin (**11a**).^a^

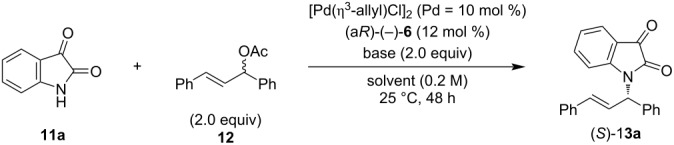				

Entry	Base	Solvent	Yield (%)^b^	ee (%)^c^

1	K_2_CO_3_	CHCl_3_	72	87
2^d^	K_2_CO_3_	CHCl_3_	3	84
3	Na_2_CO_3_	CHCl_3_	99	85
4	Cs_2_CO_3_	CHCl_3_	19	86
5	NaOAc	CHCl_3_	89	86
6	K_3_PO_4_	CHCl_3_	12	86
7	Na_3_PO_4_	CHCl_3_	60	88
8	Na_3_PO_4_	CH_2_Cl_2_	88	92
9	Na_3_PO_4_	CH_3_CN	75	93
10	Na_3_PO_4_	THF	74	93
11	Na_3_PO_4_	DMF	trace	–
12	Na_3_PO_4_	PhCF_3_	84	95
13^e^	Na_3_PO_4_	PhCF_3_	50	86
14^f^	Na_3_PO_4_	PhCF_3_	80	94

^a^The reaction was carried out at 0.1 mmol scale. ^b^Isolated yield. ^c^Determined by chiral HPLC analysis using a chiral column. Absolute configuration was assigned by comparison of HPLC analysis with reported data [26]. ^d^This reaction was carried out using (−)-**7** instead of (a*R*)-(−)-**6** as a chiral ligand. ^e^This reaction was carried out using 1,3-diphenylallyl pivalate (**14**) instead of acetate **12**. ^f^This reaction was carried out at a 1.0 mmol scale.

The reaction with (a*R*)-(−)-**6** as the chiral ligand and K_2_CO_3_ as the base in CHCl_3_ gave the desired product (*S*)-**13a** in 72% yield with 87% ee (Table 1, entry 1). In contrast, the reaction with (−)-**7** afforded (*S*)-**13a** in significantly lower yield, albeit with an enantioselectivity similar to that of the reaction with **6** (Table 1, entry 2). This result clarifies that (−)-**7**, with a racemization half-life of only approximately 1.3 days, also has a chiral induction ability. However, improvement is required in terms of the reactivity of the catalytic reaction. Subsequently, we investigated the effect of the base using (a*R*)-(−)-**6** by testing various bases. The reaction in the presence of Na_2_CO_3_ delivered the product in 99% yield, although the enantioselectivity slightly decreased compared to the reaction using K_2_CO_3_ (see Table 1, entry 1 vs entry 3). The use of Cs_2_CO_3_ resulted in a significant drop in the yield (Table 1, entry 4), whereas NaOAc improved the yield but slightly lowered the enantioselectivity (Table 1, entry 5). Other potassium salts such as K_3_PO_4_ led to a low yield of the product (Table 1, entry 6). Meanwhile, when Na_3_PO_4_ was tested, the yield decreased, but the enantioselectivity improved to 88% ee (Table 1, entry 7). With Na_3_PO_4_ as the optimum base, which showed the highest enantioselectivity, we conducted a solvent screening. The reaction in CH_2_Cl_2_ resulted in better yield and enantioselectivity than in CHCl_3_ (Table 1, entry 8). The coordinating solvents, CH_3_CN and THF, further improved the enantioselectivity to 93% ee (Table 1, entries 9 and 10). In contrast, the reaction barely proceeded when DMF was used (Table 1, entry 11). The reaction in PhCF_3_ afforded the target product in a good yield with the highest enantioselectivity compared to other solvents (Table 1, entry 12). Furthermore, when (*E*)-1,3-diphenyl-2-propenyl pivalate (**14**) was tested as the allyl ester, the desired product (*S*)-**13a** was obtained with a yield of 50% and an enantioselectivity of 86% ee (Table 1, entry 13). Additionally, the scale-up reaction using 1 mmol of isatin (**11a**) as the nucleophile under the optimal conditions (Table 1, entry 12) afforded the desired product (*S*)-**13a** with nearly the same yield and enantioselectivity as the 0.1 mmol scale reaction (entry 14).

**Scheme 2:**
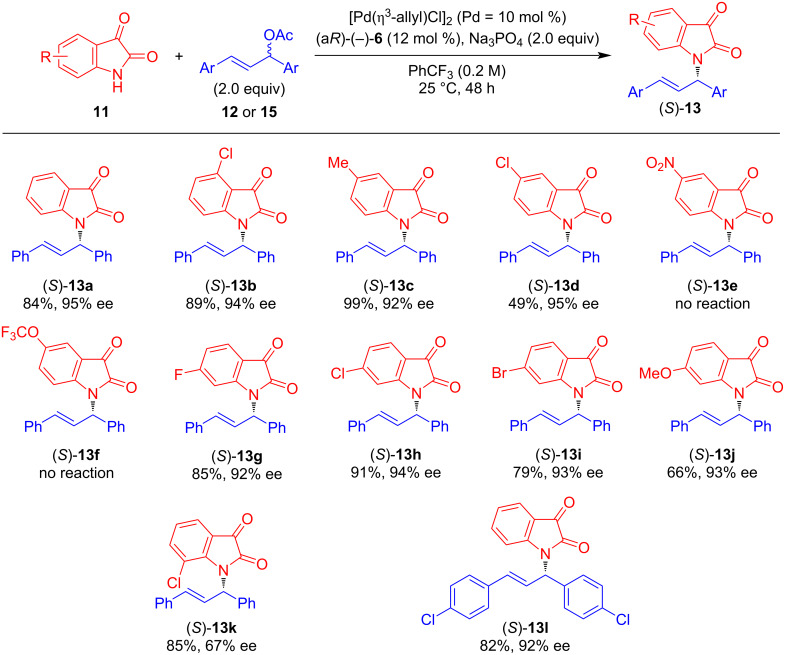
Pd-catalyzed asymmetric allylic amination of acetate **12** (Ar = Ph) or **15** (Ar = *p*-ClC_6_H_4_) with isatin derivatives **11** using (a*R*)-(−)-**6** as a chiral ligand: The reaction was carried out at 0.1 mmol scale; yields refer to isolated yields.

**Scheme 3:**
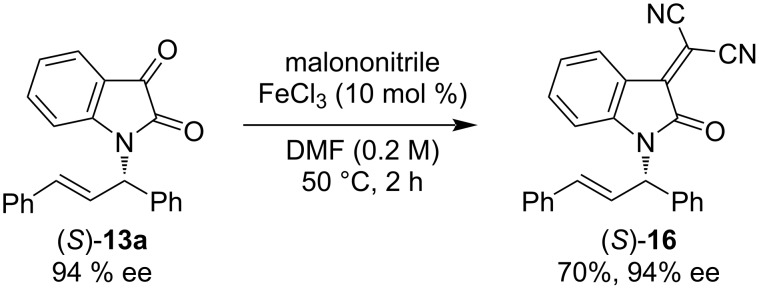
Transformation of the reaction product (*S*)-**13a**: The reaction was carried out at 0.1 mmol scale and the yield refers to the isolated yield.

Next, we investigated the substrate scope of the palladium-catalyzed asymmetric allylic amination of 1,3-diphenyl-2-propenyl acetate (**12**) with isatin derivatives **11** as nucleophiles under the optimized conditions using (a*R*)-(−)-**6** as the ligand and Na_3_PO_4_ as the base in PhCF_3_ as the solvent (Scheme 2). An isatin derivative bearing a chloro group at the 4-position afforded the desired product (*S*)-**13b** with good yield and enantioselectivity. Similarly, an isatin derivative with a methyl group as an electron-donating group at the 5-position gave (*S*)-**13c** in good yield, although with slightly decreased enantioselectivity. The introduction of the chloro group at the same position led to a moderate yield for (*S*)-**13d**, while the enantioselectivity remained high. In contrast, the reaction with the isatin derivative bearing a nitro group at the 5-position did not proceed, and (*S*)-**13e** was not produced. Likewise, no reaction occurred with a trifluoromethoxy-substituted derivative, resulting in no formation of (*S*)-**13f**. Reactions using isatin derivatives bearing halogen substituents at the 6-position proceeded efficiently, affording (*S*)-**13g**–**i** in good yields with high enantioselectivities. Conversely, the isatin derivative bearing a methoxy group at the 6-position led to a decreased yield for (*S*)-**13j**, though the enantioselectivity remained high. Additionally, we tested the reaction using an isatin derivative with a chloro group at the 7-position and obtained (*S*)-**13k** in good yield with moderate enantioselectivity. Furthermore, when (*E*)-1,3-di(*p*-chlorophenyl)-2-propenyl acetate (**15**) was utilized as an allylic acetate, the desired product (*S*)-**13l** was obtained in high yield with excellent enantioselectivity. We confirmed that the product **13** from the Pd-catalyzed asymmetric allylic amination of allyl esters with isatin using (a*R*)-(−)-**6** possesses an *S*-configuration. This stereochemical outcome follows the same reaction mechanism as the Pd-catalyzed asymmetric allylic substitution of allyl esters with indoles using (a*R*)-(−)-**6** [31]. To explore further applications of this product, we treated (*S*)-**13a** (94% ee) with malononitrile in the presence of FeCl_3_ as a catalyst [36] and obtained the corresponding malononitrile derivative (*S*)-**16** without any loss of optical purity (Scheme 3).

## Conclusion

In this study, *N*-propyl-*N*-cinnamoylamide **7** was synthesized in three steps from phosphine oxide **8**. Chiral HPLC analysis confirmed its axial chirality at the C(aryl)–N(amide) bond. The optical resolution of (±)-**7** yielded (+)-**7** and (−)-**7**. The racemization barrier of (−)-**7** in *n*-dodecane was determined to be 25.0 kcal/mol at 25 °C, with a half-life of approximately 1.3 days. The chiral amides (a*R*)-(−)-**6** and (−)-**7** were evaluated as ligands in Pd-catalyzed asymmetric allylic amination, and while (−)-**7** exhibited promising enantioselectivity, its yield was lower than (a*R*)-(−)-**6**. Further optimization of reaction conditions led to improved yields and enantioselectivities up to 95% ee. Moreover, the reaction was successfully scaled up to 1 mmol. The substrate scope was investigated using various isatin derivatives, yielding high enantioselectivities (up to 95% ee) for most, except for those bearing certain electron-withdrawing groups. Additionally, we demonstrated the further conversion of (*S*)-**13a** into the malononitrile derivative (*S*)-**16** without loss of optical purity.

## Supporting Information

Data of thermal racemization of **7**, DFT calculations for investigating racemization mechanism of **7**, general methods and materials, experimental procedures and characterization data, ^1^H, ^13^C and ^31^P NMR spectra for **9** and **10**, ^1^H, ^13^C and ^31^P NMR spectra and HPLC charts for (±)-**7**, (+)-**7** and (–)-**7**, ^1^H and ^13^C NMR spectra and HPLC charts for (*S*)-**13a**–**k** (except (*S*)-**13e**) and (*S*)-**16**.

File 1 Experimental section and compounds characterization.

## Data Availability

All data that supports the findings of this study is available in the published article and/or the supporting information of this article.
