# Supplementary material for: Pd-Catalyzed asymmetric allylic amination with isatin using a P,olefin-type chiral ligand with C–N bond axial chirality
Source: Beilstein J Org Chem. 2025 May 23;21:1018–23. doi: 10.3762/bjoc.21.83 (PMC12117205; doi:10.3762/bjoc.21.83)
Supplement: File 1 — Experimental section and compounds characterization. [file Beilstein_J_Org_Chem-21-1018-s001.pdf]

BEILSTEIN JOURNAL OF ORGANIC CHEMISTRY

Supporting Information

for

Pd-Catalyzed asymmetric allylic amination with isatin using a P,olefin-type chiral ligand with C–N bond axial chirality

Natsume Akimoto, Kaho Takaya, Yoshio Kasashima, Kohei Watanabe, Yasushi Yoshida and Takashi Mino

Beilstein J. Org. Chem. **2025,** *21,* 1018–1023. doi:10.3762/bjoc.21.83

Experimental section and compounds characterization

License and Terms: This is a supporting information file under the terms of the Creative Commons Attribution License (https://creativecommons.org/licenses/by/4.0). Please note that the reuse, redistribution and reproduction in particular requires that the author(s) and source are credited and that individual graphics may be subject to special legal provisions.
The license is subject to the *Beilstein Journal of Organic Chemistry* terms and conditions: (https://www.beilstein-journals.org/bjoc/terms)

Table of contents

Data of thermal racemization of (−)-**7** (Table S1, Figure S1, Table S2) S2

DFT calculations for investigating structural changes in the racemization of **7** S3

General information for DFT calculations S4

General methods and materials S5

Experimental procedures and characterization data S5

Copies of ^{1}H, ^{13}C and ^{31}P NMR spectra of **9** and **10** S12

Copies of ^{1}H, ^{13}C and ^{31}P NMR spectra and HPLC charts of (±)-**7**, (−)-**7** and (+)-**7** S15

Copies of ^{1}H and ^{13}C NMR and HPLC charts of (*S*)-**13** and (*S*)-**16** S30

Cartesian coordinates and energies S69

Table S1. Time dependence of ee value of (–)-**7** at various temperatures.[a]

Time (h)	Ee (%) at 40 °C	Ee (%) at 45 °C	Ee (%) at 50 °C	Ee (%) at 60 °C
0	94.0	95.4	95.8	93.2
0.5	90.1	84.0	79.0	47.8
1	84.0	75.6	64.2	23.8
2	72.2	53.2	41.8	6.0
4	54.6	34.2	18.8	-
8	29.2	-	-	-

[a] ee was determined by chiral HPLC analysis.

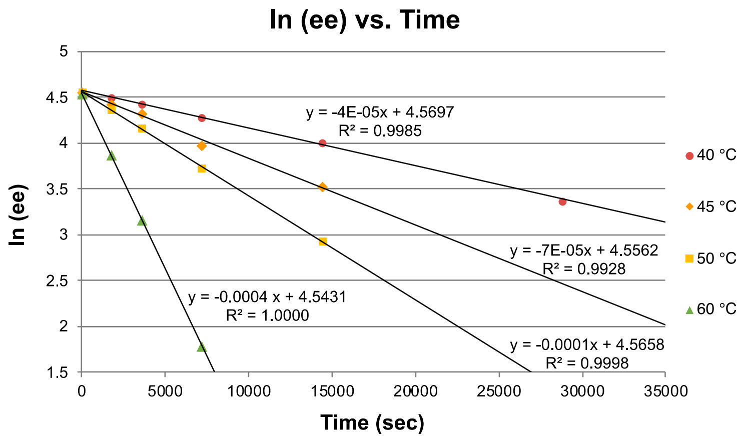

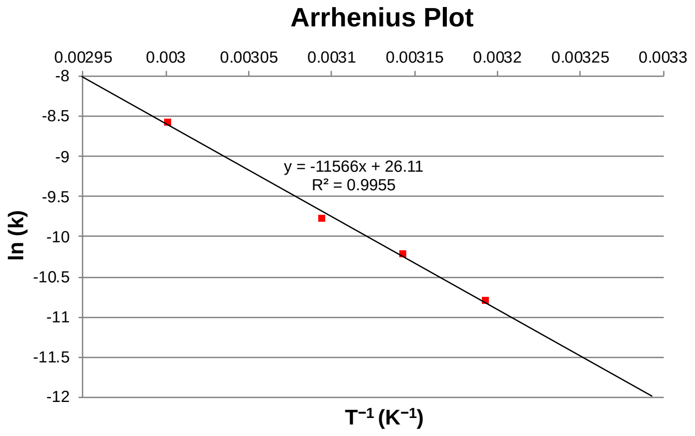

Figure S1. Data for the racemization and Arrhenius plot of (–)-**7**.

Table S2. Racemization parameters of (–)-**7** in *n*-dodecane at 25 °C.

E_{rac} kcal/mol	$\Delta H^{\ddagger}_{rac}$ kcal/mol	$\Delta S^{\ddagger}_{rac}$ cal/(mol·K)	$\Delta G^{\ddagger}_{rac}$ kcal/mol	k_{rac} 10^{-6} s^{-1}	$t_{1/2}$ days
23.0	22.4	−8.64	25.0	3.11	1.29

DFT Calculations for investigating structural changes in the racemization of 7

We investigated the structural changes and the associated energy of C–N bond rotation during the racemization of compound **7** using DFT calculations. To analyze this process, we systematically varied the dihedral angle between the carbon bonded to the methoxy group and the carbonyl carbon, optimizing each structure accordingly. The relationship between Gibbs free energy and the dihedral angle is shown in Figure S1. This indicated that the energy barrier for C–N bond rotation is +22.4 kcal/mol at a dihedral angle of 8° (◯,1), where the cinnamoyl group is positioned on the methoxy group side. This rotation then yields (*S*)-**7** with the rotated cinnamoyl group (◯,2), which was slightly less stable than (*R*)-**7** (◯,0) without cinnamoyl rotation (Δ*G* = +4.3 kcal/mol). The energy barrier for C–N bond rotation from this rotated isomer is +26.0 kcal/mol at a dihedral angle of 174° (◯,3), where the cinnamoyl group is positioned on the phosphine side. These results suggest that C–N bond rotation preferentially proceeds via a pathway in which the cinnamoyl group crosses the methoxy group rather than the phosphine group, although the calculated energy barrier slightly differs from the experimental result due to the level of basis set used in these calculations.

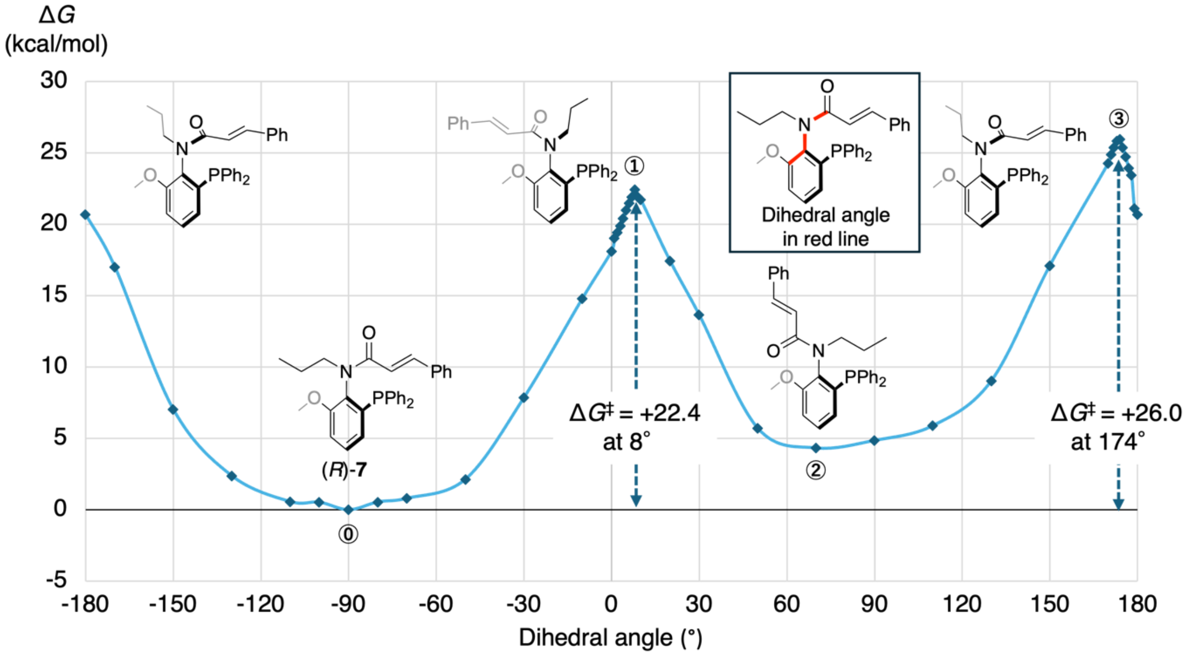

Figure S1. Plotted relation between dihedral angle and associated energy. Energy changes at the *ω*B97X-D/6-31+G* level are shown in kcal/mol.

General information for DFT calculations

All calculations were carried with the Gaussian 16 (revision C.01) program package.[1] The molecular structures and harmonic vibrational frequencies were obtained using ωB97X-D functional[2] and 6-31+G*[3] basis sets (for other atoms) were used. Geometry optimization and vibrational analysis were performed at the same level.

References

1. Gaussian 16, Revision C.01, Frisch, M. J.; Trucks, G. W.; Schlegel, H. B.; Scuseria, G. E.; Robb, M. A.; Cheeseman, J. R.; Scalmani, G.; Barone, V.; Petersson, G. A.; Nakatsuji, H.; Li, X.; Caricato, M.; Marenich, A. V.; Bloino, J.; Janesko, B. G.; Gomperts, R.; Mennucci, B.; Hratchian, H. P.; Ortiz, J. V.; Izmaylov, A. F.; Sonnenberg, J. L.; Williams-Young, D.; Ding, F.; Lipparini, F.; Egidi, F.; Goings, J.; Peng, B.; Petrone, A.; Henderson, T.; Ranasinghe, D.; Zakrzewski, V. G.; Gao, J.; Rega, N.; Zheng, G.; Liang, W.; Hada, M.; Ehara, M.; Toyota, K.; Fukuda, R.; Hasegawa, J.; Ishida, M.; Nakajima, T.; Honda, Y.; Kitao, O.; Nakai, H.; Vreven, T.; Throssell, K.; Montgomery, J. A., Jr.; Peralta, J. E.; Ogliaro, F.; Bearpark, M. J.; Heyd, J. J.; Brothers, E. N.; Kudin, K. N.; Staroverov, V. N.; Keith, T. A.; Kobayashi, R.; Normand, J.; Raghavachari, K.; Rendell, A. P.; Burant, J. C.; Iyengar, S. S.; Tomasi, J.; Cossi, M.; Millam, J. M.; Klene, M.; Adamo, C.; Cammi, R.; Ochterski, J. W.; Martin, R. L.; Morokuma, K.; Farkas, O.; Foresman, J. B.; Fox, D. J. Gaussian, Inc., Wallingford CT, 2016
2. Chai, J. D.; Head-gordon, M. Long-range corrected double-hybrid density functionals. *J. Chem. Phys.* **2009**, *131*, 174105–174117.
3. (5) (a) Clark, T.; Chandrasekhar, J.; Spitznagel, G. W.; Schleyer, P. V. R. Efficient Diffuse Function-Augmented Basis Sets for Anion Calculations. III. The 3-21+G Basis Set for First-Row Elements, Li-F. *J. Comput. Chem*. **1983**, *4*, 294–301. (b) Krishnan, R.; Binkley, J. S.; Seeger, R.; Pople, J. A. Self-consistent Molecular Orbital Methods. XX. A Basis Set for Correlated Wave Functions. *J. Chem. Phys*. **1980**, *72*, 650–654.

General materials and methods

Melting points were measured by a melting point instrument and are uncorrected. NMR spectra were recorded with 400 or 500 MHz spectrometers in $CDCl_3$ as a solvent at room temperature unless otherwise noted. Chemical shifts (δ) are given in ppm relative to TMS (^{1}H), external 85% H_3PO_4 (^{31}P), or residual $CHCl_3$ (^{13}C) as a reference. All ^{13}C and ^{31}P NMR spectra were recorded with the use of broadband proton decoupling. The coupling constants J are given in Hz, and the peak patterns are indicated as follows: s, singlet; d, doublet, t, triplet; q, quartet; m, multiplet. High-resolution mass spectra (HRMS), electrospray ionization (ESI) were measured with an orbitrap mass spectrometer. Optical rotation was measured on a polarimeter. The enantiomeric excess values were determined by HPLC equipped with a chiral stationary phase column. Unless otherwise noted, all reagents were used without further purification.

Preparation of aminophosphine oxide 9.

In a manner similar to [30], to the solution of *n*-propylamine (0.99 mL, 12 mmol) in THF (40 mL) at −80 °C was added slowly *n*-BuLi in hexane (7.1 mL, 12 mmol, 1.7 M). After phosphine oxide **8** (2.71 g, 8.0 mmol) was added at room temperature, the stirring was continued for 18 h at room temperature. The mixture was diluted with ether and quenched with sat. NH_4Cl aq. The organic layer was washed with brine, dried over $MgSO_4$, and concentrated under reduced pressure. The residue was purified by silica gel chromatography (hexane/EtOAc 2:1).

(3-Methoxy-2-(propylamino)phenyl)diphenylphosphine oxide (**9**): 186.0 mg, 0.51 mmol, 6%, yellow solid; mp 83-85 °C; ^{1}H NMR (400 MHz, $CDCl_3$) δ: 7.67-7.62 (m, 4H), 7.56-7.51 (m, 2H), 7.47-7.42 (m, 4H), 6.94 (dd, J = 1.0 and 8.0 Hz, 1H), 6.66 (td, J = 3.6 and 7.9 Hz, 1H), 6.44 (ddd, J = 1.4 and 7.7 and 13.9 Hz, 1H), 6.13 (s, br, 1H), 3.82 (s, 3H), 3.07 (t, J = 7.2 Hz, 2H), 1.29 (sex, J = 7.3 Hz, 2H), 0.74 (t, J = 7.4 Hz, 3H); ^{13}C NMR (101 MHz, $CDCl_3$) δ: 151.4 (d, $J_{C\text{-}P}$ = 11.7 Hz), 145.1 (d, $J_{C\text{-}P}$ = 5.4 Hz), 132.9 (d, $J_{C\text{-}P}$ = 104.6 Hz), 132.0 (d, $J_{C\text{-}P}$ = 9.8 Hz) × 4, 131.8 (d, $J_{C\text{-}P}$ = 2.9 Hz) × 2, 128.4 (d, $J_{C\text{-}P}$ = 12.3 Hz) × 4, 125.6 (d, $J_{C\text{-}P}$ = 11.5 Hz) × 2, 118.0 (d, $J_{C\text{-}P}$ = 15.7 Hz), 117.7 (d, $J_{C\text{-}P}$ = 105.3 Hz), 116.0 (d, $J_{C\text{-}P}$ = 2.2 Hz), 55.8, 48.9, 23.9, 11.4; ^{31}P NMR (162 MHz, $CDCl_3$) δ 35.3; HRMS (ESI-orbitrap-MS) *m*/*z* calcd for $C_{22}H_{24}O_2NP$+H 366.1617 found 366.1613; EI-MS m/z (rel intensity) 365 (M^+, 48).

Preparation of aminophosphine 10.

In a manner similar to [30], to a mixture of phosphine oxide **9** (73.3 mg, 0.2 mmol) and triethylamine (0.42 mL, 3 mmol) in *m*-xylene (4 mL) was added trichlorosilane (0.20 mL, 2 mmol) at 0 °C under an Ar atmosphere. The mixture was stirred at 120 °C for 24 h. After being cooled to room temperature, the reaction mixture was diluted with $CHCl_3$ and quenched

with 2 M of NaOH aq. solution. The organic layer was washed with brine, dried over $MgSO_4$, and concentrated under reduced pressure. The residue was purified by silica gel chromatography (hexane/EtOAc 40:1).

2-(Diphenylphosphaneyl)-6-methoxy-*N*-propylaniline (**10**): 59.1 mg, 0.17 mmol, 84%, white solid; mp 76-78 °C; ^{1}H NMR (400 MHz, $CDCl_3$) δ: 7.32–7.27 (m, 10H), 6.84–6.75 (m, 2H), 6.44 (ddd, J = 1.5 and 4.5 and 7.6 Hz, 1H), 4.17 (s, br, 1H), 3.83 (s, 3H), 3.09 (t, J = 7.2 Hz, 2H), 1.42 (sex, J = 7.3 Hz, 2H), 0.80 (t, J = 7.4 Hz, 3H); ^{13}C NMR (101 MHz, $CDCl_3$) δ: 151.2 (d, $J_{C\text{-}P}$ = 5.7 Hz), 142.6 (d, $J_{C\text{-}P}$ = 21.5 Hz), 136.6 (d, $J_{C\text{-}P}$ = 9.2 Hz) × 2, 133.6 (d, $J_{C\text{-}P}$ = 19.5 Hz) × 4, 128.52 × 2, 128.38 (d, $J_{C\text{-}P}$ = 6.8 Hz) × 4, 126.52, 126.48 (d, $J_{C\text{-}P}$ = 9.0 Hz), 120.9 (d, $J_{C\text{-}P}$ = 2.2 Hz), 111.9, 55.5, 50.6 (d, $J_{C\text{-}P}$ = 5.3 Hz), 23.7, 11.4 (d, $J_{C\text{-}P}$ = 1.1 Hz); ^{31}P NMR (162 MHz, $CDCl_3$) δ −19.3; HRMS (ESI-orbitrap-MS) *m*/*z* calcd for $C_{22}H_{24}ONP$+H 350.1668 found 350.1664; EI-MS m/z (rel intensity) 349 (M^+, 56).

Preparation of cinnamoyl amide (±)-7.

To the solution of aminophosphine **10** (69.7 mg, 0.20 mmol) in THF (5 mL) was added pyridine (0.032 mL, 0.4 mmol), *N*,*N*-dimethyl-4-aminopyridine (9.8 mg, 0.08 mmol) and cinnamoyl chloride (67 mg, 0.4 mmol) at room temperature. The reaction mixture was stirred at 40 °C. After 48 h, the mixture was quenched with water and diluted with diethyl ether. The organic layer was washed with water and brine, and dried over $MgSO_4$. The filtrate was concentrated and the residue was purified by column chromatography (hexane/EtOAc 4:1).

(±)-*N*-(2-(Diphenylphosphaneyl)-6-methoxyphenyl)-*N*-propylcinnamamide ((±)-**7**): 88.4 mg, 0.184 mmol, 92% as a white solid; mp 136-138 °C; ^{1}H NMR (400 MHz, $CDCl_3$) δ 7.38 (d, J = 15.5 Hz, 1H), 7.33-7.18 (m, 11H), 7.11-7.00 (m 5H), 6.96 (dd, J = 1.0 and 8.2 Hz, 1H), 6.77 (ddd, J = 1.1 and 2.9 and 7.7 Hz, 1H), 5.75 (d, J = 15.5 Hz, 1H), 4.08 (ddd, J = 5.5 and 11.0 and 13.4 Hz, 1H), 3.72 (s, 3H), 3.48-3.40 (m, 1H), 1.72-1.63 (m, 1H), 1.56-1.47 (m, 1H), 0.85 (t, J = 7.4 Hz, 3H); ^{13}C NMR (101 MHz, $CDCl_3$) δ 166.8 (d, $J_{C\text{-}P}$ = 0.5 Hz), 156.3 (d, $J_{C\text{-}P}$ = 5.9 Hz), 140.3, 139.8 (d, $J_{C\text{-}P}$ = 14.7 Hz), 136.5 (d, $J_{C\text{-}P}$ = 12.0 Hz), 135.3, 135.0 (d, $J_{C\text{-}P}$ = 11.8 Hz), 134.7 (d, $J_{C\text{-}P}$ = 25.2 Hz), 134.4 (d, $J_{C\text{-}P}$ = 21.9 Hz) × 2, 134.2 (d, $J_{C\text{-}P}$ = 19.3 Hz) × 2, 129.2, 129.0, 128.8, 128.37 (d, $J_{C\text{-}P}$ = 0.2 Hz) × 2, 128.310 (d, $J_{C\text{-}P}$ = 4.4 Hz) × 2, 128.308, 128.18 × 2, 127.6 (d, $J_{C\text{-}P}$ = 0.2 Hz) × 2, 126.2 (d, $J_{C\text{-}P}$ = 1.1 Hz), 118.9, 112.3, 55.5, 51.5 (d, $J_{C\text{-}P}$ = 7.4 Hz), 20.7 (d, $J_{C\text{-}P}$ = 1.8 Hz), 11.6 (d, $J_{C\text{-}P}$ = 0.4 Hz); ^{31}P NMR (162 MHz, $CDCl_3$) δ −16.9; HPLC (Daicel CHIRALCEL® OD-H, 0.46 ϕ × 25 cm; hexane : 2-propanol = 98 : 2, 0.80 mL/min, UV 280 nm) t_R = 23.8 min (CD : λ_{ext} ($\Delta_\varepsilon \varepsilon$) 280 (−)) t_R = 32.1 min (CD : λ_{ext} ($\Delta_\varepsilon \varepsilon$) 280 (+)); HRMS (ESI-orbitrap-MS) *m*/*z* calcd for $C_{31}H_{30}O_2NP$ + H 480.2087 found 480.2085; EI-MS m/z (rel intensity) 479 (M^+, 19).

Optical resolution of (±)-7.

HPLC resolution of (±)-**7** (56 mg, 0.117 mmol) dissolved in hexane (80 mL) was carried out by successive injections of 2 mL on a CHIRALCEL® OD-H (1.0 × 25 cm). A solution of hexane/2-propanol 98:2 was used as the eluent working at a flow rate of 3.0 mL/min and with UV monitoring at 280 nm. (−)-**7** and (+)-**7** were obtained by concentration of fractions respectively.

(−)-*N*-(2-(Diphenylphosphaneyl)-6-methoxyphenyl)-*N*-propylcinnamamide ((−)-**7**): 23.16 mg, 48 mmol, 41%, 99% ee; $[\alpha]^{20}_{D}$ = –207.9 (*c* 1.07, $CHCl_3$); orange solid; 86-87 °C; ^{1}H NMR (400 MHz, $CDCl_3$) δ: 7.37 (d, J = 15.4 Hz, 1H), 7.39-7.20 (m, 11H), 7.12-7.01 (m, 5H), 6.97 (dd, J = 8.3 and 1.1 Hz, 1H), 6.76 (ddd, J = 7.7, 3.0 and 1.3 Hz, 1H), 5.72 (d, J = 15.6 Hz, 1H), 4.07 (ddd, J = 5.5, 11.0 and 13.4 Hz, 1H), 3.74 (s, 3H), 3.47-3.39 (m, 1H), 1.70-1.62 (m, 1H), 1.54-1.45 (m, 1H), 0.85 (t, J = 7.4 Hz, 3H); ^{13}C NMR (126 MHz, $CDCl_3$) δ: 166.9 (d, $J_{C\text{-}P}$ = 0.7 Hz), 156.4 (d, $J_{C\text{-}P}$ = 5.9 Hz), 140.4, 140.0 (d, $J_{C\text{-}P}$ = 14.9 Hz), 136.6 (d, $J_{C\text{-}P}$ = 11.7 Hz), 135.4, 135.1 (d, $J_{C\text{-}P}$ = 11.3 Hz), 134.8 (d, $J_{C\text{-}P}$ = 25.4 Hz), 134.5 (d, $J_{C\text{-}P}$ = 21.5 Hz)×2, 133.3 (d, $J_{C\text{-}P}$ = 19.3 Hz)×2, 129.2, 129.1, 128.9, 128.5 (d, $J_{C\text{-}P}$ = 0.2 Hz) ×2, 128.4×(2 + 1), 128.3×2, 127.7 (d, $J_{C\text{-}P}$ = 0.2 Hz)×2, 126.2 (d, $J_{C\text{-}P}$ = 1.0 Hz), 119.0, 112.3, 55.6, 51.6 (d, $J_{C\text{-}P}$ = 7.3 Hz), 20.8 (d, $J_{C\text{-}P}$ = 1.2 Hz), 11.7 (d, $J_{C\text{-}P}$ = 0.2 Hz); ^{31}P NMR (162 MHz, $CDCl_3$) δ: -16.9; HPLC (Daicel CHIRALPAK® OD-H, 0.46 cmφ× 25 cm; Hex : 2-PrOH = 98 : 2, 0.8 mL/min, UV = 254 nm) t_R = 19.1 min (major, *R*), 26.6 min (minor, *S*); HRMS (ESI-orbitrap-MS) *m*/*z* calcd for $C_{31}H_{30}O_2NP$+H 480.2087 found 480.2076.

(+)-*N*-(2-(Diphenylphosphaneyl)-6-methoxyphenyl)-*N*-propylcinnamamide ((+)-**7**): 22.34 mg, 0.046 mmol, 40%, 99% ee; $[\alpha]^{20}_{D}$ = +205.3 (*c* 1.00, $CHCl_3$); orange solid; 86-87 °C; ^{1}H NMR (400 MHz, $CDCl_3$) δ: 7.37 (d, J = 15.5 Hz, 1H), 7.33-7.20 (m, 11H), 7.12-7.01 (m, 5H), 6.97 (dd, J = 8.2 and 1.1 Hz, 1H), 6.76 (ddd, J = 7.7, 3.0 and 1.2 Hz, 1H), 5.74 (d, J = 15.4 Hz, 1H), 4.07 (ddd, J = 13.4, 11.0 and 5.6 Hz, 1H), 3.74 (s, 3H), 3.46-3.39 (m, 1H), 1.71-1.62 (m, 1H), 1.54-1.46 (m, 1H), 0.85 (t, J = 7.5 Hz, 3H); ^{13}C NMR (126 MHz, $CDCl_3$) δ: 166.9 (d, $J_{C\text{-}P}$ = 0.2 Hz), 156.4 (d, $J_{C\text{-}P}$ = 5.9 Hz), 140.4, 140.0 (d, $J_{C\text{-}P}$ = 14.7 Hz), 136.6 (d, $J_{C\text{-}P}$ = 12.0 Hz), 135.4, 135.1 (d, $J_{C\text{-}P}$ = 11.3 Hz), 134.8 (d, $J_{C\text{-}P}$ = 25.4 Hz), 134.5 (d, $J_{C\text{-}P}$ = 22.5 Hz)×2, 133.3 (d, $J_{C\text{-}P}$ = 19.3 Hz)×2, 129.2, 129.1, 128.9, 128.5 (d, $J_{C\text{-}P}$ = 0.2 Hz) ×2, 128.4×(2 + 1), 128.3×2, 127.7 (d, $J_{C\text{-}P}$ = 0.2 Hz)×2, 126.2 (d, $J_{C\text{-}P}$ = 0.7 Hz), 119.0, 112.3, 55.6, 51.6 (d, $J_{C\text{-}P}$ = 6.9 Hz), 20.8 (d, $J_{C\text{-}P}$ = 2.0 Hz), 11.7 (d, $J_{C\text{-}P}$ = 0.2 Hz); ^{31}P NMR (162 MHz, $CDCl_3$) δ: -16.9; HPLC (Daicel CHIRALPAK® OD-H, 0.46 cmφ× 25 cm; Hex : 2-PrOH = 98 : 2, 0.8 mL/min, UV = 254 nm) t_R = 20.0 min (minor, *R*), 26.0 min (major, *S*); HRMS (ESI-orbitrap-MS) *m*/*z* calcd for $C_{31}H_{30}O_2NP$+H 480.2087 found 480.2081.

General procedure for the palladium-catalyzed allylic alkylation with isatin.

To a mixture of (a*R*)-(−)-**6** or (−)-**7** (12 μmol), $[Pd(C_3H_5)Cl]_2$ (1.85 mg, 5 μmol) (Pd = 10 mol %), isatin or substituted isatin (0.1 mmol), (*E*)-1,3-diphenyl-2-propenyl acetate (**12**, (50.4 mg, 0.20 mmol) or (*E*)-1,3-diphenyl-2-propenyl pivalate (**14**, 58.8 mg, 0.2 mmol) or (*E*)-1,3-di(*p*-chlorophenyl)-2-propenyl acetate (**15**, 64.24 mg, 0.2 mmol), and Na_3PO_4 (32.8 mg, 0.2 mol) was added $PhCF_3$ (0.5 mL) at 25 °C under an Ar atmosphere. After stirring for 48 h at 25 °C, the reaction mixture was quenched with water and diluted with $CHCl_3$. The organic layer was washed with water and brine, and dried over $MgSO_4$. The filtrate was concentrated under reduced pressure and the residue was purified by column chromatography (elution with *n*-hexane/EtOAc 4/1 and 100% of CH_2Cl_2).

(*S*,*E*)-1-(1,3-Diphenylallyl)indoline-2,3-dione ((*S*)-**13a**) (Table 1, entry 12 and Scheme 2): 28.38 mg, 0.084 mmol, 84%, 95% ee; $[\alpha]^{20}_D$ = –177.4 (*c* 0.10, $CHCl_3$); orange solid; mp 131-133 °C; ^1H NMR (400 MHz, $CDCl_3$) δ: 7.64-7.62 (m, 1H), 7.44-7.26 (m, 11H), 7.06 (td, *J* = 7.6 and 0.8 Hz, 1H), 6.82-6.71 (m, 2H), 6.67 (d, *J* = 8.0 Hz, 1H), 6.42 (d, *J* = 6.4 Hz, 1H); ^{13}C NMR (101 MHz, $CDCl_3$) δ:183.2, 158.1, 149.7, 137.9, 136.6, 135.7, 135.7, 129.0×2, 128.7×2, 128.4, 128.1, 127.0×2, 126.7×2, 125.5, 123.6, 122.8, 118.0, 113.0, 56.7; HPLC (Daicel CHIRALPAK® IA-3, 0.46 cmφ× 25 cm; Hex : 2-PrOH = 95 : 5, 1.00 mL/min, UV = 254 nm) t_R = 21.5 min (major, *S*), 25.5 min (minor, *R*); HRMS (ESI-orbitrap-MS) m/z calcd for $C_{23}H_{17}O_2N$+Na 362.1152, found 362.1145.

(*S*,*E*)-1-(1,3-Diphenylallyl)indoline-2,3-dione ((*S*)-**13a**) (Table 1, entry 13): 16.95 mg, 0.050 mmol, 50%, 86% ee; $[\alpha]^{20}_D$ = –111.4 (*c* 0.10, $CHCl_3$); orange solid; 120-122 °C; ^1H NMR (400 MHz, $CDCl_3$) δ: 7.65-7.27 (m, 1H), 7.44-7.26 (m, 11H), 7.07 (td, *J* = 7.5 and 0.8 Hz, 1H), 6.82-6.71 (m, 2H), 6.67 (d, *J* = 8.2 Hz, 1H), 6.43 (d, *J* = 6.6 Hz, 1H); ^{13}C NMR (126 MHz, $CDCl_3$) δ:183.2, 158.1, 149.8, 138.0, 136.7, 135.8, 135.8, 129.0×2, 128.7×2, 128.5, 128.2, 127.0×2, 126.7×2, 125.5, 123.6, 122.9, 118.1, 113.0, 56.8; HPLC (Daicel CHIRALPAK® IA-3, 0.46 cm φ× 25 cm; Hex : 2-PrOH = 95 : 5, 1.00 mL/min, UV = 254 nm) t_R = 20.8 min (major, *S*), 25.2 min (minor, *R*).

(*S*,*E*)-1-(1,3-Diphenylallyl)indoline-2,3-dione ((*S*)-**13a**) (Table 1, entry 14): 270.93 mg, 0.80 mmol, 80%, 94% ee; $[\alpha]^{20}_D$ = –193.8 (*c* 0.11, $CHCl_3$); orange solid; mp 131-133 °C; ^1H NMR (400 MHz, $CDCl_3$) δ: 7.64-7.62 (m, 1H), 7.44-7.26 (m, 11H), 7.06 (td, *J* = 7.6 and 0.8 Hz, 1H), 6.82-6.71 (m, 2H), 6.67 (d, *J* = 8.0 Hz, 1H), 6.42 (d, *J* = 6.4 Hz, 1H); ^{13}C NMR (101 MHz, $CDCl_3$) δ:183.2, 158.1, 149.7, 137.9, 136.6, 135.7, 135.7, 129.0×2, 128.7×2, 128.4, 128.2, 127.0×2, 126.7×2, 125.5, 123.6, 122.8, 118.0, 113.0, 56.7; HPLC (Daicel CHIRALPAK® IA-3, 0.46 cmφ× 25 cm; Hex : 2-PrOH = 95 : 5, 1.00 mL/min, UV = 254 nm) t_R = 21.3 min (major, *S*), 24.8 min (minor, *R*).

(*S*,*E*)-4-Chloro-1-(1,3-diphenylallyl)indoline-2,3-dione ((*S*)-**13b**): 33.32 mg, 0.089 mmol, 89%, 94% ee; $[\alpha]^{20}_D$ = –107.3 (*c* 0.13, $CHCl_3$); orange solid; mp 63-65°C; ^1H NMR (400 MHz, $CDCl_3$) δ: 7.44-7.27 (m, 11H), 7.00 (d, *J* = 8.3, 1H), 6.80-6.69 (m, 2H), 6.51 (d, *J* = 8.0 Hz,

1H), 6.44 (d, *J* = 7.2 Hz, 1H); ^{13}C NMR (101 MHz, $CDCl_3$) *δ*:180.1, 157.2, 150.8, 138.1, 136.3, 136.0, 135.6, 133.9, 129.1×2, 128.7×2, 128.5, 128.3, 126.9×2, 126.7×2, 125.2, 122.5, 115.1, 111.2, 56.9; HPLC (Daicel CHIRALPAK® IA-3, 0.46 cmφ× 25 cm; Hex : EtOH = 97 : 3, 1.00 mL/min, UV = 254 nm) t_R = 30.5 min (minor, *S*), 33.6 min (major, *R*); HRMS (ESI-orbitrap-MS) m/z calcd for $C_{23}H_{16}O_2NCl$+Na 396.0762, found 396.0755.

(*S*,*E*)-5-Methyl-1-(1,3-diphenylallyl)indoline-2,3-dione ((*S*)-**13c**): 34.89 mg, 0.099 mmol, 99%, 92% ee; $[\alpha]^{20}_D$ = –152.8 (*c* 0.10, $CHCl_3$); dark orange solid; mp 167-169 °C ^{1}H NMR (500 MHz, $CDCl_3$) *δ*: 7.44-7.27 (m, 11H), 7.19 (ddd, *J* = 8.0, 1.8 and 0.6 Hz, 1H), 6.80-6.70 (m, 2H), 6.55 (d, *J* = 8.2 Hz, 1H), 6.40 (d, *J* = 6.8 Hz, 1H), 2.27 (s, 3H); ^{13}C NMR (126 MHz, $CDCl_3$) *δ*:183.5, 158.2, 147.6, 138.4, 136.8, 135.8, 135.6, 133.5, 129.0×2, 128.7×2, 128.4, 128.1, 127.0×2, 126.7×2, 125.8, 123.0, 118.1, 112.9, 56.7, 20.6; HPLC (Daicel CHIRALPAK® IA-3, 0.46 cmφ× 25 cm; Hex : 2-PrOH = 95 : 5, 0.50 mL/min, UV = 254 nm) t_R = 42.2 min (major, *S*), 45.9 min (minor, *R*); HRMS (ESI-orbitrap-MS) m/z calcd for $C_{24}H_{19}O_2N$+Na 376.1308, found 376.1299.

(*S*,*E*)-5-Chloro-1-(1,3-diphenylallyl)indoline-2,3-dione((*S*)-**13d**): 18.22 mg, 0.049 mmol, 49%, 95% ee; $[\alpha]^{20}_D$ = –161.7 (*c* 0.13, $CHCl_3$); orange solid; 67-69 °C; ^{1}H NMR (400 MHz, $CDCl_3$) *δ*: 7.59 (dd, 2.3 and 0.3 Hz, 1H), 7.44-7.27 (m, 11H), 6.80-6.66 (m, 2H), 6.61 (dd, *J* = 8.5 and 0.4 Hz, 1H), 6.42 (d, *J* = 7.0 Hz, 1H); ^{13}C NMR (101 MHz, $CDCl_3$) *δ*:182.3, 157.6, 148.0, 137.3, 136.2, 136.0, 135.5, 129.6, 129.1×2, 128.8×2, 128.6, 128.4, 127.0×2, 126.7×2, 125.4, 122.3, 119.0, 114.3, 56.8; HPLC (Daicel CHIRALPAK® IA+IA-3, 0.46 cmφ× 25 cm; Hex : 2-PrOH = 93 : 7, 0.50 mL/min, UV = 254 nm) t_R = 62.3 min (major, *S*), 68.0 min (minor, *R*); HRMS (ESI-orbitrap-MS) m/z calcd for $C_{23}H_{16}O_2NCl$+Na 396.0762, found 396.0761.

(*S*,*E*)-6-Fluoro-1-(1,3-diphenylallyl)indoline-2,3-dione ((*S*)-**13g**): 30.38 mg, 0.085 mmol, 85% as an orange viscous oil, 92% ee; $[\alpha]^{20}_D$ = –127.4 (*c* 0.10, $CHCl_3$); ^{1}H NMR (400 MHz, $CDCl_3$) *δ*: 7.66 (dd, *J* = 8.3 and 5.7, 1H), 7.45-7.28 (m, 10H), 6.81-6.67 (m, 3H), 6.41-6.35 (m, 2H); ^{13}C NMR (101 MHz, $CDCl_3$) *δ*:181.1, 168.4 (d, *J* = 260.2 Hz), 152.3 (d, *J* = 13.7 Hz), 136.2, 136.0, 135.5, 129.2×2, 128.7×2, 128.6, 128.4, 128.2, 128.1, 127.0×2, 126.7×2, 122.3, 114.5 (d, *J* = 2.9 Hz), 110.7 (d, *J* = 23.5 Hz), 101.8 (d, *J* = 28.4 Hz), 57.0; HPLC (Daicel CHIRALPAK® AD-3, 0.46 cmφ× 25 cm; Hex : 2-PrOH = 95 : 5, 0.70 mL/min, UV = 254 nm) t_R = 36.5 min (major, *S*), 41.0 min (minor, *R*); HRMS (ESI-orbitrap-MS) m/z calcd for $C_{23}H_{16}O_2NF$+Na 380.1057, found 380.1050.

(*S*,*E*)-6-Chloro-1-(1,3-diphenylallyl)indoline-2,3-dione ((*S*)-**13h**): 33.95 mg, 0.091 mmol, 91%, 94% ee; $[\alpha]^{20}_D$ = –163.5 (*c* 0.10, $CHCl_3$); yellow solid; mp 115-117 °C; ^{1}H NMR (400 MHz, $CDCl_3$) *δ*: 7.56 (d, *J* = 8.0 Hz, 1H), 7.46-7.28 (m, 10H), 7.05 (dd, 8.1 and 1.6 Hz, 1H), 6.82-6.70 (m, 2H), 6.65 (d, *J* = 1.4 Hz, 1H), 6.38 (d, *J* = 7.2 Hz, 1H); ^{13}C NMR (101 MHz, $CDCl_3$) *δ*:181.7, 158.0, 150.7, 144.3, 136.2, 136.1, 135.6, 129.2×2, 128.7×2, 128.6, 128.4, 126.9×2, 126.8×2, 126.4, 124.0, 122.4, 116.4, 113.4, 57.1; HPLC (Daicel CHIRALPAK® IA-3, 0.46 cmφ× 25 cm; Hex : 2-PrOH = 95 : 5, 0.50 mL/min, UV = 254 nm) t_R = 36.5 min (major,

S), 41.0 min (minor, *R*); HRMS (ESI-orbitrap-MS) m/z calcd for $C_{23}H_{16}O_2NCl$+Na 396.0762, found 396.0756.

(*S*,*E*)-6-Bromo-1-(1,3-diphenylallyl)indoline-2,3-dione ((*S*)-**13i**) : 33.03 mg, 0.079 mmol, 79%, 93% ee; $[\alpha]^{20}_D$ = –93.7 (*c* 0.09, $CHCl_3$); yellow solid; mp 129-130 °C; ^{1}H NMR (500 MHz, $CDCl_3$) *δ*: 7.50-7.29 (m, 11H), 7.34 (dd, *J* = 7.9 and 1.5 Hz, 1H), 6.83-6.78 (m, 2H), 6.74-6.70 (m, 1H), 6.37 (d, *J* = 7.3 Hz, 1H); ^{13}C NMR (126 MHz, $CDCl_3$) *δ*:182.0, 157.9, 150.6, 136.2, 136.1, 135.6, 133.2, 129.2×2, 128.8×2, 128.6, 128.5, 127.0, 127.0×2, 126.8×2, 126.4, 122.4, 116.7, 116.2, 57.1; HPLC (Daicel CHIRALPAK® AD-3, 0.46 cmφ× 25 cm; Hex : 2-PrOH = 85 : 15, 1.00 mL/min, UV = 254 nm) t_R = 13.3 min (major, *S*), 20.9 min (minor, *R*); HRMS (ESI-orbitrap-MS) m/z calcd for $C_{23}H_{16}O_2NBr$+Na 440.0257, found 440.0255.

(*S*,*E*)-6-Methoxy-1-(1,3-diphenylallyl)indoline-2,3-dione ((*S*)-**13j**): 24.45 mg, 0.066 mmol, 66%, 93% ee; $[\alpha]^{20}_D$ = –151.7 (*c* 0.11, $CHCl_3$); orange solid; mp 129-130 °C; ^{1}H NMR (500 MHz, $CDCl_3$) *δ*: 7.60 (d, *J* = 8.4 Hz, 1H), 7.44-7.27 (m, 10H), 6.79-6.69 (m, 2H), 6.50 (d, *J* = 8.4 and 2.1 Hz, 1H), 6.40 (d, *J* = 7.0 Hz, 1H), 6.14 (d, *J* = 2.0 Hz, 1H), 3.73 (s, 3H); ^{13}C NMR (126 MHz, $CDCl_3$) *δ*:180.5, 167.6, 159.5, 152.1, 136.8, 135.8, 135.5, 129.0×2, 128.7×2, 128.4, 128.2, 128.0, 127.1×2, 126.7×2, 123.0, 111.8, 107.8, 100.3, 56.6, 55.9; HPLC (Daicel CHIRALPAK® IA-3, 0.46 cmφ× 25 cm; Hex : 2-PrOH = 95 : 5, 1.00 mL/min, UV = 254 nm) t_R = 37.9 min (major, *S*), 42.6 min (minor, *R*); HRMS (ESI-orbitrap-MS) m/z calcd for $C_{24}H_{19}O_3N$+Na 392.1257, found 392.1253.

(*S*,*E*)-7-Chloro-1-(1,3-diphenylallyl)indoline-2,3-dione ((*S*)-**13k**): 31.77 mg, 0.085 mmol, 85%, 67% ee; $[\alpha]^{20}_D$ = +37.0 (*c* 0.10, $CHCl_3$); dark orange solid; mp 135-136 °C; ^{1}H NMR (500 MHz, $CDCl_3$) *δ*: 7.61 (dd, *J* = 7.3 and 1.2 Hz, 1H), 7.52-7.26 (m, 11H), 7.10-7.03 (m, 2H), 6.89 (d, *J* = 8.0 Hz, 1H), 6.79 (d, *J* = 15.9 Hz, 1H); ^{13}C NMR (126 MHz, $CDCl_3$) *δ*:182.2, 158.6, 146.4, 141.2, 138.7, 136.0, 135.6, 128.7×2, 128.7×2, 128.3, 127.6, 126.8×2, 126.2, 125.4, 124.9, 124.4, 121.2, 117.3, 59.3; HPLC (Daicel CHIRALPAK® IA+IA-3, 0.46 cmφ× 25 cm; Hex : 2-PrOH = 93 : 7, 0.5 mL/min, UV = 254 nm) t_R = 65.3 min (major, *S*), 73.0 min (minor, *R*); HRMS (ESI-orbitrap-MS) m/z calcd for $C_{23}H_{16}O_2NCl$+Na 396.0762, found 396.0763.

(*S*,*E*)-1-(1,3-Bis(4-chlorophenyl)allyl)indoline-2,3-dione ((*S*)-**13l**): 33.33 mg, 0.082 mmol, 82%, 92% ee; $[\alpha]^{20}_D$ = –113.3 (*c* 0.10, $CHCl_3$); orange solid; 162-163 °C; ^{1}H NMR (500 MHz, $CDCl_3$) *δ*: 7.65 (d, *J* = 6.9 Hz, 1H), 7.44-7.29 (m, 9H), 7.10 (td, *J* = 7.7 and 0.6 Hz, 1H), 6.76-6.63 (m, 3H), 6.34 (d, *J* = 7.4 Hz, 1H); ^{13}C NMR (126 MHz, $CDCl_3$) *δ*:182.9, 158.0, 159.5, 149.3, 138.1, 135.0, 134.9, 134.3, 134.0, 129.3×2, 128.9×2, 128.4×2, 128.0×2, 125.7, 123.9, 123.0, 118.1, 112.7, 56.3; HPLC (Daicel CHIRALPAK® AD-3, 0.46 cmφ× 25 cm; Hex : 2-PrOH = 90 : 10, 1.0 mL/min, UV = 254 nm) t_R = 37.5 min (major, *S*), 64.4 min (minor, *R*); HRMS (ESI-orbitrap-MS) m/z calcd for $C_{23}H_{15}O_2NCl_2$+H 408.0553, found 408.0543.

Synthesis of malononitrile (*S*)-16.

To the solution of (*S*)-**13a** (94% ee) (33.9 mg, 0.1 mmol) in DMF (1 mL) was added

malononitrile (6.7 mg, 0.1 mmol) and $FeCl_3$ (1.62 mg, 0.01 mmol) at room temperature. The reaction mixture was stirred at 50 °C and after 2 h, the mixture was quenched with water and diluted with ethyl acetate. The organic layer was washed with water and brine, and dried over $MgSO_4$. The filtrate was concentrated and the residue was purified by column chromatography (hexane/EtOAc 3:1).

(*S*,*E*)-2-(1,3-Diphenylallyl-2-oxoindolin-3-ylidene)malononitrile ((*S*)-**16**): 27.06 mg, 0.070 mmol, 70%, 94% ee; $[\alpha]^{20}_D$ = +336.7 (*c* 0.03, $CHCl_3$); orange solid; 86-87 °C; ^{1}H NMR (400 MHz, $CDCl_3$) δ: 8.15-8.13 (dd, *J* = 7.9 and 0.7 Hz, 1H), 7.43-7.27 (m, 11H), 7.08 (td, *J* = 7.7 and 0.9 Hz, 1H), 6.78-6.69 (m, 2H), 6.65 (d, *J* = 8.5 Hz, 1H), 6.39 (d, *J* = 6.9 Hz, 1H); ^{13}C NMR (126 MHz, $CDCl_3$) δ:162.5, 149.2, 145.3, 137.3, 136.3, 136.1, 135.5, 129.0×2, 128.7×2, 128.6, 128.3, 127.0×2, 126.9, 126.7×2, 123.7, 122.4, 118.6, 112.5, 112.3, 110.7, 82.7, 57.0; HPLC (Daicel CHIRALPAK® AD-H, 0.46 cmφ× 25 cm; Hex : EtOH = 90 : 10, 1.50 mL/min, UV = 254 nm) t_R = 13.8 min (major, *S*), 28.9 min (minor, *R*); HRMS (ESI-orbitrap-MS) m/z calcd for $C_{26}H_{17}ON_3$+Na 410.1264, found 410.1260.

(3-Methoxy-2-(propylamino)phenyl)diphenylphosphine oxide (9)

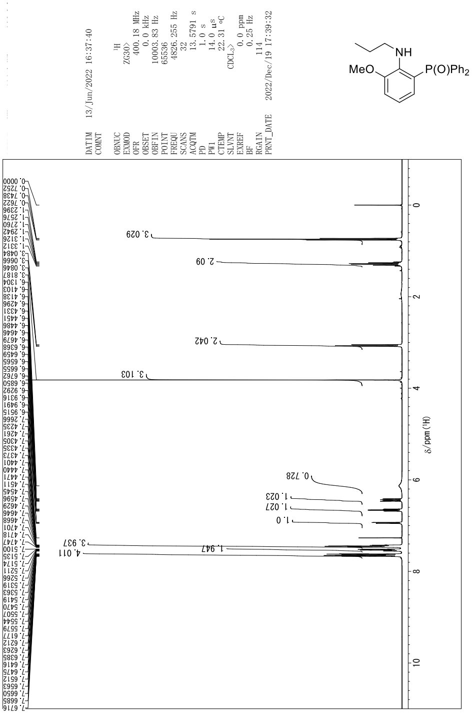

S12

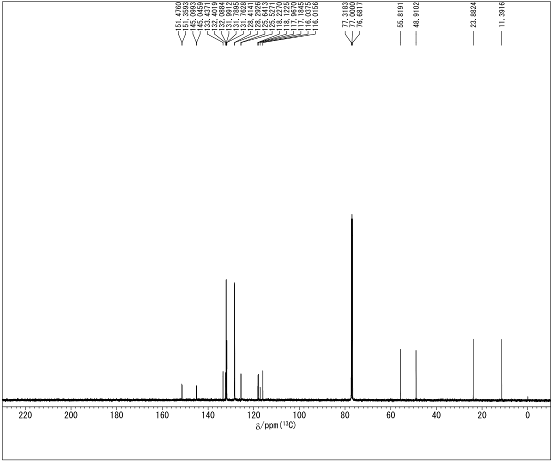

DATIM 13/Jun/2022 19:07:35
COMNT

OBNUC ^{13}C
EXMOD ZGPG30>
OFR 100.63 MHz
OBSET 0.0 kHz
OBFIN 12704.67 Hz
POINT 131072(ZeroFill
:×4)
FREQU 32051.28 Hz
SCANS 512
ACQTM 1.0224 s
PD 2.0 s
PW1 10.0 μs
CTEMP 23.01 °C
SLVNT $CDCL_3$>
EXREF 77.0 ppm
BF 0.9781 Hz
RGAIN 203
PRNT_DATE 2022/Dec/09 18:36:18

S13

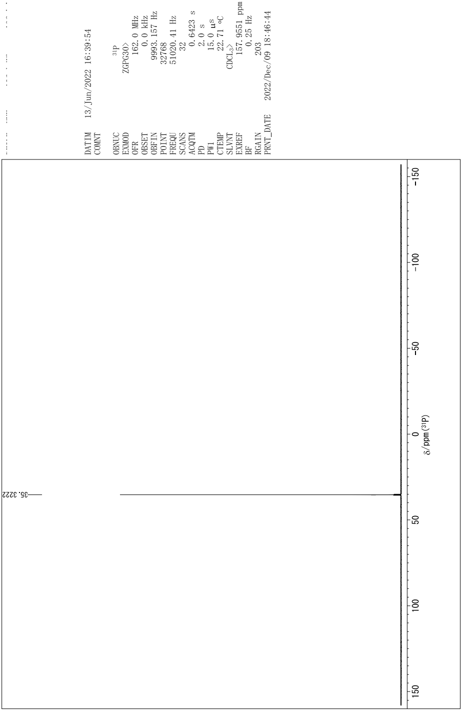
DATIM 13/Jun/2022 16:39:54
COMNT
OBNUC 31P
EXMOD ZGPG30>
OFR 162.0 MHz
OBSET 0.0 kHz
OBFIN 9993.157 Hz
POINT 32768
FREQU 51020.41 Hz
SCANS 32
ACQTM 0.6423 s
PD 2.0 s
PW1 15.0 us
CTEMP 22.71 °C
SLVNT CDCL3>
EXREF 157.9551 ppm
BF 0.25 Hz
RGAIN 203
PRNT_DATE 2022/Dec/09 18:46:44
35.3222
150
100
50
0
-50
-100
-150
δ/ppm(31P)

S14

2-(Diphenylphosphaneyl)-6-methoxy-*N*-propylaniline (10)

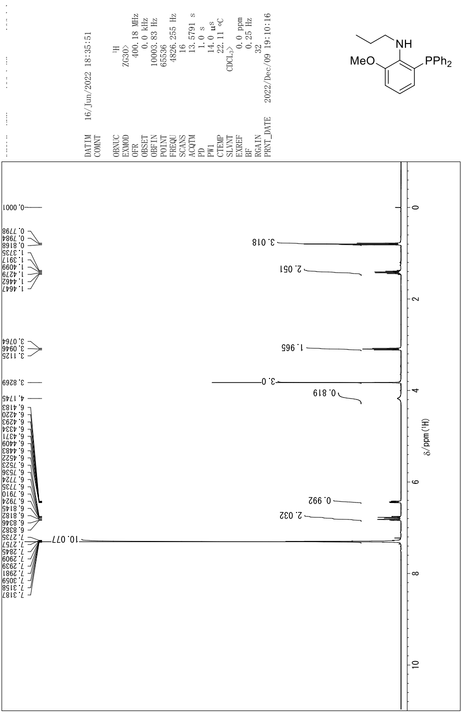

S15

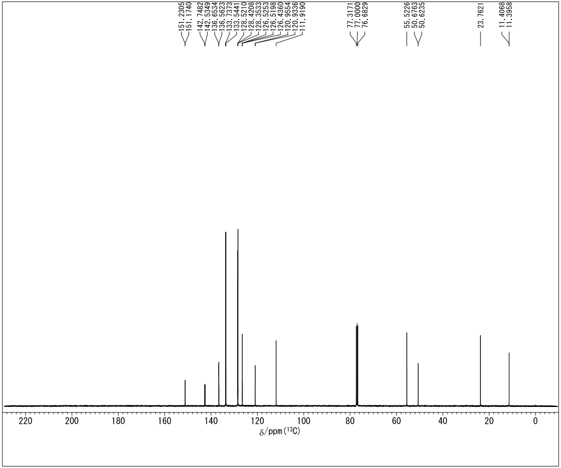

DATIM	16/Jun/2022 19:07:29
COMNT	
OBNUC	^{13}C
EXMOD	ZGPG30>
OFR	100.63 MHz
OBSET	0.0 kHz
OBFIN	11066.5 Hz
POINT	131072(ZeroFill :×4)
FREQU	24038.46 Hz
SCANS	512
ACQTM	1.3631 s
PD	2.0 s
PW1	10.0 μs
CTEMP	22.91 °C
SLVNT	$CDCL_3$>
EXREF	77.0 ppm
BF	0.7336 Hz
RGAIN	203
PRNT_DATE	2022/Dec/12 18:01:43

S16

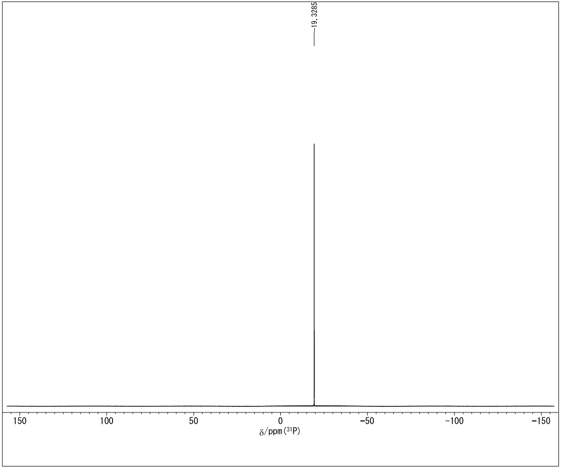

DATIM	16/Jun/2022 19:10:25
COMNT	
OBNUC	^{31}P
EXMOD	ZGPG30>
OFR	162.0 MHz
OBSET	0.0 kHz
OBFIN	−1.3506 Hz
POINT	131072(ZeroFill :×4)
FREQU	51020.41 Hz
SCANS	16
ACQTM	0.6423 s
PD	2.0 s
PW1	15.0 μs
CTEMP	22.61 °C
SLVNT	$CDCL_3$>
EXREF	−0.007 ppm
BF	1.557 Hz
RGAIN	203
PRNT_DATE	2022/Dec/12 18:12:46

S17

(±)-*N*-(2-(Diphenylphosphaneyl)-6-methoxyphenyl)-*N*-propylcinnamamide ((±)-7)

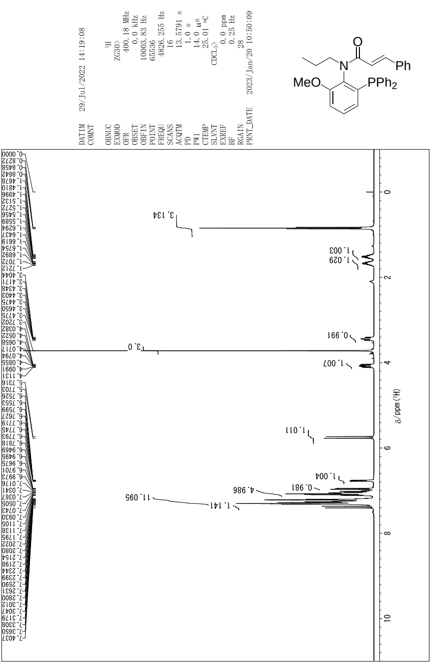

S18

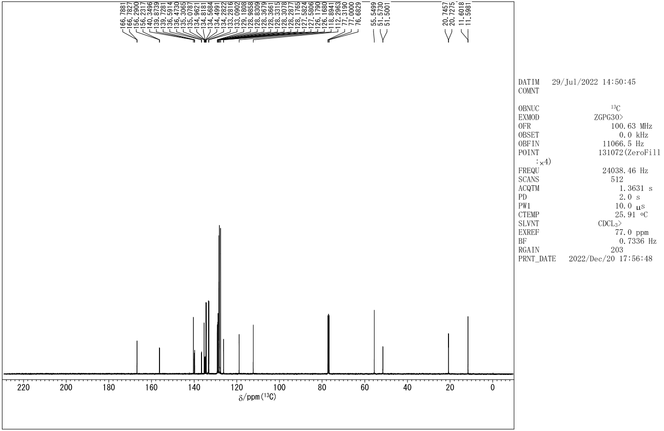

166.7881
166.7827
156.2900
156.2317
140.3496
139.8739
139.7281
136.5914
136.4730
135.3065
135.0787
134.9621
134.8181
134.5684
134.4991
134.2822
133.2816
133.0902
129.1808
128.9858
128.8309
128.3679
128.3661
128.3315
128.3078
128.2877
128.1765
127.5824
127.5806
126.1790
126.1680
118.8941
112.2963
77.3190
77.0000
76.6829
55.5499
51.5730
51.5001
20.7457
20.7275
11.6018
11.5981
220
200
180
160
140
120
100
80
60
40
20
0
δ/ppm(13C)
DATIM 29/Jul/2022 14:50:45
COMNT
OBNUC 13C
EXMOD ZGPG30>
OFR 100.63 MHz
OBSET 0.0 kHz
OBFIN 11066.5 Hz
POINT 131072(ZeroFill :×4)
FREQU 24038.46 Hz
SCANS 512
ACQTM 1.3631 s
PD 2.0 s
PW1 10.0 μs
CTEMP 25.91 °C
SLVNT CDCL3>
EXREF 77.0 ppm
BF 0.7336 Hz
RGAIN 203
PRNT_DATE 2022/Dec/20 17:56:48

S19

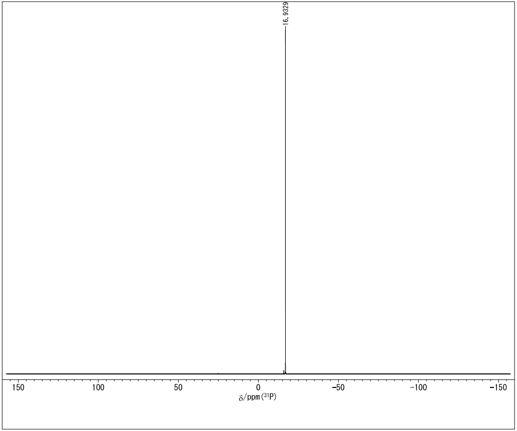

DATIM	29/Jul/2022 14:53:38
COMNT	
OBNUC	^{31}P
EXMOD	ZGPG30>
OFR	162.0 MHz
OBSET	0.0 kHz
OBFIN	−1.3506 Hz
POINT	131072(ZeroFill :×4)
FREQU	51020.41 Hz
SCANS	16
ACQTM	0.6423 s
PD	2.0 s
PW1	15.0 μs
CTEMP	25.61 °C
SLVNT	$CDCL_3$>
EXREF	−0.007 ppm
BF	1.557 Hz
RGAIN	203
PRNT_DATE	2022/Dec/12 20:16:48

S20

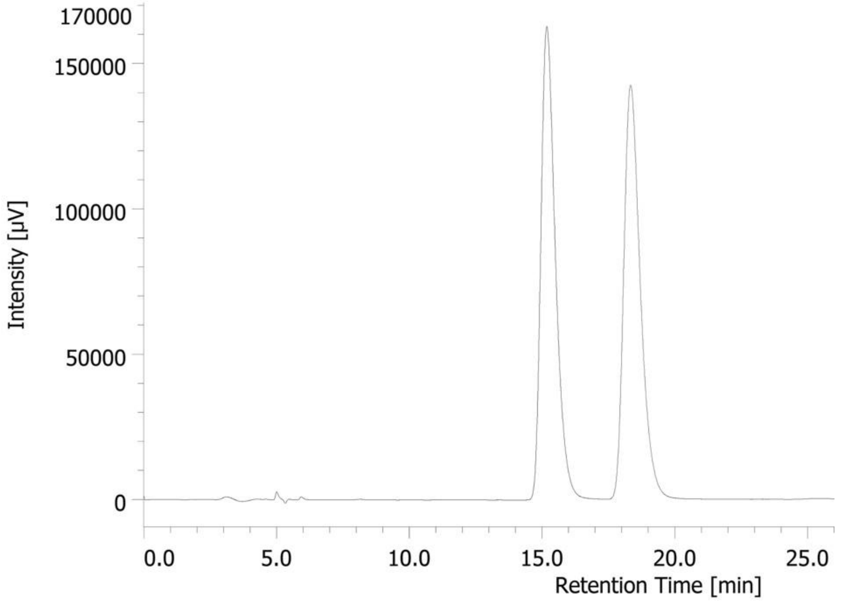
170000
150000
100000
50000
0
Intensity [µV]
0.0
5.0
10.0
15.0
20.0
25.0
Retention Time [min]

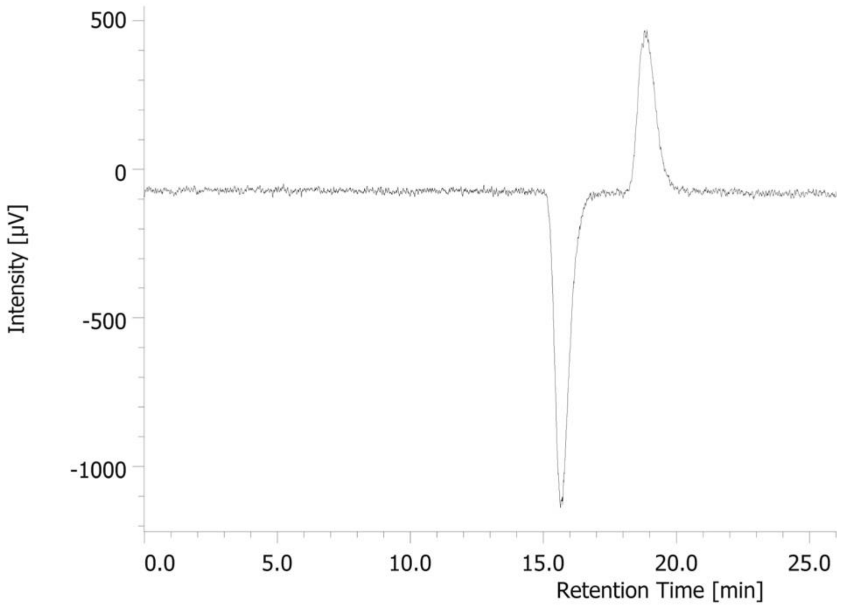
500
0
-500
-1000
Intensity [µV]
0.0
5.0
10.0
15.0
20.0
25.0
Retention Time [min]

(−)-*N*-(2-(Diphenylphosphaneyl)-6-methoxyphenyl)-*N*-propylcinnamamide ((−)-7)

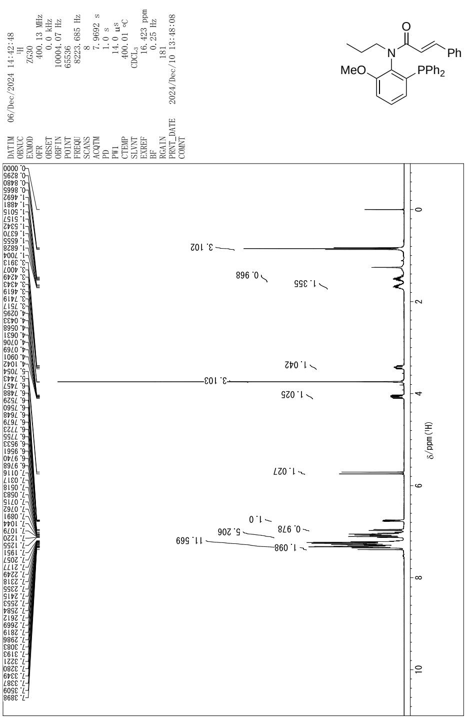

S22

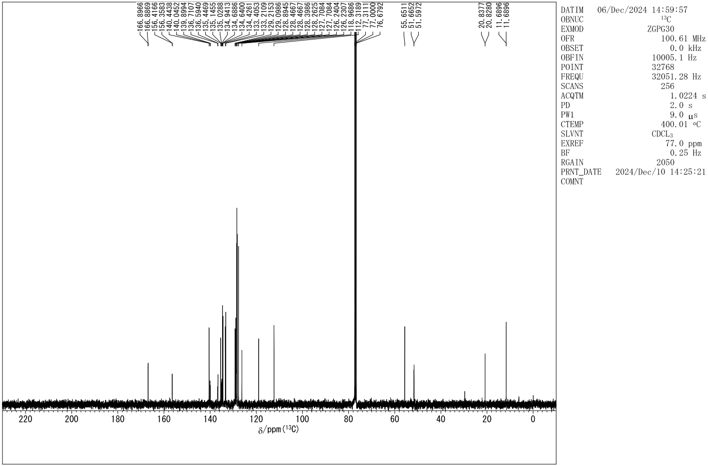
166.8966
166.8869
156.4166
156.3583
140.4438
140.0452
139.8994
136.7107
136.5940
135.4469
135.1455
135.0288
134.9413
134.6886
134.6400
134.4261
133.4053
133.2109
129.2153
129.0986
128.8945
128.4667
128.4667
128.3986
128.2625
127.7084
127.7084
126.2404
126.2307
118.9686
112.3189
77.3111
77.0000
76.6792
55.6511
51.6652
51.5972
20.8377
20.8280
11.6896
11.6896
220
200
180
160
140
120
100
80
60
40
20
0
δ/ppm(^{13}C)
DATIM 06/Dec/2024 14:59:57
OBNUC ^{13}C
EXMOD ZGPG30
OFR 100.61 MHz
OBSET 0.0 kHz
OBFIN 10005.1 Hz
POINT 32768
FREQU 32051.28 Hz
SCANS 256
ACQTM 1.0224 s
PD 2.0 s
PW1 9.0 us
CTEMP 400.01 °C
SLVNT CDCL$_3$
EXREF 77.0 ppm
BF 0.25 Hz
RGAIN 2050
PRNT_DATE 2024/Dec/10 14:25:21
COMNT

S23

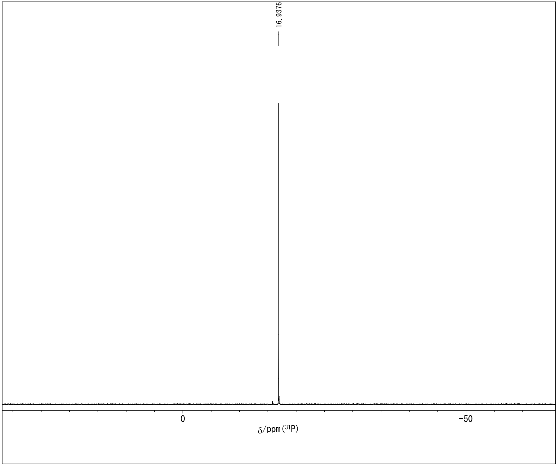

DATIM	06/Dec/2024 14:44:23
OBNUC	^{31}P
EXMOD	ZGPG30
OFR	161.98 MHz
OBSET	0.0 kHz
OBFIN	10001.57 Hz
POINT	32768
FREQU	51020.41 Hz
SCANS	16
ACQTM	0.6423 s
PD	2.0 s
PW1	9.0 μs
CTEMP	400.01 °C
SLVNT	$CDCL_3$
EXREF	157.4941 ppm
BF	0.25 Hz
RGAIN	2050
PRNT_DATE	2024/Dec/10 14:31:44
COMNT	

S24

Chromatogram

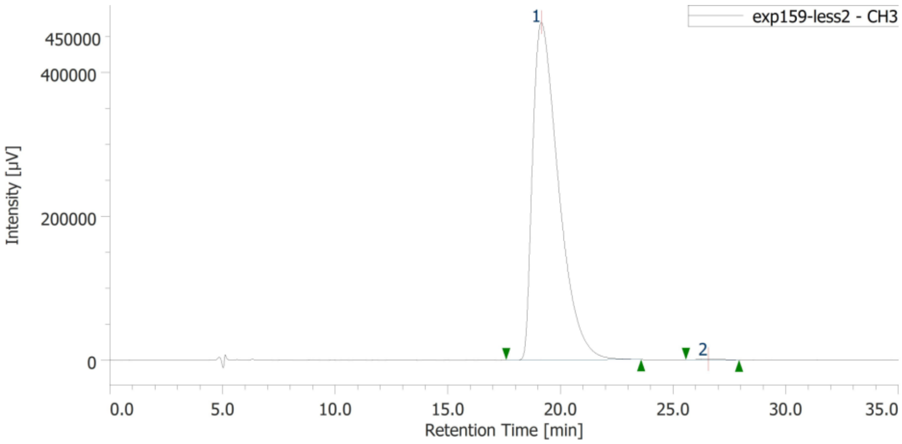

Chromatogram Information	
User Name	3385
Date Modified	2024/12/04 16:00:50
Description	OD-H, 0.8 mL/min, hex:2-pro=98:2
HPLC System Name	SYSTEM-3
Injection Date	2024/12/04 13:23:48
Volume	1.0 [μL]
Sample #	1
Project Name	2024system3
Acquisition Time	180.0 [min]
Acquisition Sequence	3-2290 nats
Control Method	2019system3
Peak ID Table	
Calibration Method	
Additional Information	

Channel & Peak Information Table	
Chromatogram Name	exp159-less2-CH3
Sample Name	
Channel Name	UV-2075
Sampling Interval	500 [msec]
Peak Method	(Manual)

#	Peak Name	CH	tR [min]	Area [μV·sec]	Height [μV]	Area%	Height%
1	Unknown	3	19.14	37566756	469042	99.85	99.8
2	Unknown	3	26.55	57792	887	0.1536	0.189

(+)-*N*-(2-(Diphenylphosphaneyl)-6-methoxyphenyl)-N-propylcinnamamide ((+)-7)

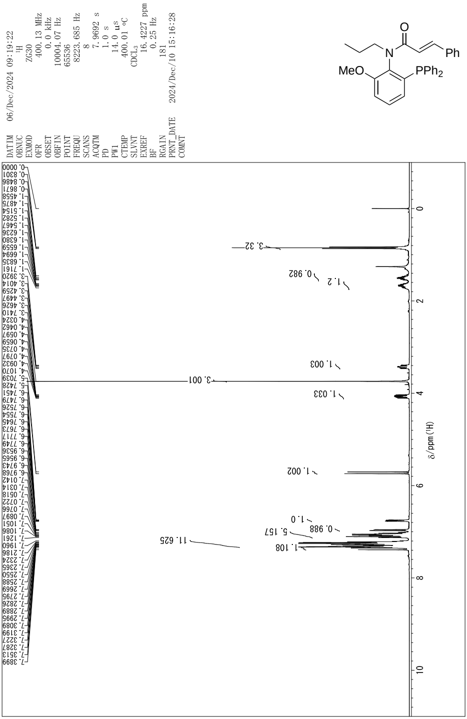

S26

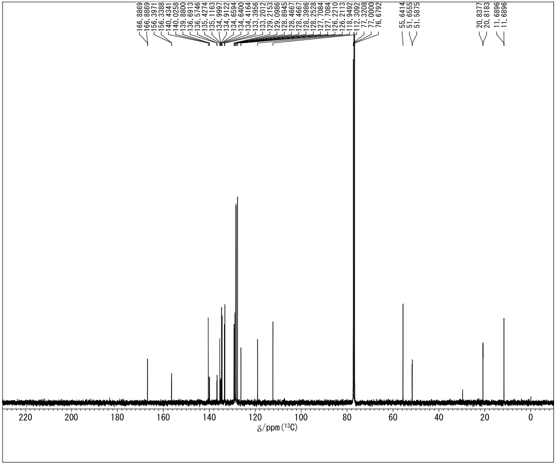

DATIM	06/Dec/2024 09:53:09
OBNUC	^{13}C
EXMOD	ZGPG30
OFR	100.61 MHz
OBSET	0.0 kHz
OBFIN	10005.1 Hz
POINT	32768
FREQU	32051.28 Hz
SCANS	512
ACQTM	1.0224 s
PD	2.0 s
PW1	9.0 μs
CTEMP	400.01 °C
SLVNT	$CDCL_3$
EXREF	77.0 ppm
BF	0.25 Hz
RGAIN	2050
PRNT_DATE	2024/Dec/10 15:49:40
COMNT	

S27

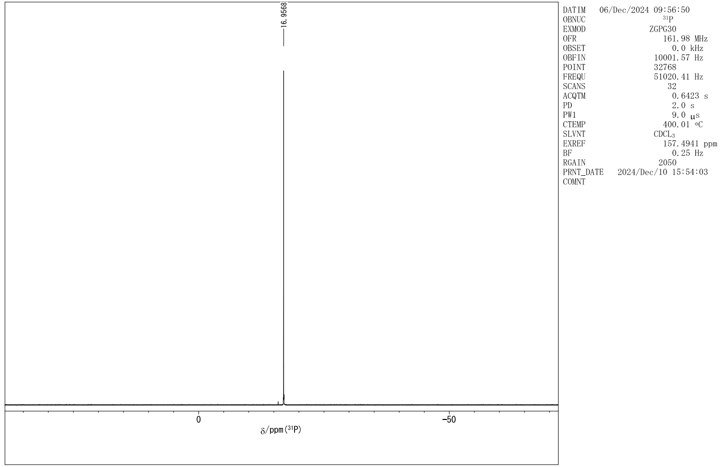
16.9568
0
−50
δ/ppm(31P)
DATIM 06/Dec/2024 09:56:50
OBNUC 31P
EXMOD ZGPG30
OFR 161.98 MHz
OBSET 0.0 kHz
OBFIN 10001.57 Hz
POINT 32768
FREQU 51020.41 Hz
SCANS 32
ACQTM 0.6423 s
PD 2.0 s
PW1 9.0 us
CTEMP 400.01 °C
SLVNT CDCL3
EXREF 157.4941 ppm
BF 0.25 Hz
RGAIN 2050
PRNT_DATE 2024/Dec/10 15:54:03
COMNT

S28

Chromatogram

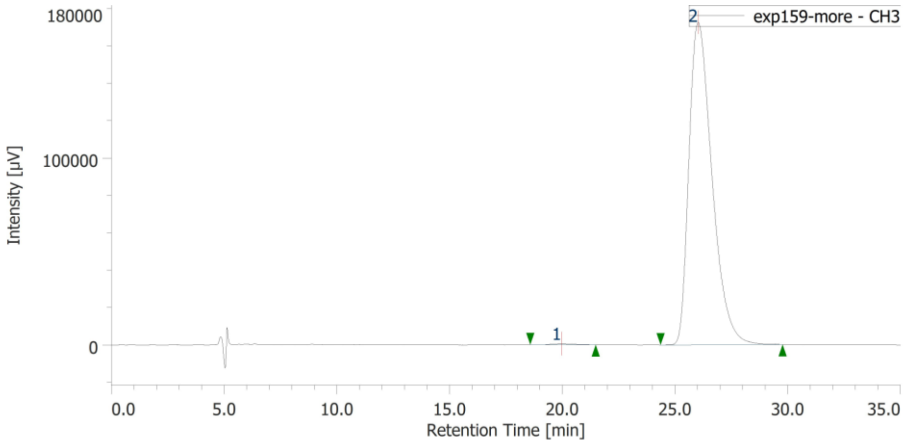

Chromatogram Information	
User Name	3385
Date Modified	2024/12/04 16:00:50
Description	OD−H, 0.8 mL/min, hex:2−pro=98:2
HPLC System Name	SYSTEM−3
Injection Date	2024/12/04 14:18:48
Volume	1.0 [µL]
Sample #	1
Project Name	2024system3
Acquisition Time	180.0 [min]
Acquisition Sequence	3−2291 nats
Control Method	2019system3
Peak ID Table	
Calibration Method	
Additional Information	

Channel & Peak Information Table	
Chromatogram Name	exp159−more−CH3
Sample Name	
Channel Name	UV−2075
Sampling Interval	500 [msec]
Peak Method	(Manual)

#	Peak Name	CH	tR [min]	Area [µV·sec]	Height [µV]	Area%	Height%
1	Unknown	3	19.98	32721	504	0.2659	0.291
2	Unknown	3	26.02	12274297	172422	99.73	99.7

(*S,E*)-1-(1,3-Diphenylallyl)indoline-2,3-dione ((*S*)-13a) (Table 1, entry 12 and Scheme 2)

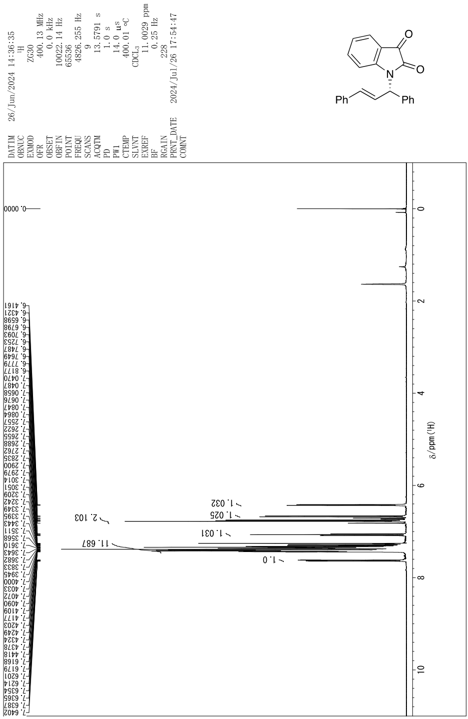

S30

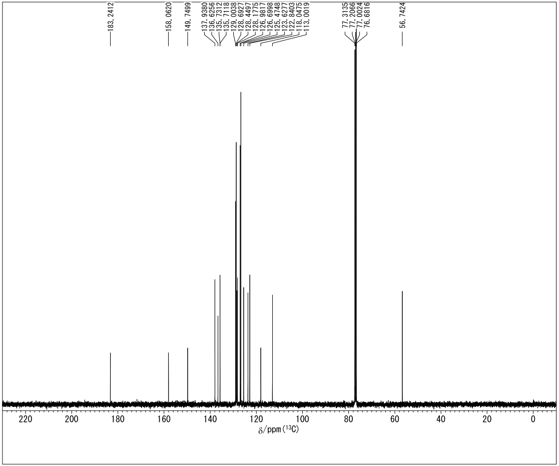

DATIM	26/Jun/2024 15:06:08
OBNUC	^{13}C
EXMOD	ZGPG30
OFR	100.61 MHz
OBSET	0.0 kHz
OBFIN	10005.1 Hz
POINT	32768
FREQU	32051.28 Hz
SCANS	512
ACQTM	1.0224 s
PD	2.0 s
PW1	9.0 μs
CTEMP	400.01 °C
SLVNT	$CDCL_3$
EXREF	77.0 ppm
BF	0.25 Hz
RGAIN	2050
PRNT_DATE	2024/Jul/26 17:57:36
COMNT	

S31

Chromatogram

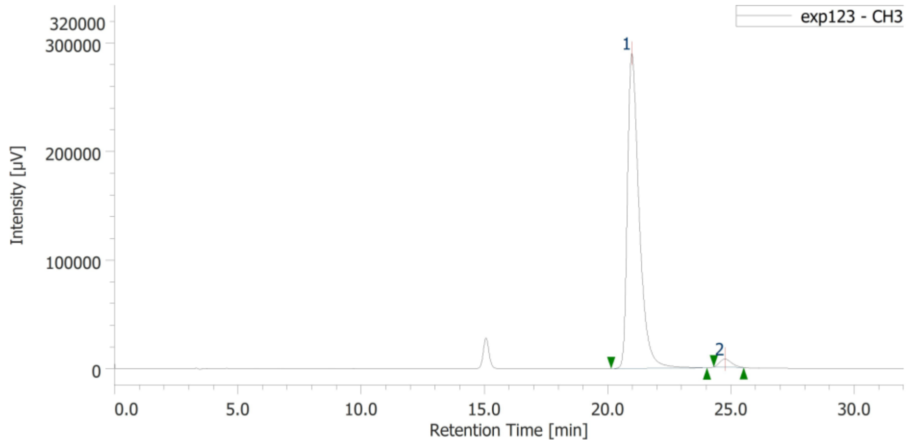

Chromatogram Information

User Name	3385
Date Modified	2024/06/13 8:39:38
Description	IA-3, 1.0 mL/min, hex:2-PrOH=95:5, UV = 254 nm, CD =270 nm
HPLC System Name	SYSTEM-3
Injection Date	2024/06/13 8:03:26
Volume	1.0 [μL]
Sample #	1
Project Name	2024system3
Acquisition Time	180.0 [min]
Acquisition Sequence	3-1974 nats
Control Method	2019system3
Peak ID Table	
Calibration Method	
Additional Information	

Channel & Peak Information Table

Chromatogram Name	exp123-CH3
Sample Name	
Channel Name	UV-2075
Sampling Interval	500 [msec]
Peak Method	(Manual)

#	Peak Name	CH	tR [min]	Area [μV·sec]	Height [μV]	Area%	Height%
1	Unknown	3	20.98	9594336	290077	97.46	97.5
2	Unknown	3	24.75	249909	7412	2.539	2.49

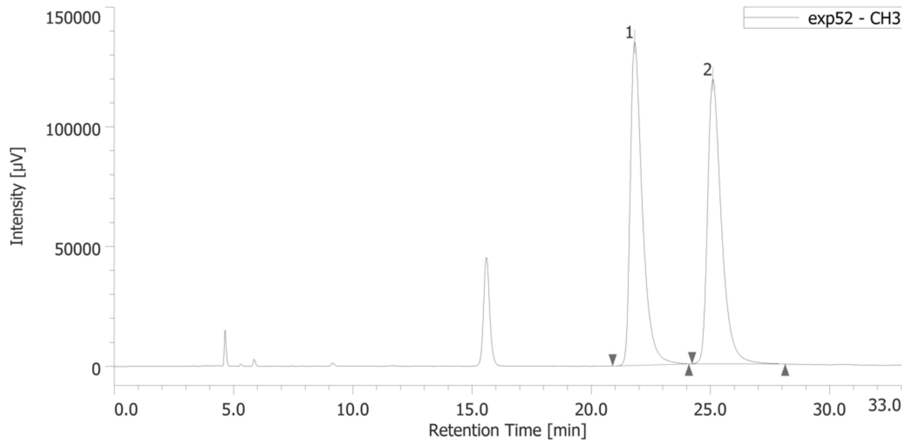

(*S,E*)-1-(1,3-Diphenylallyl)indoline-2,3-dione ((*S*)-13a) (Table 1, entry 13)

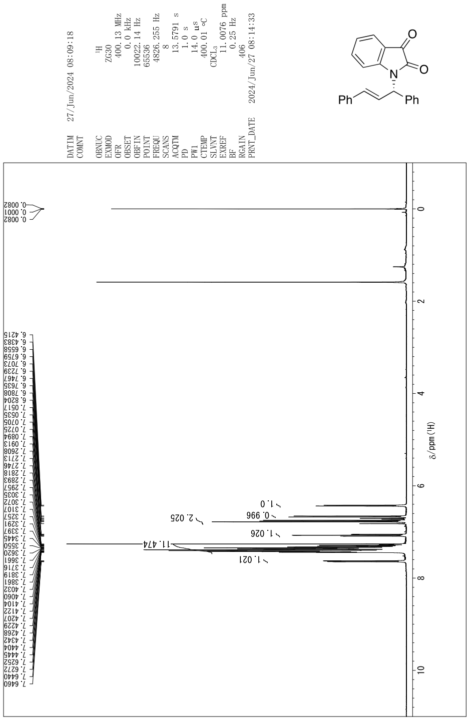

S33

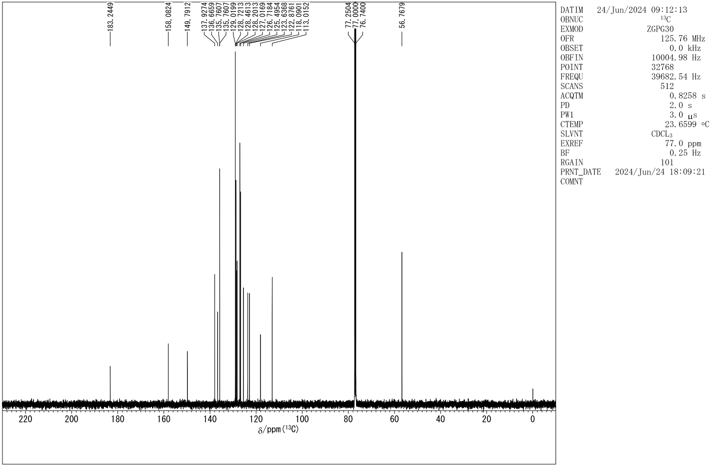
183.2449
158.0824
149.7912
137.9274
136.6659
135.7607
135.7607
129.0199
128.7213
128.4613
128.2013
127.0169
126.7184
125.4954
123.6368
122.8761
118.0901
113.0152
77.2504
77.0000
76.7400
56.7679
220 200 180 160 140 120 100 80 60 40 20 0
δ/ppm(13C)
DATIM 24/Jun/2024 09:12:13
OBNUC 13C
EXMOD ZGPG30
OFR 125.76 MHz
OBSET 0.0 kHz
OBFIN 10004.98 Hz
POINT 32768
FREQU 39682.54 Hz
SCANS 512
ACQTM 0.8258 s
PD 2.0 s
PW1 3.0 us
CTEMP 23.6599 °C
SLVNT CDCL3
EXREF 77.0 ppm
BF 0.25 Hz
RGAIN 101
PRNT_DATE 2024/Jun/24 18:09:21
COMNT

S34

Chromatogram

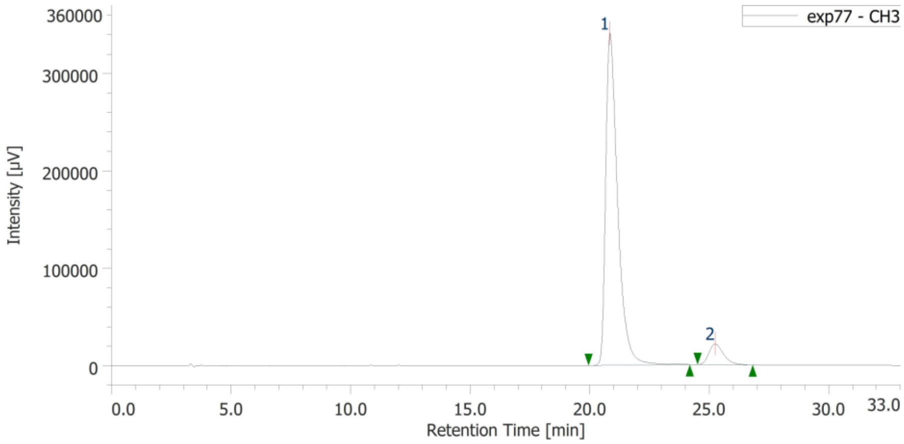

Chromatogram Information

User Name	3385
Date Modified	2024/06/24 9:51:55
Description	IA3, 1.0 mL/min, hex:2-PrOH=95:5, UV = 254 nm, CD =270 nm
HPLC System Name	SYSTEM-3
Injection Date	2024/06/24 7:35:13
Volume	1.0 [μL]
Sample #	1
Project Name	2024system3
Acquisition Time	180.0 [min]
Acquisition Sequence	3-2019 nats
Control Method	2019system3
Peak ID Table	
Calibration Method	
Additional Information	

Channel & Peak Information Table

Chromatogram Name	exp77-CH3
Sample Name	
Channel Name	UV-2075
Sampling Interval	500 [msec]
Peak Method	(Manual)

#	Peak Name	CH	tR [min]	Area [μV·sec]	Height [μV]	Area%	Height%
1	Unknown	3	20.84	11881358	340772	92.94	94.1
2	Unknown	3	25.24	902402	21376	7.059	5.90

(*S,E*)-1-(1,3-Diphenylallyl)indoline-2,3-dione ((*S*)-13a) (Table 1, entry 14)

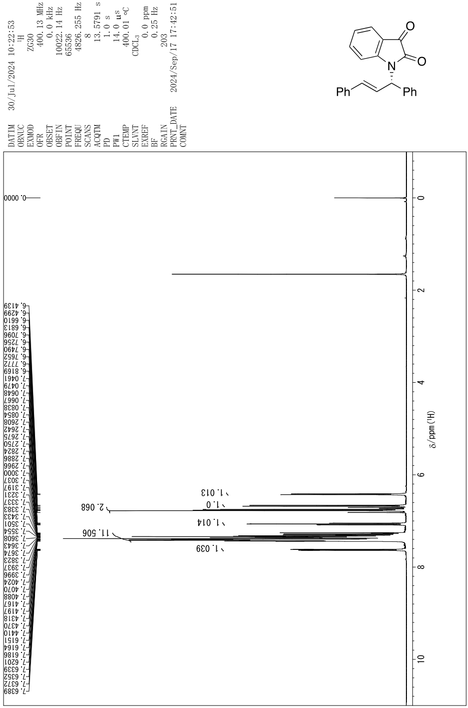

S36

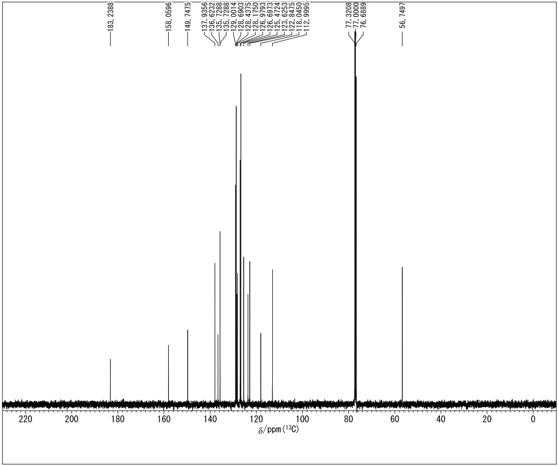

DATIM	30/Jul/2024 10:38:46
OBNUC	^{13}C
EXMOD	ZGPG30
OFR	100.61 MHz
OBSET	0.0 kHz
OBFIN	10005.1 Hz
POINT	32768
FREQU	32051.28 Hz
SCANS	256
ACQTM	1.0224 s
PD	2.0 s
PW1	9.0 μs
CTEMP	400.01 °C
SLVNT	$CDCL_3$
EXREF	77.0 ppm
BF	0.25 Hz
RGAIN	2050
PRNT_DATE	2024/Jul/30 11:06:53
COMNT	

S37

Chromatogram

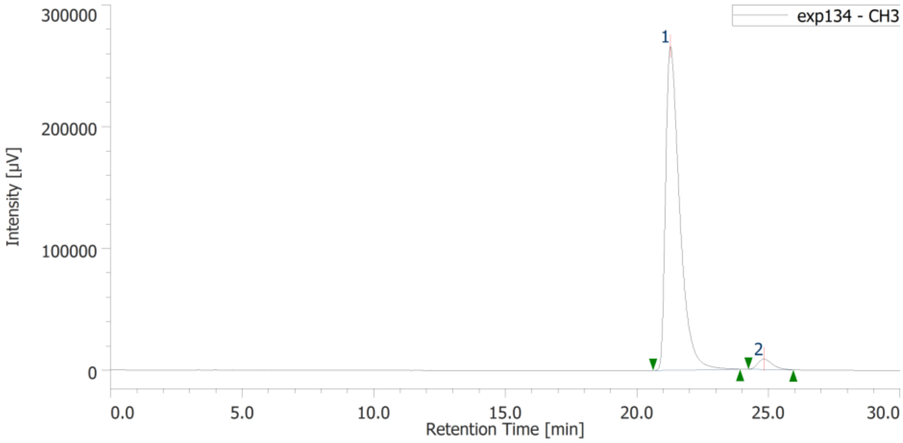

Chromatogram Information	
User Name	3385
Date Modified	2024/07/30 12:44:16
Description	IA3,1.0 mL/min, Hex:EtOH=95:5,UV254nm,CD270nm
HPLC System Name	HPLC-1
Injection Date	2024/07/30 12:13:54
Volume	1.00 [µL]
Sample #	1
Project Name	2024system1
Acquisition Time	180.0 [min]
Acquisition Sequence	1-2757 nats
Control Method	system1_2017
Peak ID Table	
Calibration Method	
Additional Information	

Channel & Peak Information Table	
Chromatogram Name	exp134-CH3
Sample Name	
Channel Name	UV-2075
Sampling Interval	500 [msec]
Peak Method	(Manual)

#	Peak Name	CH	tR [min]	Area [µV·sec]	Hight [µV]	Area%	Hight%
1	Unknown	3	21.275	9876317	265716	96.81	96.9
2	Unknown	3	24.825	325022	8498	3.186	3.10

(*S,E*)-4-Chloro-1-(1,3-diphenylallyl)indoline-2,3-dione ((*S*)-13b)

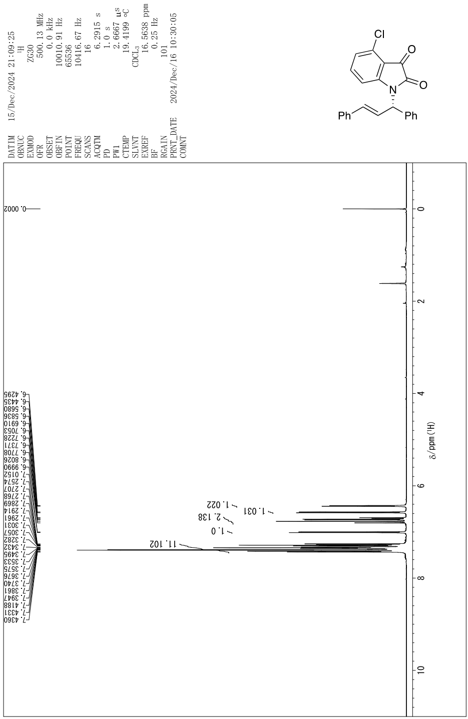

S39

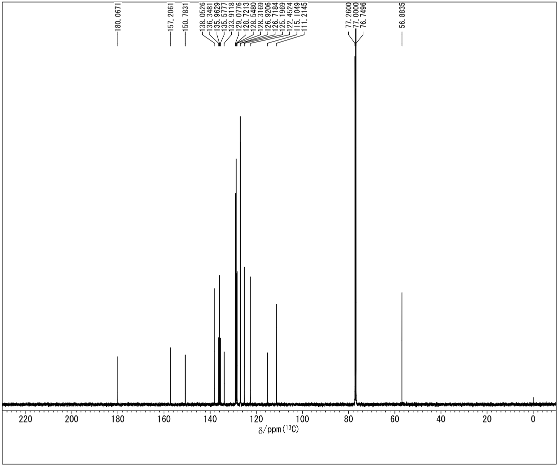

DATIM	15/Dec/2024 21:35:20
OBNUC	^{13}C
EXMOD	ZGPG30
OFR	125.76 MHz
OBSET	0.0 kHz
OBFIN	10004.98 Hz
POINT	32768
FREQU	39682.54 Hz
SCANS	512
ACQTM	0.8258 s
PD	2.0 s
PW1	3.0 μs
CTEMP	20.2718 °C
SLVNT	$CDCL_3$
EXREF	77.0 ppm
BF	0.25 Hz
RGAIN	101
PRNT_DATE	2024/Dec/16 10:35:43
COMNT	

S40

Chromatogram

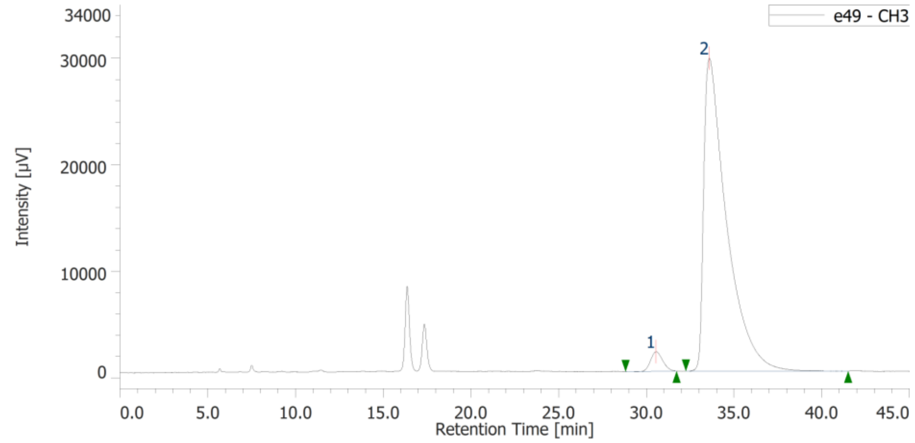

Chromatogram Information

User Name	3385
Date Modified	2023/04/25 17:49:30
Description	IA-3, 1.0 mL/min, Hex:EtOH=97:3
HPLC System Name	HPLC-1
Injection Date	2023/04/25 14:40:50
Volume	1.00 [μL]
Sample #	1
Project Name	2023system1
Acquisition Time	180.0 [min]
Acquisition Sequence	1-2346 nats
Control Method	system1_2017
Peak ID Table	
Calibration Method	
Additional Information	

Channel & Peak Information Table

Chromatogram Name	e49-CH3
Sample Name	
Channel Name	UV-2075
Sampling Interval	500 [msec]
Peak Method	(Manual)

#	Peak Name	CH	tR [min]	Area [μV·sec]	Height [μV]	Area%	Height%
1	Unknown	3	30.542	87904	1837	3.202	5.89
2	Unknown	3	33.583	2657174	29356	96.798	94.1

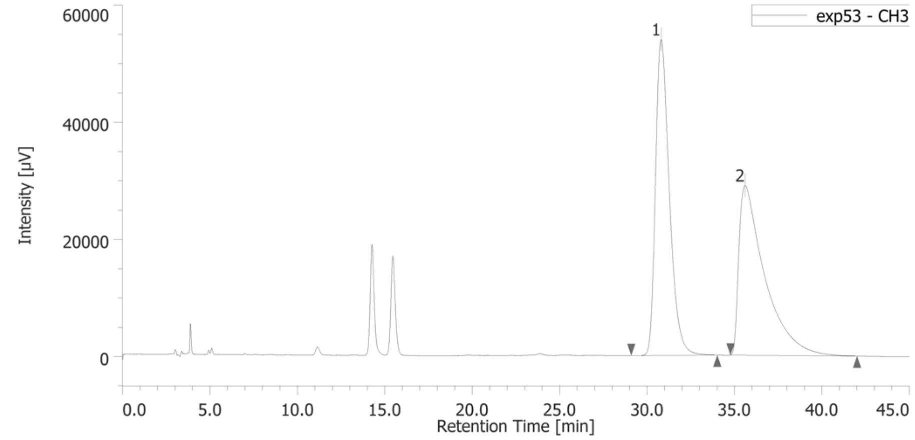

(*S,E*)-5-Methyl-1-(1,3-diphenylallyl)indoline-2,3-dione ((*S*)-13c)

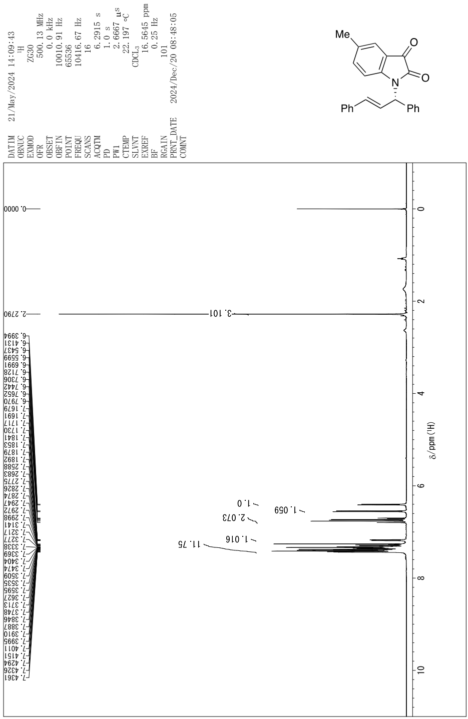

S42

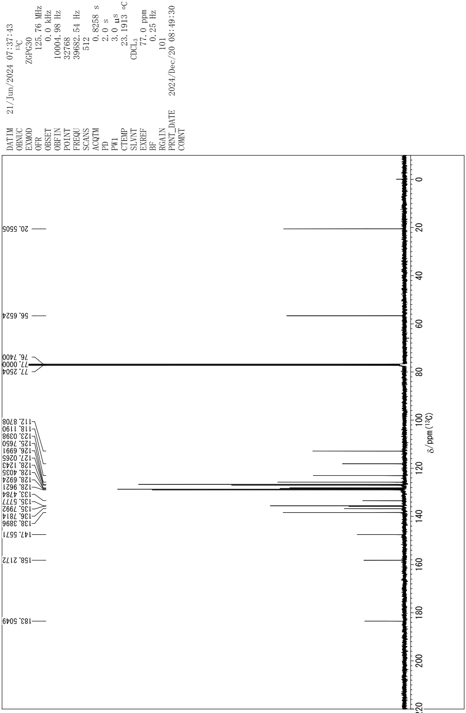
DATIM 21/Jun/2024 07:37:43
OBNUC 13C
EXMOD ZGPG30
OFR 125.76 MHz
OBSET 0.0 kHz
OBFIN 10004.98 Hz
POINT 32768
FREQU 39682.54 Hz
SCANS 512
ACQTM 0.8258 s
PD 2.0 s
PW1 3.0 us
CTEMP 23.1913 °C
SLVNT CDCL3
EXREF 77.0 ppm
BF 0.25 Hz
RGAIN 101
PRNT_DATE 2024/Dec/20 08:49:30
COMNT
20.5505
56.6524
76.7400
77.0000
77.2504
112.8708
118.1190
123.0398
125.7650
126.6991
127.0265
128.1243
128.4035
128.6924
128.9621
133.4784
135.5777
135.7992
136.7814
138.3896
147.5571
158.2172
183.5049
δ/ppm(13C)

S43

Chromatogram

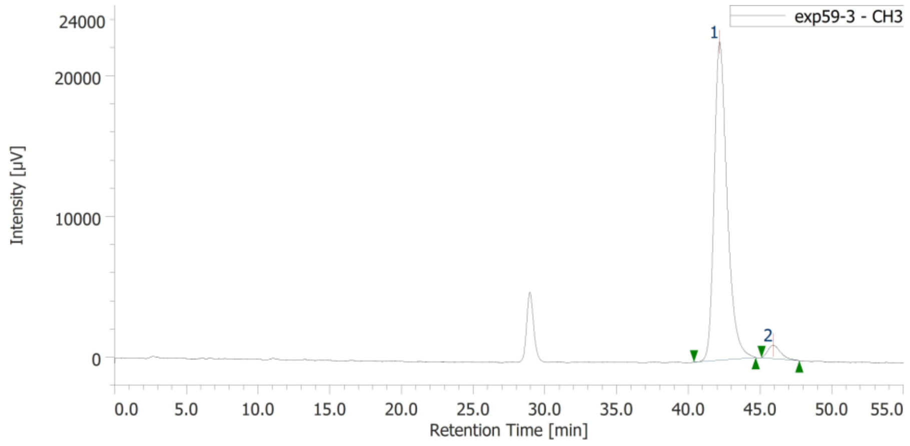

Chromatogram Information

User Name	3385
Date Modified	2024/06/13 10:37:25
Description	IA3, 0.5 mL/min, hex:2pro=95:5
HPLC System Name	SYSTEM-3
Injection Date	2023/06/20 13:10:06
Volume	1.0 [μL]
Sample #	1
Project Name	2023system3
Acquisition Time	180.0 [min]
Acquisition Sequence	3-1466 nats
Control Method	2019system3
Peak ID Table	
Calibration Method	
Additional Information	

Channel & Peak Information Table

Chromatogram Name	exp59-3-CH3
Sample Name	
Channel Name	UV-2075
Sampling Interval	500 [msec]
Peak Method	(Manual)

#	Peak Name	CH	tR [min]	Area [μV·sec]	Height [μV]	Area%	Height%
1	Unknown	3	42.17	1424779	22622	96.059	95.975
2	Unknown	3	45.91	58459	949	3.941	4.025

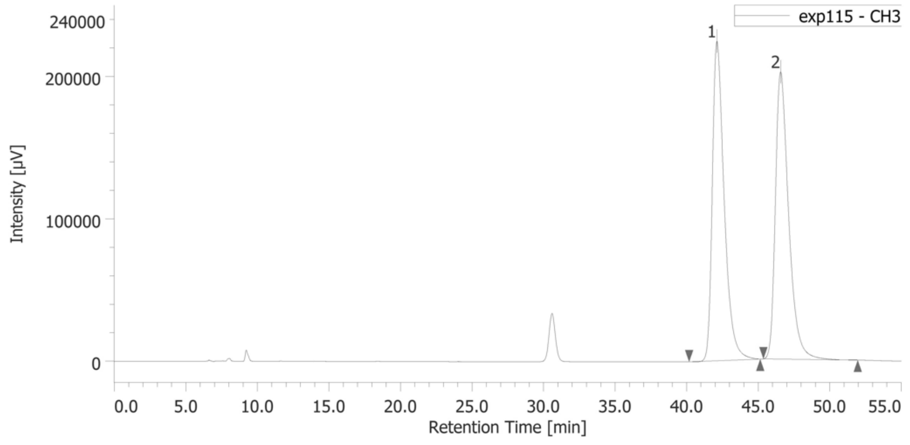

(*S,E*)-5-Chloro-1-(1,3-diphenylallyl)indoline-2,3-dione ((*S*)-13d)

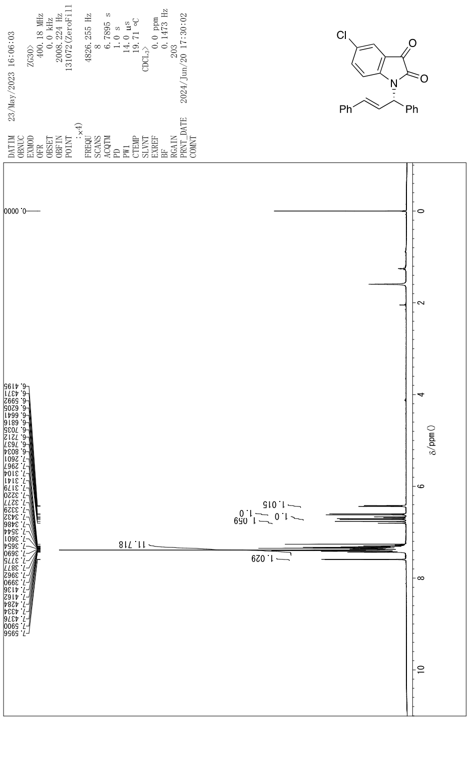

S45

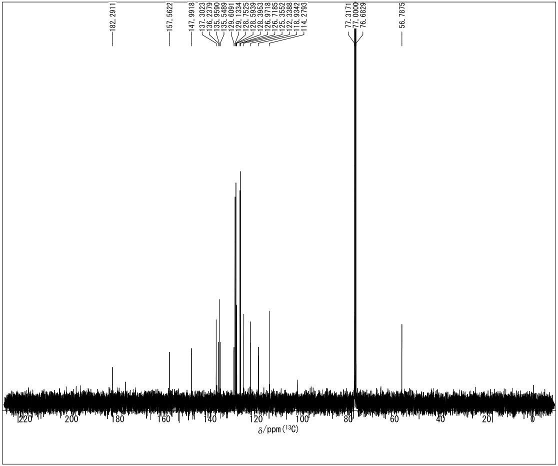

DATIM 23/May/2023 16:13:21
OBNUC ^{13}C
EXMOD ZGPG30>
OFR 100.63 MHz
OBSET 0.0 kHz
OBFIN 11066.5 Hz
POINT 131072(ZeroFill :x4)
FREQU 24038.46 Hz
SCANS 64
ACQTM 1.3631 s
PD 2.0 s
PW1 10.0 μs
CTEMP 20.41 °C
SLVNT $CDCL_3$>
EXREF 77.0 ppm
BF 0.7336 Hz
RGAIN 203
PRNT_DATE 2024/Jul/29 11:08:58
COMNT

S46

Chromatogram

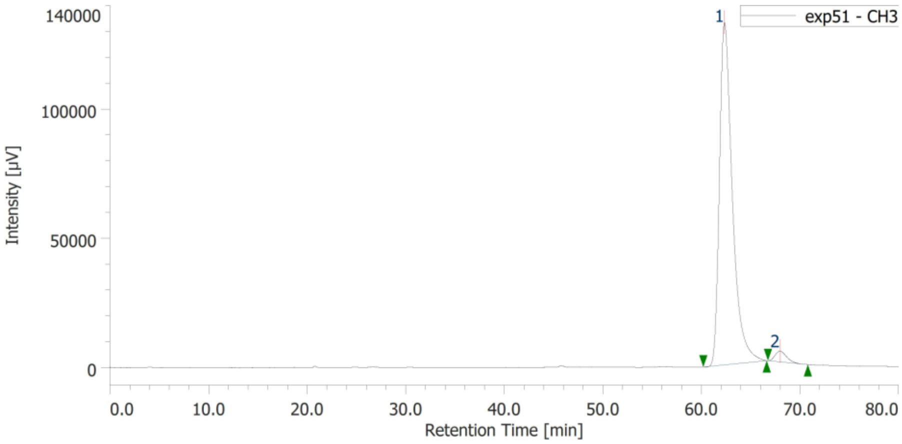

Chromatogram Information	
User Name	3385
Date Modified	2024/07/01 13:10:51
Description	IA-IA3, 0.5 mL/min, hex:2pro=93:7
HPLC System Name	SYSTEM-3
Injection Date	2023/05/23 12:26:47
Volume	1.0 [µL]
Sample #	1
Project Name	2023system3
Acquisition Time	180.0 [min]
Acquisition Sequence	3-1416 nats
Control Method	2019system3
Peak ID Table	
Calibration Method	
Additional Information	

Channel & Peak Information Table	
Chromatogram Name	exp51-CH3
Sample Name	
Channel Name	UV-2075
Sampling Interval	500 [msec]
Peak Method	(Manual)

#	Peak Name	CH	tR [min]	Area [µV·sec]	Height [µV]	Area%	Height%
1	Unknown	3	62.34	12061115	132492	97.284	96.970
2	Unknown	3	67.98	336727	4140	2.716	3.030

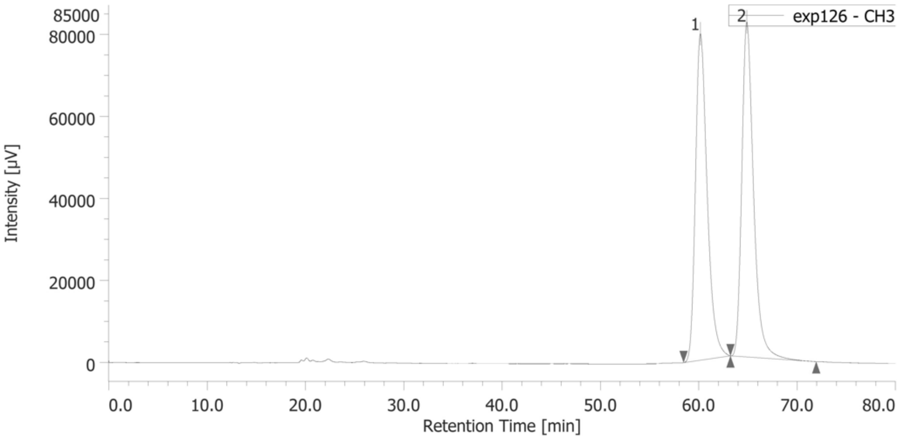

(*S,E*)-6-Fluoro-1-(1,3-diphenylallyl)indoline-2,3-dione ((*S*)-13g)

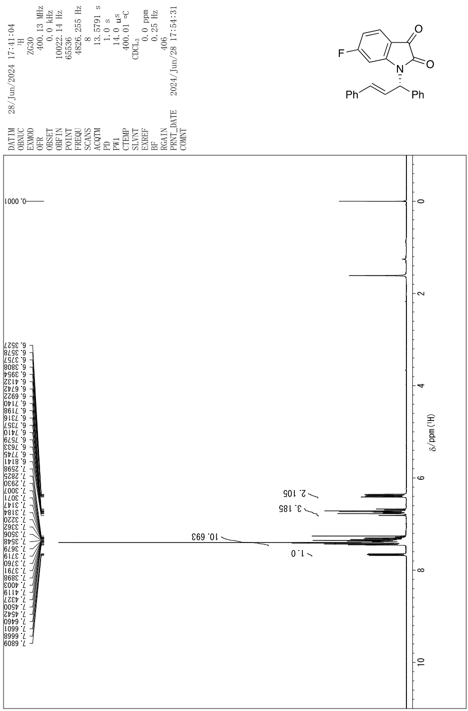

S48

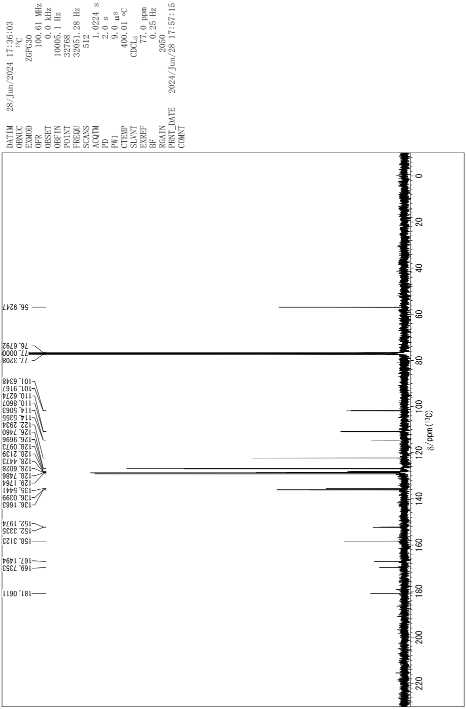
DATIM 28/Jun/2024 17:36:03
OBNUC 13C
EXMOD ZGPG30
OFR 100.61 MHz
OBSET 0.0 kHz
OBFIN 10005.1 Hz
POINT 32768
FREQU 32051.28 Hz
SCANS 512
ACQTM 1.0224 s
PD 2.0 s
PW1 9.0 us
CTEMP 400.01 °C
SLVNT CDCl3
EXREF 77.0 ppm
BF 0.25 Hz
RGAIN 2050
PRNT_DATE 2024/Jun/28 17:57:15
COMNT
56.9247
76.6792
77.0000
77.3208
101.6348
101.9167
110.6274
110.8607
114.5063
114.5355
122.2934
126.7460
126.9696
128.0973
128.2139
128.4473
128.6028
128.7486
129.1764
135.5441
136.0399
136.1663
152.1974
152.3335
158.3123
167.1494
169.7353
181.0611
δ/ppm(13C)
0
20
40
60
80
100
120
140
160
180
200
220

S49

Chromatogram

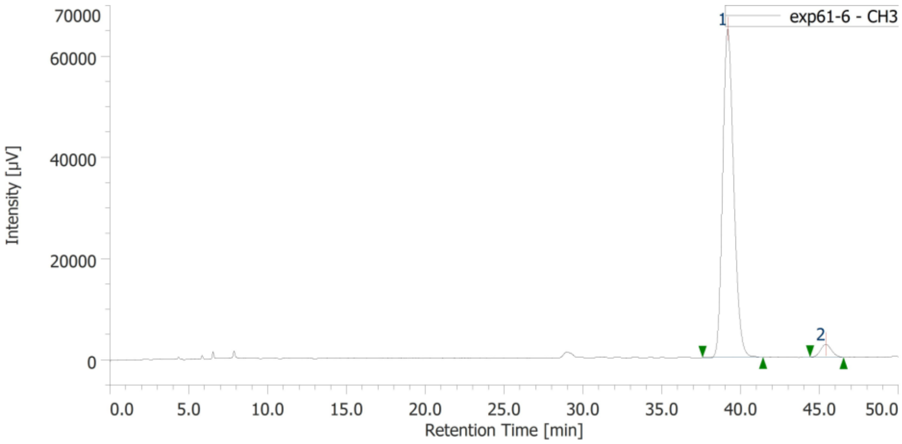

Chromatogram Information
User Name 3385
Date Modified 2023/07/07 21:06:45
Description AD−3, 0.7 mL/min, hex:2pro=95:5
HPLC System Name SYSTEM−3
Injection Date 2023/07/07 18:42:38
Volume 1.0 [µL]
Sample # 1
Project Name 2023system3
Acquisition Time 180.0 [min]
Acquisition Sequence 3−1499 nats
Control Method 2019system3
Peak ID Table
Calibration Method
Additional Information

Channel & Peak Information Table
Chromatogram Name exp61−6−CH3
Sample Name
Channel Name UV−2075
Sampling Interval 500 [msec]
Peak Method (Manual)

#	Peak Name	CH	tR [min]	Area [µV·sec]	Height [µV]	Area%	Height%
1	Unknown	3	39.16	3139408	64832	96.179	96.212
2	Unknown	3	45.38	124720	2553	3.821	3.788

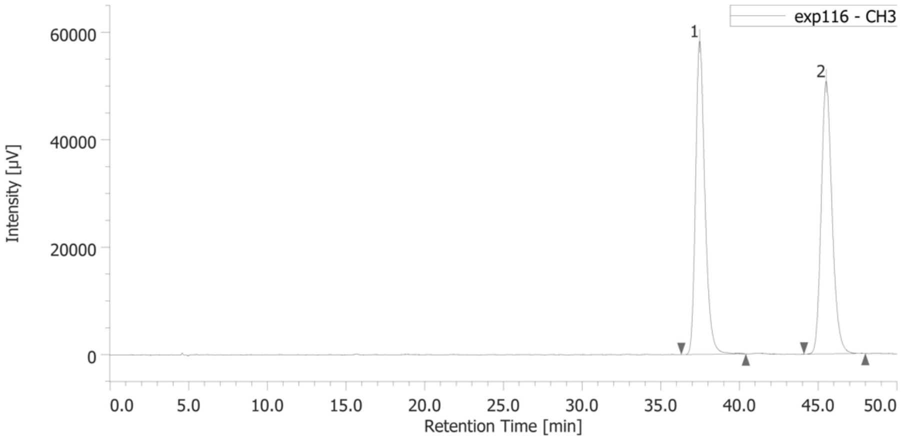

(*S*,*E*)-6-Chloro-1-(1,3-diphenylallyl)indoline-2,3-dione ((*S*)-13h)

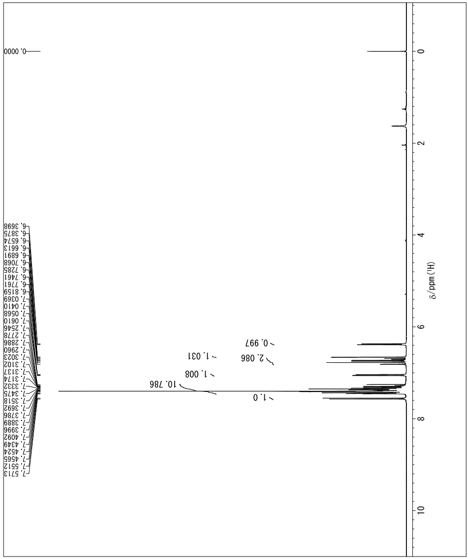

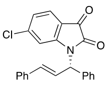

S51

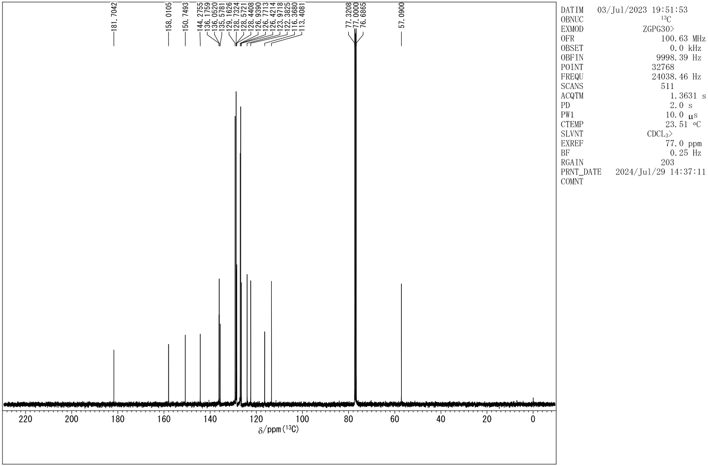

181.7042
158.0105
150.7493
144.2755
136.1759
136.0520
135.5781
129.1626
128.7324
128.5721
128.4408
126.9390
126.7713
126.4214
123.9718
122.3825
116.3680
113.4081
77.3208
77.0000
76.6865
57.0900
220
200
180
160
140
120
100
80
60
40
20
0
δ/ppm(13C)
DATIM 03/Jul/2023 19:51:53
OBNUC 13C
EXMOD ZGPG30>
OFR 100.63 MHz
OBSET 0.0 kHz
OBFIN 9998.39 Hz
POINT 32768
FREQU 24038.46 Hz
SCANS 511
ACQTM 1.3631 s
PD 2.0 s
PW1 10.0 μs
CTEMP 23.51 °C
SLVNT CDCL3>
EXREF 77.0 ppm
BF 0.25 Hz
RGAIN 203
PRNT_DATE 2024/Jul/29 14:37:11
COMNT

S52

Chromatogram

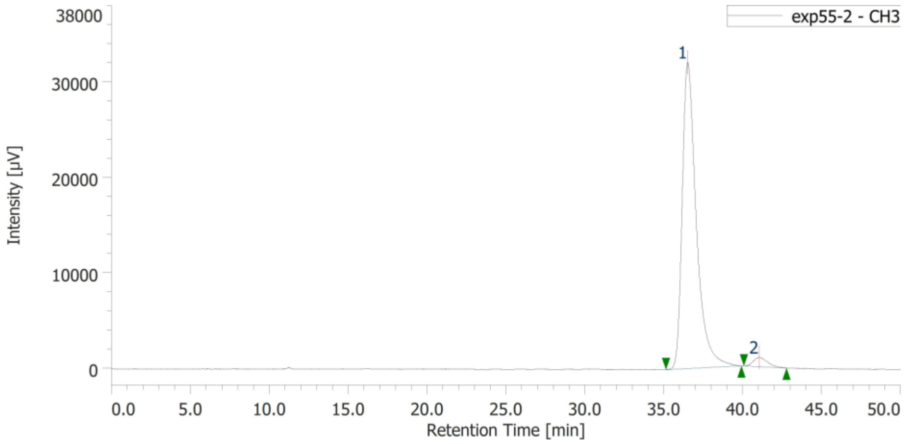

Chromatogram Information
User Name 3385
Date Modified 2023/05/28 19:05:17
Description IA3, 0.5 mL/min, hex:2pro=95:5
HPLC System Name SYSTEM-3
Injection Date 2023/05/28 14:33:56
Volume 1.0 [µL]
Sample # 1
Project Name 2023system3
Acquisition Time 180.0 [min]
Acquisition Sequence 3-1418 nats
Control Method 2019system3
Peak ID Table
Calibration Method
Additional Information

Channel & Peak Information Table
Chromatogram Name exp55-2-CH3
Sample Name
Channel Name UV-2075
Sampling Interval 500 [msec]
Peak Method (Manual)

#	Peak Name	CH	tR [min]	Area [µV·sec]	Height [µV]	Area%	Height%
1	Unknown	3	36.51	1986226	32064	97.065	97.107
2	Unknown	3	41.03	60069	955	2.935	2.893

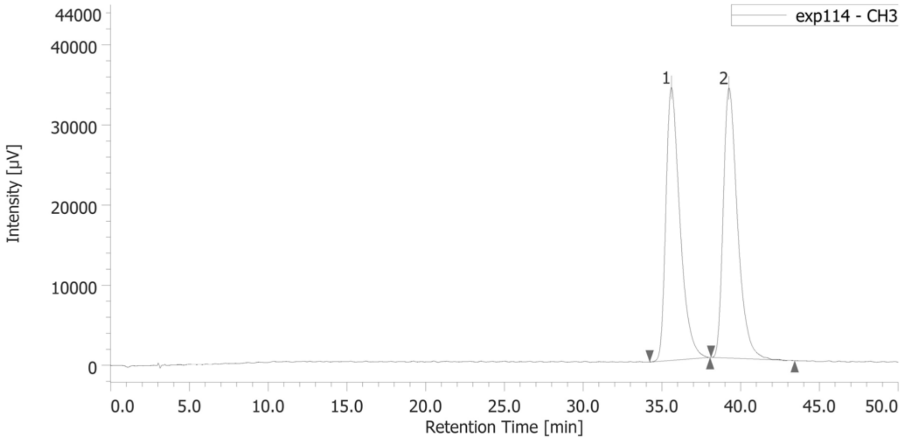

(*S,E*)-6-Bromo-1-(1,3-diphenylallyl)indoline-2,3-dione ((*S*)-13i)

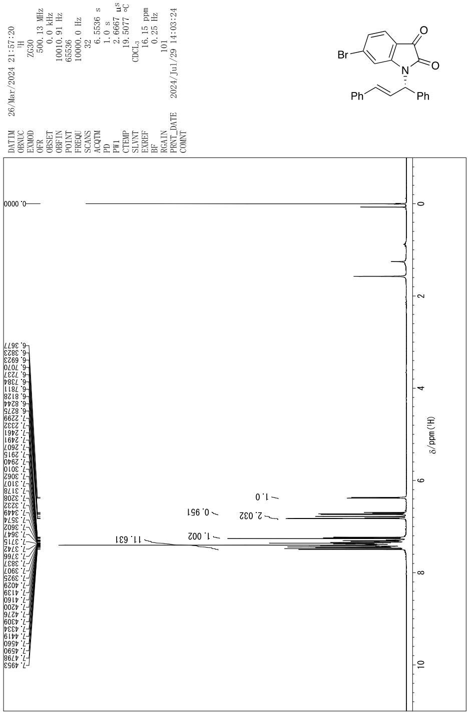

S54

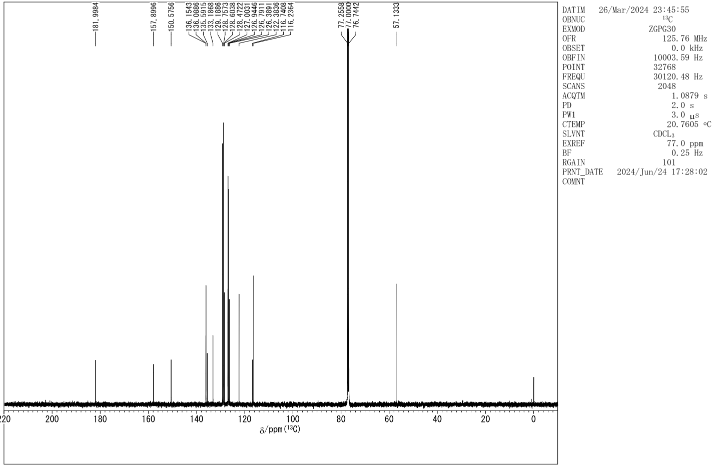
181.9984
157.8996
150.5756
136.1543
136.0886
135.5915
133.1868
129.1886
128.7573
128.6038
128.4722
127.0031
126.9446
126.7911
126.3891
122.3836
116.7408
116.2364
77.2558
77.0000
76.7442
57.1333
δ/ppm(13C)
DATIM 26/Mar/2024 23:45:55
OBNUC 13C
EXMOD ZGPG30
OFR 125.76 MHz
OBSET 0.0 kHz
OBFIN 10003.59 Hz
POINT 32768
FREQU 30120.48 Hz
SCANS 2048
ACQTM 1.0879 s
PD 2.0 s
PW1 3.0 us
CTEMP 20.7605 °C
SLVNT CDCL3
EXREF 77.0 ppm
BF 0.25 Hz
RGAIN 101
PRNT_DATE 2024/Jun/24 17:28:02
COMNT

S55

Chromatogram

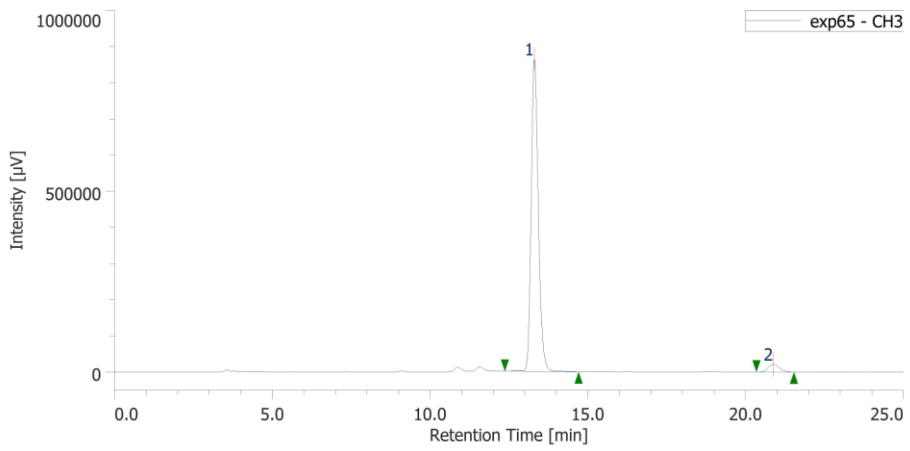

Chromatogram Information	
User Name	3385
Date Modified	2024/06/14 15:52:37
Description	AD-3, 1.0 mL/min, hex:2-PrOH=85:15, UV = 254 nm, CD =270 nm
HPLC System Name	SYSTEM-3
Injection Date	2024/06/14 13:11:04
Volume	1.0 [µL]
Sample #	1
Project Name	2024system3
Acquisition Time	180.0 [min]
Acquisition Sequence	3-1983 nats
Control Method	2019system3
Peak ID Table	
Calibration Method	
Additional Information	

Channel & Peak Information Table	
Chromatogram Name	exp65-CH3
Sample Name	
Channel Name	UV-2075
Sampling Interval	500 [msec]
Peak Method	(Manual)

#	Peak Name	CH	tR [min]	Area [µV·sec]	Height [µV]	Area%	Height%
1	Unknown	3	13.30	13606735	862801	96.29	97.6
2	Unknown	3	20.88	523822	21422	3.707	2.42

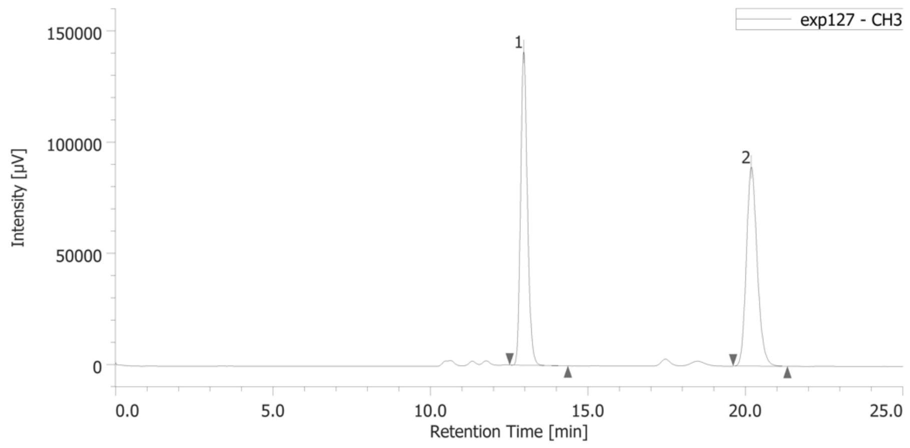

(*S,E*)-6-Methoxy-1-(1,3-diphenylallyl)indoline-2,3-dione ((*S*)-13j)

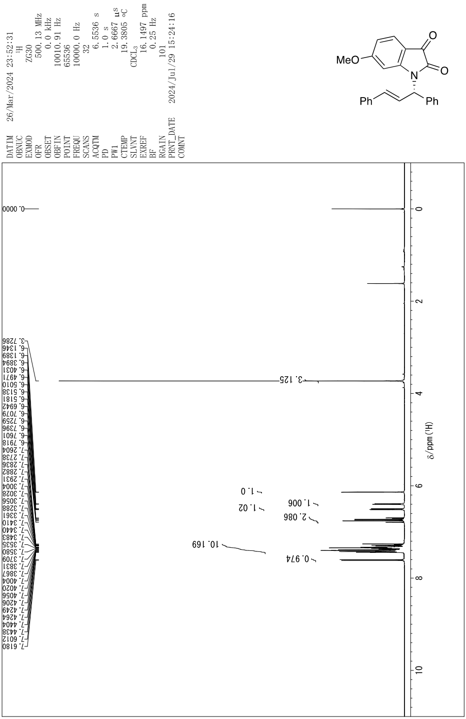

S57

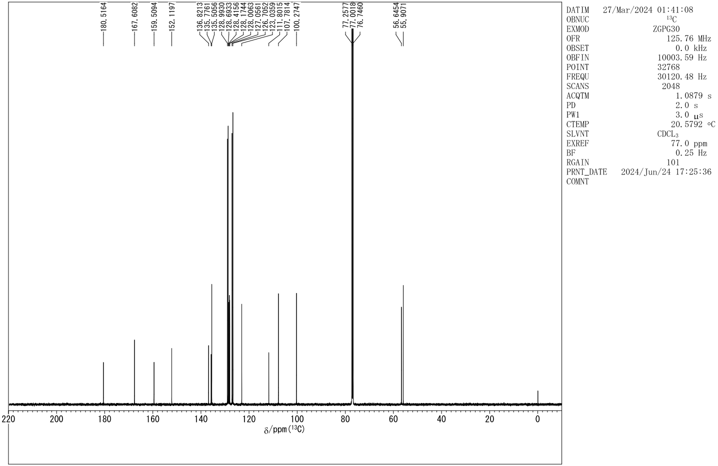
180.5164
167.6082
159.5094
152.1197
136.8213
135.7761
135.5056
128.9930
128.6933
128.4156
128.1744
128.0063
127.0561
126.7052
123.0359
111.8015
107.7814
100.2747
77.2577
77.0018
76.7460
56.6454
55.9071
220
200
180
160
140
120
100
80
60
40
20
0
δ/ppm(13C)
DATIM 27/Mar/2024 01:41:08
OBNUC 13C
EXMOD ZGPG30
OFR 125.76 MHz
OBSET 0.0 kHz
OBFIN 10003.59 Hz
POINT 32768
FREQU 30120.48 Hz
SCANS 2048
ACQTM 1.0879 s
PD 2.0 s
PW1 3.0 us
CTEMP 20.5792 °C
SLVNT CDCL3
EXREF 77.0 ppm
BF 0.25 Hz
RGAIN 101
PRNT_DATE 2024/Jun/24 17:25:36
COMNT

S58

Chromatogram

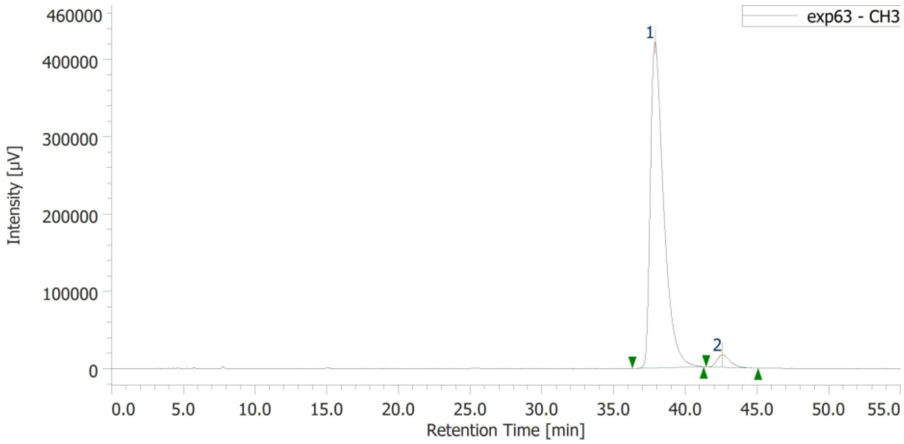

Chromatogram Information

User Name	3385
Date Modified	2024/07/02 16:00:44
Description	IA-3, 1.0 mL/min, hex:2-PrOH=95:5, UV = 254 nm, CD =270 nm
HPLC System Name	SYSTEM-3
Injection Date	2024/06/13 8:50:23
Volume	1.0 [µL]
Sample #	1
Project Name	2024system3
Acquisition Time	180.0 [min]
Acquisition Sequence	3-1975 nats
Control Method	2019system3
Peak ID Table	
Calibration Method	
Additional Information	

Channel & Peak Information Table

Chromatogram Name	exp63-CH3
Sample Name	
Channel Name	UV-2075
Sampling Interval	500 [msec]
Peak Method	(Manual)

#	Peak Name	CH	tR [min]	Area [µV·sec]	Height [µV]	Area%	Height%
1	Unknown	3	37.88	27200278	421773	96.33	96.3
2	Unknown	3	42.58	1035738	16284	3.668	3.72

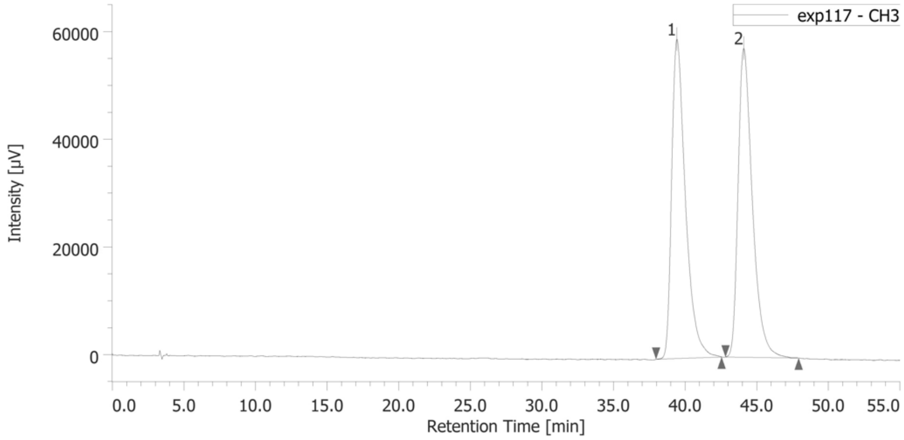

(*S,E*)-7-Chloro-1-(1,3-diphenylallyl)indoline-2,3-dione ((*S*)-13k)

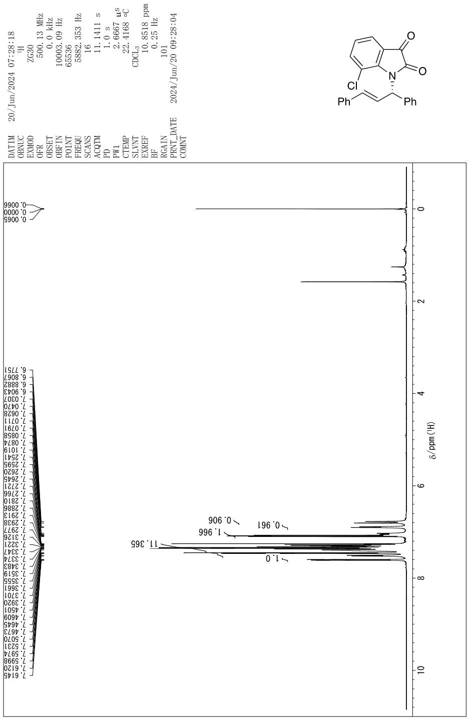

S60

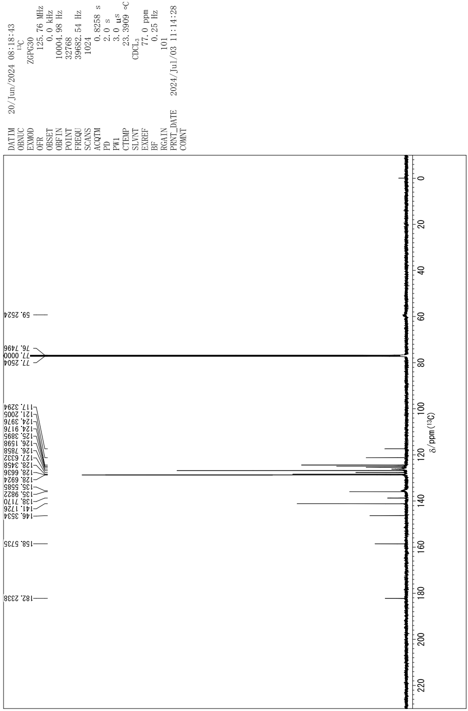
DATIM 20/Jun/2024 08:18:43
OBNUC 13C
EXMOD ZGPG30
OFR 125.76 MHz
OBSET 0.0 kHz
OBFIN 10004.98 Hz
POINT 32768
FREQU 39682.54 Hz
SCANS 1024
ACQTM 0.8258 s
PD 2.0 s
PW1 3.0 us
CTEMP 23.3909 °C
SLVNT CDCL3
EXREF 77.0 ppm
BF 0.25 Hz
RGAIN 101
PRNT_DATE 2024/Jul/03 11:14:28
COMNT
59.2524
76.7496
77.0000
77.2504
117.3294
121.2005
124.3976
124.9176
125.3895
126.1598
126.7858
127.6332
128.3458
128.6636
128.6924
135.5585
135.9822
138.7170
141.1726
146.3534
158.5735
182.2338
0
20
40
60
80
100
120
140
160
180
200
220
δ/ppm(13C)

S61

Chromatogram

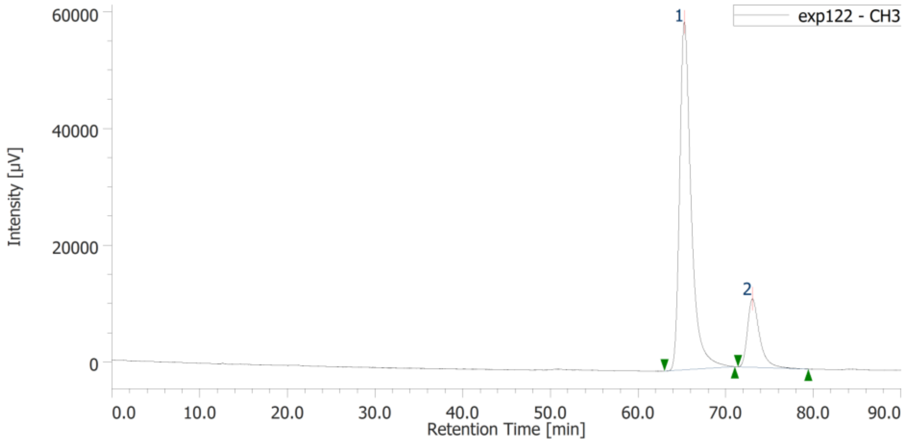

Chromatogram Information	
User Name	3385
Date Modified	2024/06/18 16:46:40
Description	IA-IA3,0.5 mL/min, Hex:2-PrOH=93:7,UV254nm,CD270nm
HPLC System Name	HPLC-1
Injection Date	2024/06/18 14:06:44
Volume	1.00 [µL]
Sample #	1
Project Name	2024system1
Acquisition Time	180.0 [min]
Acquisition Sequence	1-2719 nats
Control Method	system1_2017
Peak ID Table	
Calibration Method	
Additional Information	

Channel & Peak Information Table	
Chromatogram Name	exp122-CH3
Sample Name	
Channel Name	UV-2075
Sampling Interval	500 [msec]
Peak Method	(Manual)

#	Peak Name	CH	tR [min]	Area [µV·sec]	Hight [µV]	Area%	Hight%
1	Unknown	3	65.267	5484742	59561	83.08	83.6
2	Unknown	3	73.042	1117261	11722	16.92	16.4

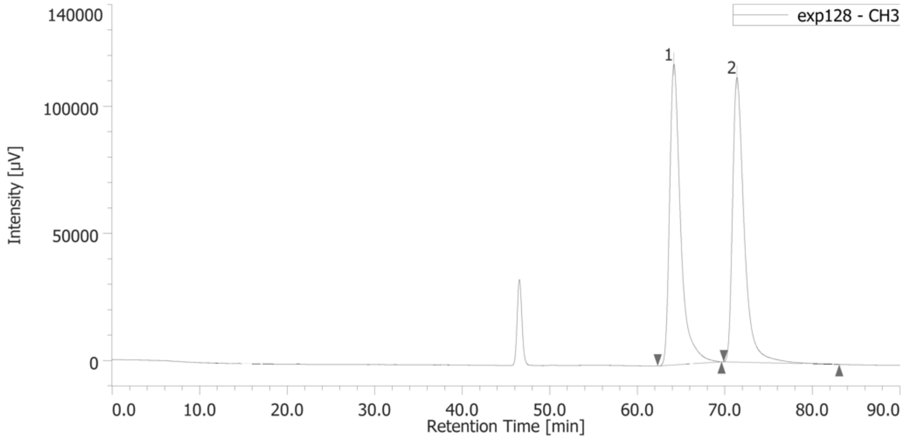

(*S,E*)-1-(1,3-Bis(4-chlorophenyl)allyl)indoline-2,3-dione ((*S*)-13l)

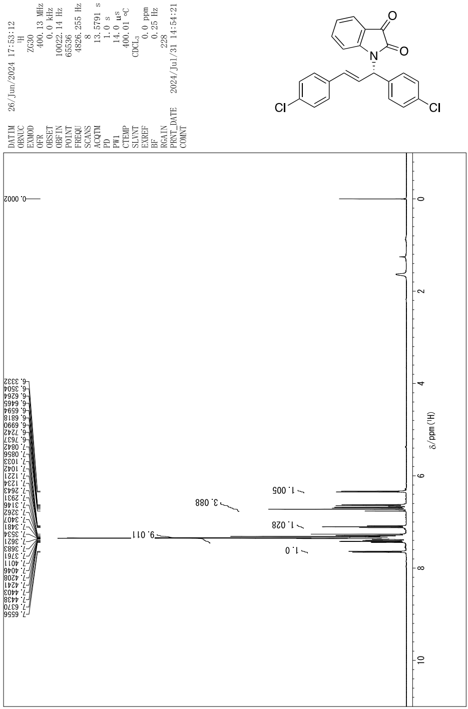

S63

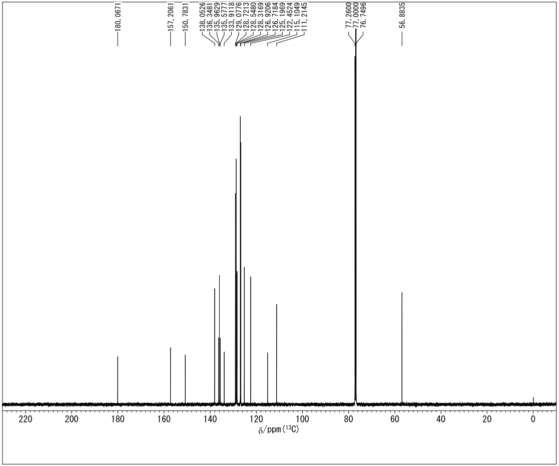

DATIM	15/Dec/2024 21:35:20
OBNUC	^{13}C
EXMOD	ZGPG30
OFR	125.76 MHz
OBSET	0.0 kHz
OBFIN	10004.98 Hz
POINT	32768
FREQU	39682.54 Hz
SCANS	512
ACQTM	0.8258 s
PD	2.0 s
PW1	3.0 μs
CTEMP	20.2718 °C
SLVNT	$CDCL_3$
EXREF	77.0 ppm
BF	0.25 Hz
RGAIN	101
PRNT_DATE	2024/Dec/16 10:35:43
COMNT	

S64

Chromatogram

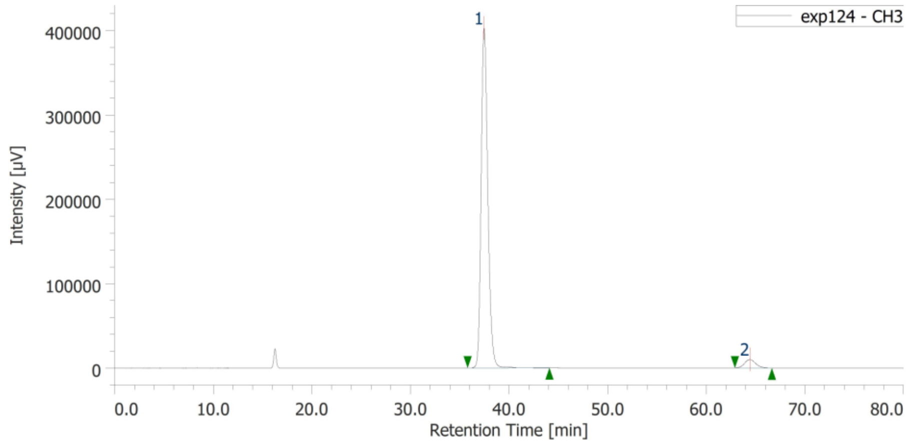

Chromatogram Information

User Name	3385
Date Modified	2024/06/17 11:17:04
Description	AD3, 1.0 mL/min, hex:2−PrOH=90:10, UV = 254 nm, CD =270 nm
HPLC System Name	SYSTEM−3
Injection Date	2024/06/17 7:30:52
Volume	1.0 [µL]
Sample #	1
Project Name	2024system3
Acquisition Time	180.0 [min]
Acquisition Sequence	3−1992 nats
Control Method	2019system3
Peak ID Table	
Calibration Method	
Additional Information	

Channel & Peak Information Table

Chromatogram Name	exp124−CH3
Sample Name	
Channel Name	UV−2075
Sampling Interval	500 [msec]
Peak Method	(Manual)

#	Peak Name	CH	tR [min]	Area [µV·sec]	Height [µV]	Area%	Height%
1	Unknown	3	37.46	18853681	402498	96.05	97.6
2	Unknown	3	64.41	775888	10004	3.953	2.43

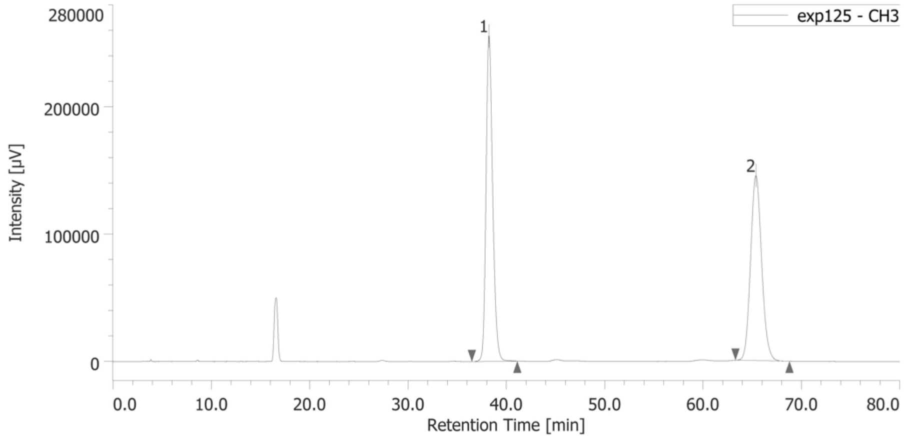

(*S,E*)-2-(1,3-Diphenylallyl-2-oxoindolin-3-ylidene)malononitrile ((*S*)-16)

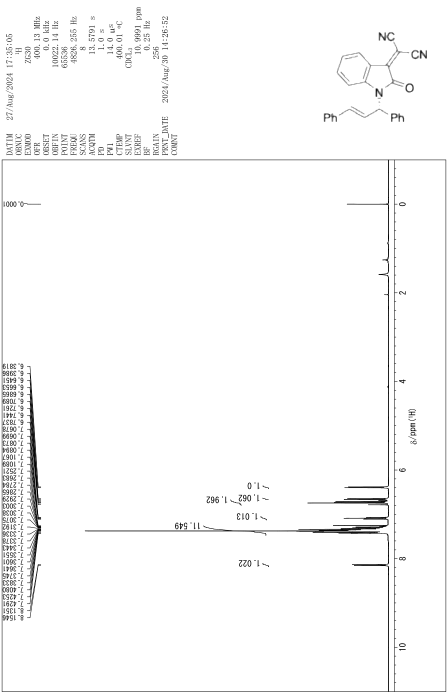

S66

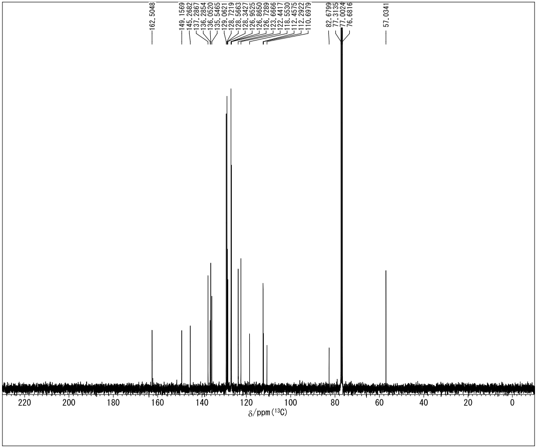

DATIM 27/Aug/2024 17:51:33
OBNUC ^{13}C
EXMOD ZGPG30
OFR 100.61 MHz
OBSET 0.0 kHz
OBFIN 10005.1 Hz
POINT 32768
FREQU 32051.28 Hz
SCANS 256
ACQTM 1.0224 s
PD 2.0 s
PW1 9.0 μs
CTEMP 400.01 °C
SLVNT $CDCl_3$
EXREF 77.0 ppm
BF 0.25 Hz
RGAIN 2050
PRNT_DATE 2024/Aug/30 14:29:09
COMNT

S67

Chromatogram

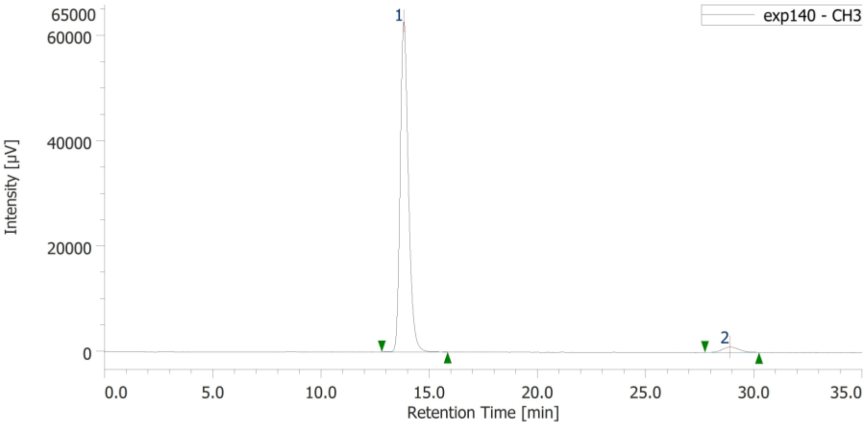

Chromatogram Information

User Name	3385
Date Modified	2024/08/29 14:22:41
Description	AD-H, 1.5 mL/min, hex:EtOH=90:10
HPLC System Name	SYSTEM-3
Injection Date	2024/08/29 13:42:58
Volume	1.0 [μL]
Sample #	1
Project Name	2024system3
Acquisition Time	180.0 [min]
Acquisition Sequence	3-2118 nats
Control Method	2019system3
Peak ID Table	
Calibration Method	
Additional Information	

Channel & Peak Information Table

Chromatogram Name	exp140-CH3
Sample Name	
Channel Name	UV-2075
Sampling Interval	500 [msec]
Peak Method	(Manual)

#	Peak Name	CH	tR [min]	Area [μV·sec]	Height [μV]	Area%	Height%
1	Unknown	3	13.82	1656223	62786	96.90	98.4
2	Unknown	3	28.88	52964	1010	3.099	1.58

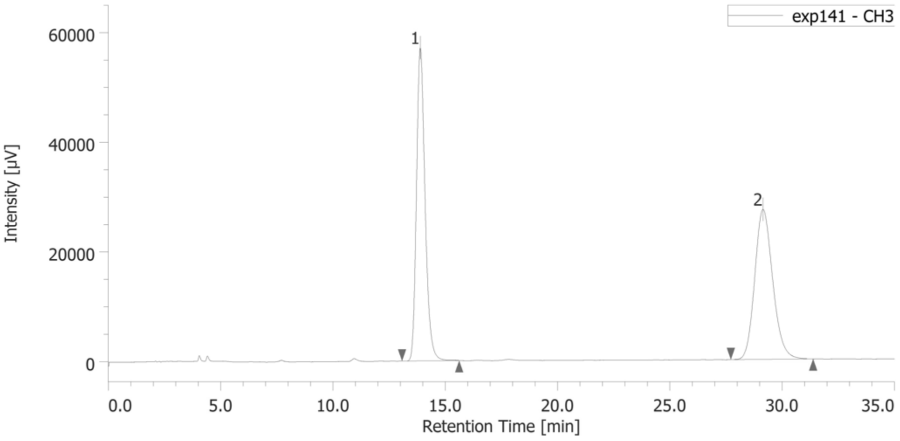

Cartesian coordinates and energies

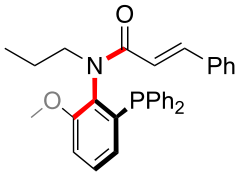

0°

at wb97xd/6-31+G*
Energy = -1745.454486 A.U.
Thermal correction to Gibbs Free Energy = 0.471179 A.U.
Sum of electronic and thermal Free Energies = -1744.983307 A.U.

P	-2.34802700	-0.02042300	-0.82269300
C	2.29029300	-0.77916400	-0.03924100
C	-1.85360700	-0.82664100	0.78116900
H	1.74149100	0.06103600	0.37675100
C	3.60125000	-0.71050000	-0.29466700
C	-0.60396300	-1.50422300	0.81253900
C	-2.53552900	-0.57075900	1.96907600
N	0.08298400	-1.68126400	-0.43162200
C	0.03580600	-1.59063700	2.06049800
C	-1.94081000	-0.81904200	3.20530800
H	-3.50699600	-0.08772500	1.93839000
C	-0.63863700	-1.28894700	3.24350800
H	-2.47162500	-0.59004900	4.12454700
H	-0.11120200	-1.43132100	4.18224800
C	-0.99607200	1.24020200	-0.84337000
C	-0.39830100	1.53944500	-2.06998300
C	-0.48459700	1.83828500	0.31643400
C	0.69553100	2.40185200	-2.14044900
H	-0.77507500	1.07536900	-2.97806900
C	0.60136800	2.70569400	0.24720800
H	-0.92493600	1.60990600	1.28400600
C	1.20220400	2.98118000	-0.98100100
H	1.16118900	2.60589500	-3.10034100
H	0.98938900	3.15760100	1.15645600
H	2.06869100	3.63444900	-1.02918800
C	-3.83778500	0.93133200	-0.31305200
C	-5.05747400	0.24046500	-0.33010600
C	-3.83983600	2.28379000	0.04181600
C	-6.24320600	0.87395100	0.03016300
H	-5.07835100	-0.80642400	-0.62666500
C	-5.02962200	2.92436700	0.38573500
H	-2.91131800	2.84644300	0.04714900
C	-6.23169400	2.22131800	0.38857400
H	-7.17727400	0.31915600	0.01940100
H	-5.01379900	3.97707600	0.65443400
H	-7.15675000	2.72184900	0.66043900
C	1.48428400	-1.95944400	-0.42503700
O	1.95102200	-2.98619300	-0.88172200
H	4.07226000	-1.57051300	-0.76950900
C	4.46079100	0.45191800	-0.02809700
C	5.69744900	0.55280300	-0.67842700
C	4.08649400	1.47642400	0.85421400
C	6.52583100	1.65372600	-0.47697800
H	6.00650800	-0.23862900	-1.35727700
C	4.91297700	2.57479000	1.05836100
H	3.14751900	1.40492600	1.39486200
C	6.13442600	2.67104200	0.39019200
H	7.47818200	1.71533800	-0.99602700
H	4.60886000	3.35622600	1.74967900
H	6.78007100	3.52933400	0.55327300
C	-0.61996900	-2.66311700	-1.31336700
C	-0.39575700	-2.37490900	-2.79212000
H	-0.27970800	-3.67647200	-1.06950600
H	-0.72347900	-1.35118000	-3.00381700
H	0.67259600	-2.43420200	-3.02336400
O	1.31410300	-2.08248800	2.15183900
C	1.38984900	-3.48764800	2.36370700
H	2.45166100	-3.73579400	2.38548300
H	0.91794200	-3.76225200	3.31566600
H	0.90713300	-4.02931200	1.54107100
H	-1.68613900	-2.60978000	-1.09120500
C	-1.17087100	-3.35905300	-3.66615300
H	-0.85734900	-4.39187600	-3.47292300
H	-2.24878900	-3.29115200	-3.47464300
H	-1.00713100	-3.15428500	-4.72921500

1°

at wb97xd/6-31+G*
Energy = -1745.453741 A.U.
Thermal correction to Gibbs Free Energy = 0.471911 A.U.
Sum of electronic and thermal Free Energies = -1744.98183 A.U.

P	-2.41200500	0.04993100	0.75706600
C	2.38803600	0.68511600	0.16690500
C	-1.77465600	0.91559800	-0.76032000
H	1.84409600	-0.13690900	-0.29057200
C	3.70885700	0.64259500	0.37467200
C	-0.49362500	1.53101500	-0.69725100
C	-2.42287200	0.78734900	-1.98803800
N	0.16985700	1.58030900	0.57037200
C	0.18385000	1.70764100	-1.91680900
C	-1.77830500	1.10747400	-3.18129000
H	-3.41668600	0.35350200	-2.02693900
C	-0.46052600	1.53054700	-3.14034600
H	-2.28680700	0.97524700	-4.13154000
H	0.10217600	1.73229100	-4.04717800
C	-1.19166200	-1.33594100	0.78431200
C	-0.86497900	-1.88719600	2.02650200
C	-0.56045600	-1.83807500	-0.36088900
C	0.06929200	-2.91706000	2.12767100

H	-1.33682300	-1.49952600	2.92640200
C	0.37181800	-2.86725900	-0.26264800
H	-0.79274700	-1.41957900	-1.33691500
C	0.69183000	-3.40717400	0.98286900
H	0.31809400	-3.32718700	3.10226200
H	0.85173300	-3.24598600	-1.16163400
H	1.42757800	-4.20305900	1.06017400
C	-3.94022800	-0.75064000	0.11613800
C	-5.12233500	-0.00361900	0.20672100
C	-4.00206900	-2.04115900	-0.41939900
C	-6.33075600	-0.51950400	-0.25366700
H	-5.09564100	0.99359600	0.64129500
C	-5.21423200	-2.56555400	-0.86503000
H	-3.10199300	-2.64474600	-0.48868300
C	-6.37906300	-1.80515900	-0.79007200
H	-7.23570000	0.07745300	-0.18260400
H	-5.24607500	-3.57157300	-1.27433100
H	-7.32201600	-2.21499300	-1.14093500
C	1.57718400	1.83982800	0.61509400
O	2.04200200	2.82877500	1.15006400
H	4.17453600	1.51832200	0.82557200
C	4.60079500	-0.47695800	0.04195200
C	5.98496000	-0.26274100	0.01743900
C	4.11970100	-1.76283300	-0.24689200
C	6.86401600	-1.29201900	-0.30897200
H	6.37379900	0.72565000	0.25111500
C	4.99611300	-2.79168100	-0.56989700
H	3.05350600	-1.96358300	-0.19737600
C	6.37191900	-2.56050500	-0.60654300
H	7.93367200	-1.10326300	-0.32777500
H	4.60597000	-3.78248800	-0.78696400
H	7.05492300	-3.36715800	-0.85734000
C	-0.52999400	2.48551900	1.53313800
C	-0.34976400	2.03037300	2.97548300
H	-0.15375400	3.50712400	1.40738300
H	-0.68341000	0.98993100	3.06065300
H	0.71235200	2.05605400	3.24082500
O	1.47668800	2.16825500	-1.92950700
C	1.59612700	3.58177900	-2.03837000
H	2.66441800	3.79998300	-2.01020800
H	1.16345100	3.93667600	-2.98236800
H	1.10171200	4.07776400	-1.19414500
H	-1.59055300	2.49060900	1.28300000
C	-1.14607000	2.91317200	3.93424400
H	-0.82811500	3.96032300	3.86497900
H	-2.21864700	2.86939700	3.70880000
H	-1.00893600	2.59121000	4.97176000

2°

at wb97xd/6-31+G*
Energy = -1745.452956 A.U.
Thermal correction to Gibbs Free Energy = 0.471726 A.U.
Sum of electronic and thermal Free Energies = -1744.98123 A.U.

P	-2.41568800	0.04815900	0.76155000
C	2.39442300	0.68558900	0.16667000
C	-1.78145700	0.92843200	-0.74913200
H	1.85164700	-0.13617100	-0.29258100
C	3.71524300	0.64430700	0.37482400
C	-0.49670800	1.53725200	-0.68712800
C	-2.43663300	0.81459700	-1.97457900
N	0.17373500	1.57093600	0.57723700
C	0.17634200	1.71550600	-1.90901300
C	-1.79629600	1.14020800	-3.16860100
H	-3.43311800	0.38686600	-2.01209300
C	-0.47514100	1.55265200	-3.13075200
H	-2.31051600	1.01909600	-4.11723600
H	0.08499200	1.75619900	-4.03878300
C	-1.19628200	-1.33890000	0.77240700
C	-0.87188000	-1.90611000	2.00800600
C	-0.56368300	-1.82697100	-0.37807400
C	0.06161000	-2.93773500	2.09761400
H	-1.34492700	-1.52959100	2.91202200
C	0.36824500	-2.85752100	-0.29134700
H	-0.79434600	-1.39628300	-1.34917200
C	0.68597400	-3.41340400	0.94770700
H	0.30844500	-3.36047000	3.06731100
H	0.84957600	-3.22488800	-1.19427900
H	1.42117300	-4.21062600	1.01608400
C	-3.94580000	-0.74579700	0.11687300
C	-5.12887400	-0.00283400	0.22565900
C	-4.00783000	-2.02769000	-0.43906900
C	-6.33873700	-0.51385800	-0.23651900
H	-5.10183800	0.98730600	0.67608000
C	-5.22122200	-2.54741400	-0.88664200
H	-3.10690900	-2.62816500	-0.52294500
C	-6.38723200	-1.79078700	-0.79323700
H	-7.24452000	0.07992100	-0.15102600
H	-5.25322300	-3.54677300	-1.31192900
H	-7.33118700	-2.19699000	-1.14562700
C	1.58115200	1.83745500	0.61736900
O	2.04377900	2.82796100	1.15090200
H	4.17991200	1.51953400	0.82774900
C	4.60824200	-0.47380200	0.04006700
C	5.99215000	-0.25801500	0.01485000
C	4.12831400	-1.75986800	-0.24997800
C	6.87210500	-1.28588900	-0.31353700
H	6.38006600	0.73050200	0.24954000
C	5.00566100	-2.78730200	-0.57495900
H	3.06236700	-1.96196100	-0.19978800
C	6.38117700	-2.55452600	-0.61237600
H	7.94153900	-1.09593100	-0.33287300
H	4.61648900	-3.77829300	-0.79289400
H	7.06488400	-3.36010800	-0.86469100
C	-0.52195200	2.46378000	1.55508100
C	-0.33136300	1.99084100	2.99030100
H	-0.14801400	3.48729200	1.43929500
H	-0.66245000	0.94874600	3.06460200
H	0.73255700	2.01486400	3.24887100
O	1.47320500	2.16479700	-1.92436600
C	1.60576300	3.57689000	-2.03672100

H	2.67609000	3.78508800	-2.00964800
H	1.17606300	3.93367500	-2.98132900
H	1.11637200	4.07927900	-1.19338200
H	-1.58402100	2.47080300	1.31222800
C	-1.12245800	2.86007700	3.96559100
H	-0.80671800	3.90856500	3.90733800
H	-2.19644800	2.81725100	3.74682800
H	-0.97773000	2.52528100	4.99800600

3°

at wb97xd/6-31+G*
Energy = -1745.452162 A.U.
Thermal correction to Gibbs Free Energy = 0.471682 A.U.
Sum of electronic and thermal Free Energies = -1744.98048 A.U.

P	-2.41936000	0.04582500	0.76637300
C	2.40106100	0.68608600	0.16663400
C	-1.78855400	0.94233100	-0.73654800
H	1.85957200	-0.13509600	-0.29509800
C	3.72185800	0.64590300	0.37527900
C	-0.50000000	1.54430200	-0.67549900
C	-2.45110200	0.84462600	-1.95942600
N	0.17785200	1.56107700	0.58508800
C	0.16812000	1.72564000	-1.89976500
C	-1.81547500	1.17728200	-3.15399300
H	-3.45029300	0.42318900	-1.99559200
C	-0.49094000	1.57879900	-3.11944500
H	-2.33579800	1.06866800	-4.10079500
H	0.06618600	1.78515300	-4.02869900
C	-1.20090800	-1.34223700	0.75916000
C	-0.87921500	-1.92724600	1.98715700
C	-0.56664900	-1.81445500	-0.39699500
C	0.05330600	-2.96076600	2.06381500
H	-1.35370300	-1.56329000	2.89557500
C	0.36474500	-2.84651800	-0.32316300
H	-0.79539400	-1.36991900	-1.36231000
C	0.67971700	-3.42025600	0.90843300
H	0.29786300	-3.39758700	3.02783800
H	0.84773900	-3.20112600	-1.23029100
H	1.41420000	-4.21895000	0.96682200
C	-3.95136600	-0.74093800	0.11730700
C	-5.13549200	-0.00279700	0.24520200
C	-4.01343200	-2.01327400	-0.46026900
C	-6.34673100	-0.50879600	-0.21903900
H	-5.10823200	0.97951700	0.71241400
C	-5.22798600	-2.52816700	-0.91008800
H	-3.11156000	-2.60998000	-0.55945000
C	-6.39526500	-1.77607600	-0.79728500
H	-7.25343600	0.08118600	-0.11839100
H	-5.26001400	-3.52014700	-1.35231600
H	-7.34018600	-2.17850200	-1.15141800
C	1.58528900	1.83475500	0.62059800
O	2.04565700	2.82664600	1.15303200
H	4.18557900	1.52027800	0.83082800
C	4.61577500	-0.47069900	0.03783100
C	5.99936100	-0.25317700	0.01022400
C	4.13681400	-1.75711500	-0.25234200
C	6.87993500	-1.27966100	-0.32081500
H	6.38651500	0.73559700	0.24510200
C	5.01483500	-2.78315800	-0.57996300
H	3.07120600	-1.96067200	-0.20005700
C	6.38997400	-2.54862800	-0.61987300
H	7.94909800	-1.08838500	-0.34199600
H	4.62648800	-3.77447000	-0.79788700
H	7.07418700	-3.35315100	-0.87418100
C	-0.51319800	2.43987600	1.57933200
C	-0.31140400	1.94736200	3.00637800
H	-0.14182700	3.46542600	1.47477700
H	-0.63993300	0.90365700	3.06861500
H	0.75443200	1.96950500	3.25754200
O	1.46907000	2.16299600	-1.91777300
C	1.61552500	3.57348800	-2.03274100
H	2.68787000	3.77112600	-2.00582600
H	1.18970800	3.93291400	-2.97808000
H	1.13083900	4.08202600	-1.19040100
H	-1.57686100	2.44883900	1.34440900
C	-1.09657400	2.80155800	3.99955100
H	-0.78294400	3.85128800	3.95357100
H	-2.17204500	2.75995000	3.78799500
H	-0.94376700	2.45263800	5.02611300

4°

at wb97xd/6-31+G*
Energy = -1745.451363 A.U.
Thermal correction to Gibbs Free Energy = 0.47172 A.U.
Sum of electronic and thermal Free Energies = -1744.979643 A.U.

P	2.42321600	-0.04322000	0.77145000
C	-2.40781400	-0.68643400	0.16673900
C	1.79572000	-0.95676100	-0.72297000
H	-1.86747400	0.13407000	-0.29745600
C	-3.72864400	-0.64735600	0.37553800
C	0.50335800	-1.55172500	-0.66283900
C	2.46585000	-0.87612200	-1.94297300
N	-0.18214600	-1.55074600	0.59353000
C	-0.15949300	-1.73710200	-1.88949700
C	1.83518100	-1.21670200	-3.13790200
H	3.46773900	-0.46107400	-1.97772300
C	0.50734000	-1.60722600	-3.10684700
H	2.36174800	-1.12134200	-4.08265900
H	-0.04656100	-1.81700200	-4.01729700
C	1.20587000	1.34576300	0.74539000
C	0.88744900	1.94951500	1.96515200
C	0.56956200	1.80120900	-0.41633200
C	-0.04393900	2.98494800	2.02833200
H	1.36365500	1.59884900	2.87791000
C	-0.36112200	2.83481800	-0.35590800
H	0.79590000	1.34208400	-1.37538500

C	-0.67279600	3.42734800	0.86759900
H	-0.28578400	3.43659800	2.98619400
H	-0.84602500	3.17597900	-1.26715800
H	-1.40642600	4.22753000	0.91560900
C	3.95705500	0.73596400	0.11751400
C	5.14238000	0.00370300	0.26583400
C	4.01888800	1.99748700	-0.48345100
C	6.35492000	0.50448000	-0.20085400
H	5.11509100	-0.96975900	0.75119800
C	5.23448800	2.50735000	-0.93594300
H	3.11589000	2.58955000	-0.59894200
C	6.40321800	1.76083200	-0.80238600
H	7.26268500	-0.08086100	-0.08398600
H	5.26633500	3.49096300	-1.39650300
H	7.34903100	2.15931900	-1.15858900
C	-1.58958000	-1.83170000	0.62443400
O	-2.04764900	-2.82485700	1.15600600
H	-4.19143600	-1.52079100	0.83384400
C	-4.62352500	0.46754600	0.03495600
C	-6.00683500	0.24843200	0.00624000
C	-4.14560600	1.75396800	-0.25702200
C	-6.88816300	1.27329900	-0.32776600
H	-6.39316300	-0.74031700	0.24259300
C	-5.02442500	2.77838800	-0.58760200
H	-3.08027700	1.95892200	-0.20374700
C	-6.39924900	2.54223200	-0.62868100
H	-7.95709100	1.08081200	-0.34977500
H	-4.63695900	3.76975000	-0.80683400
H	-7.08406100	3.34552300	-0.88525600
C	0.50394900	-2.41428200	1.60494000
C	0.29054200	-1.90098800	3.02290200
H	0.13527200	-3.44185200	1.51249400
H	0.61658800	-0.85579800	3.07208100
H	-0.77723100	-1.92109100	3.26633200
O	-1.46448800	-2.16226100	-1.91028100
C	-1.62508000	-3.57104100	-2.02729300
H	-2.69940300	-3.75781500	-2.00110100
H	-1.20268400	-3.93359300	-2.97293700
H	-1.14580700	-4.08537600	-1.18541300
H	1.56922500	-2.42506900	1.37832500
C	1.06932500	-2.73891400	4.03475900
H	0.75768400	-3.78973200	4.00206200
H	2.14628300	-2.69867100	3.83068300
H	0.90819400	-2.37491900	5.05479300

5°

at wb97xd/6-31+G*
Energy = -1745.450556 A.U.
Thermal correction to Gibbs Free Energy = 0.471818 A.U.
Sum of electronic and thermal Free Energies = -1744.978738 A.U.

P	2.42774100	-0.04069800	0.77636900
C	-2.41432200	-0.68626500	0.16728400
C	1.80291400	-0.97146100	-0.70893000
H	-1.87502600	0.13336600	-0.29956000
C	-3.73526500	-0.64829800	0.37575300
C	0.50677900	-1.55932900	-0.64938500
C	2.48035200	-0.90840900	-1.92596200
N	-0.18628500	-1.53979800	0.60250000
C	-0.15087000	-1.74945200	-1.87824700
C	1.85451800	-1.25761800	-3.12089700
H	3.48489400	-0.49978900	-1.95950900
C	0.52350600	-1.63723300	-3.09316900
H	2.38714900	-1.17602600	-4.06352600
H	-0.02723800	-1.85094000	-4.00462800
C	1.21121300	1.34871700	0.73222900
C	0.89576000	1.96996500	1.94397100
C	0.57278600	1.78815700	-0.43445600
C	-0.03490700	3.00671900	1.99448800
H	1.37375000	1.63202400	2.86061000
C	-0.35759000	2.82274900	-0.38661700
H	0.79691300	1.31570100	-1.38752300
C	-0.66634300	3.43273100	0.82901400
H	-0.27426700	3.47218400	2.94634800
H	-0.84441200	3.15105000	-1.30155400
H	-1.39946800	4.23391400	0.86727700
C	3.96291300	0.73113700	0.11679700
C	5.14961300	0.00556000	0.28498000
C	4.02394200	1.98127900	-0.50769700
C	6.36307400	0.50133300	-0.18487600
H	5.12276500	-0.95858200	0.78860200
C	5.24018000	2.48630800	-0.96361400
H	3.11964200	2.56805800	-0.63897600
C	6.41055100	1.74614500	-0.80988400
H	7.27204100	-0.07875000	-0.05221500
H	5.27140200	3.46107500	-1.44266300
H	7.35694000	2.14085100	-1.16876200
C	-1.59366200	-1.82791000	0.62922400
O	-2.04936400	-2.82209600	1.16046100
H	-4.19719300	-1.52059100	0.83710800
C	-4.63112500	0.46457800	0.03107500
C	-6.01417500	0.24389100	0.00195600
C	-4.15429900	1.75063500	-0.26443600
C	-6.89632900	1.26676300	-0.33594700
H	-6.39964100	-0.74452900	0.24108500
C	-5.03398300	2.77305400	-0.59890700
H	-3.08921700	1.95700600	-0.21087200
C	-6.40851300	2.53527000	-0.64039900
H	-7.96503300	1.07307000	-0.35820800
H	-4.64743600	3.76417200	-0.82081000
H	-7.09397000	3.33703500	-0.89999800
C	0.49458500	-2.38715800	1.63146600
C	0.26957900	-1.85217800	3.03953700
H	0.12836100	-3.41656200	1.55189000
H	0.59312000	-0.80563700	3.07479300
H	-0.80006800	-1.87026500	3.27524100
O	-1.45981800	-2.16228000	-1.90162800
C	-1.63471400	-3.56923200	-2.01999000
H	-2.71095100	-3.74491600	-1.99492200
H	-1.21540100	-3.93548800	-2.96553800
H	-1.16140100	-4.08892100	-1.17806000

H	1.56142300	-2.39989100	1.41326100
C	1.04192800	-2.67268600	4.07039100
H	0.73230400	-3.72444200	4.05182100
H	2.12031000	-2.63370800	3.87376600
H	0.87246700	-2.29296900	5.08331700

6°

at wb97xd/6-31+G*
Energy = -1745.449744 A.U.
Thermal correction to Gibbs Free Energy = 0.471816 A.U.
Sum of electronic and thermal Free Energies = -1744.977928 A.U.

P	2.43162100	-0.03699200	0.78183700
C	-2.42087400	-0.68647500	0.16812900
C	1.81037900	-0.98616400	-0.69370500
H	-1.88270300	0.13242900	-0.30118900
C	-3.74193400	-0.64981400	0.37626300
C	0.51052500	-1.56694800	-0.63517000
C	2.49551200	-0.94125700	-1.90725800
N	-0.19037200	-1.52881200	0.61170700
C	-0.14146400	-1.76223500	-1.86638500
C	1.87494600	-1.29964700	-3.10218200
H	3.50260500	-0.53888200	-1.93930600
C	0.54080700	-1.66821100	-3.07839500
H	2.41393200	-1.23221100	-4.04230100
H	-0.00638100	-1.88616600	-3.99099600
C	1.21606500	1.35272400	0.71761500
C	0.90308000	1.99274700	1.92022200
C	0.57599900	1.77486000	-0.45453100
C	-0.02693500	3.03066800	1.95652400
H	1.38256900	1.66872700	2.84110500
C	-0.35414700	2.81024700	-0.42082100
H	0.79830500	1.28819500	-1.40081500
C	-0.66050000	3.43891700	0.78585200
H	-0.26416800	3.51092800	2.90154800
H	-0.84252700	3.12460900	-1.33981000
H	-1.39317500	4.24097100	0.81314900
C	3.96859000	0.72665500	0.11689300
C	5.15614300	0.00727900	0.30423800
C	4.02968300	1.96535500	-0.53005900
C	6.37079100	0.49758700	-0.16842100
H	5.12906200	-0.94757000	0.82522600
C	5.24689200	2.46510100	-0.98902300
H	3.12453500	2.54719500	-0.67659900
C	6.41832600	1.73086600	-0.81582000
H	7.28052900	-0.07759600	-0.02057100
H	5.27813400	3.43099400	-1.48572400
H	7.36554800	2.12140200	-1.17705500
C	-1.59765600	-1.82446800	0.63405200
O	-2.05083100	-2.81998800	1.16444400
H	-4.20294200	-1.52108400	0.84048700
C	-4.63886500	0.46100000	0.02766400
C	-6.02163700	0.23864700	-0.00188700
C	-4.16320900	1.74673900	-0.27123500
C	-6.90468100	1.25949100	-0.34356100
H	-6.40617300	-0.74949300	0.23989400
C	-5.04382300	2.76712100	-0.60947900
H	-3.09839500	1.95460100	-0.21738300
C	-6.41804200	2.52762700	-0.65141100
H	-7.97314600	1.06452500	-0.36610200
H	-4.65825200	3.75803400	-0.83396000
H	-7.10419400	3.32783700	-0.91395400
C	0.48517600	-2.35943000	1.65816600
C	0.24811100	-1.80320900	3.05596200
H	0.12192600	-3.39068900	1.59135900
H	0.56878500	-0.75541000	3.07746900
H	-0.82337800	-1.81990500	3.28373700
O	-1.45431400	-2.16241600	-1.89285500
C	-1.64380300	-3.56734300	-2.01272100
H	-2.72185200	-3.73173000	-1.98825100
H	-1.22815700	-3.93716700	-2.95846000
H	-1.17618200	-4.09257000	-1.17109100
H	1.55358000	-2.37337300	1.44864000
C	1.01404300	-2.60580200	4.10552000
H	0.70688500	-3.65843200	4.10085200
H	2.09381200	-2.56737600	3.91660900
H	0.83587000	-2.21078200	5.11107000

7°

at wb97xd/6-31+G*
Energy = -1745.448925 A.U.
Thermal correction to Gibbs Free Energy = 0.471671 A.U.
Sum of electronic and thermal Free Energies = -1744.977254 A.U.

P	2.43485400	-0.03259600	0.78792700
C	-2.42705100	-0.68703000	0.16882700
C	1.81842500	-1.00066300	-0.67775300
H	-1.89008200	0.13135300	-0.30268800
C	-3.74820900	-0.65178400	0.37677700
C	0.51501800	-1.57465900	-0.62082900
C	2.51165600	-0.97387300	-1.88726200
N	-0.19408200	-1.51806600	0.62044900
C	-0.13079700	-1.77524000	-1.85459400
C	1.89689500	-1.34178100	-3.08226000
H	3.52117600	-0.57750300	-1.91733800
C	0.55976000	-1.69945100	-3.06314400
H	2.44252400	-1.28853700	-4.01944300
H	0.01656900	-1.92165900	-3.97711000
C	1.22002700	1.35681500	0.70188000
C	0.90695700	2.01506000	1.89460800
C	0.58048800	1.76164000	-0.47663200
C	-0.02280600	3.05363800	1.91508800
H	1.38619600	1.70491600	2.82039900
C	-0.34985300	2.79725000	-0.45864700
H	0.80309100	1.26111600	-1.41554700
C	-0.65634000	3.44411000	0.73833800
H	-0.25992600	3.54827200	2.85269800
H	-0.83817400	3.09760800	-1.38232900

H	-1.38886100	4.24664300	0.75340800
C	3.97394000	0.72288500	0.11853600
C	5.16208200	0.00966400	0.32451000
C	4.03556500	1.95032900	-0.54951300
C	6.37809900	0.49468400	-0.15020200
H	5.13441200	-0.93608100	0.86179800
C	5.25395800	2.44497600	-1.01071600
H	3.12977200	2.52722000	-0.71088700
C	6.42617300	1.71665400	-0.81862100
H	7.28838800	-0.07564200	0.01235700
H	5.28558100	3.40214800	-1.52399800
H	7.37438300	2.10315000	-1.18160600
C	-1.60119300	-1.82154100	0.63809100
O	-2.05165500	-2.81885100	1.16690100
H	-4.20827400	-1.52219100	0.84357500
C	-4.64617800	0.45715100	0.02479900
C	-6.02866300	0.23315700	-0.00555700
C	-4.17160400	1.74273500	-0.27660700
C	-6.91251400	1.25219000	-0.35052000
H	-6.41232900	-0.75484200	0.23819100
C	-5.05307100	2.76129100	-0.61814200
H	-3.10706400	1.95204700	-0.22217800
C	-6.42697400	2.52013300	-0.66089000
H	-7.98073800	1.05598300	-0.37363900
H	-4.66841400	3.75212300	-0.84451500
H	-7.11376300	3.31895400	-0.92598100
C	0.47553600	-2.33174200	1.68432600
C	0.22559600	-1.75482100	3.07141200
H	0.11527400	-3.36469600	1.62993100
H	0.54374700	-0.70606300	3.07972400
H	-0.84783100	-1.77008000	3.29048800
O	-1.44752200	-2.16225000	-1.88476400
C	-1.65220700	-3.56484100	-2.00684100
H	-2.73202700	-3.71741200	-1.98320000
H	-1.24030800	-3.93793800	-2.95288900
H	-1.19071500	-4.09614300	-1.16569200
H	1.54554600	-2.34660100	1.48408700
C	0.98400200	-2.53978000	4.13958600
H	0.67887400	-3.59296900	4.14835100
H	2.06523300	-2.50207900	3.95913300
H	0.79662600	-2.12981600	5.13745000

8°

at wb97xd/6-31+G*
Energy = -1745.447531 A.U.
Thermal correction to Gibbs Free Energy = 0.471088 A.U.
Sum of electronic and thermal Free Energies = -1744.976443 A.U.

P	-2.31935600	0.16399900	-0.93189700
C	3.18348800	-0.26456000	-0.33170200
C	-1.62255700	-0.85981500	0.45231600
H	3.12932300	0.60984400	0.30571500
C	4.34136700	-0.64888000	-0.88545500
C	-0.22674800	-1.07704400	0.57826800
C	-2.51043000	-1.56482500	1.27470500
N	0.67985700	-0.49184500	-0.36265300
C	0.19351100	-2.11702500	1.43557300
C	-2.06944300	-2.56619400	2.12813300
H	-3.57545700	-1.37275000	1.19651000
C	-0.71358100	-2.84772700	2.19694700
H	-2.77797300	-3.12410400	2.73305100
H	-0.34153100	-3.61416500	2.86930500
C	-2.65936600	1.78195700	-0.10994700
C	-2.81130700	2.89955500	-0.93998800
C	-2.75426200	1.95790500	1.27427300
C	-3.06373800	4.15983000	-0.40283700
H	-2.72707200	2.78134600	-2.01824200
C	-2.99296800	3.21968500	1.81523400
H	-2.63585400	1.10459000	1.93668700
C	-3.15017700	4.32288900	0.97860000
H	-3.18120700	5.01516000	-1.06232000
H	-3.05607600	3.33996600	2.89332700
H	-3.33478400	5.30639700	1.40151400
C	-4.01756800	-0.53367700	-1.09177600
C	-4.13890400	-1.74374600	-1.78887100
C	-5.17668100	0.08602400	-0.61556800
C	-5.38409800	-2.33289100	-1.98108200
H	-3.24777600	-2.23192800	-2.17788300
C	-6.42736500	-0.49672400	-0.82143800
H	-5.10898500	1.02735500	-0.07763100
C	-6.53435700	-1.70834400	-1.49842200
H	-5.45831900	-3.27566600	-2.51590500
H	-7.31872500	-0.00121400	-0.44616300
H	-7.50878300	-2.16250200	-1.65509200
C	1.95815600	-1.08243800	-0.54533700
O	2.03961300	-2.22627200	-0.96169200
H	4.31389900	-1.53240200	-1.52192000
C	5.65067700	0.00011300	-0.73353000
C	6.71667200	-0.44685800	-1.52562400
C	5.88689300	1.04580000	0.17196700
C	7.97710800	0.13627900	-1.42880700
H	6.55095200	-1.26013500	-2.22812800
C	7.14443600	1.62797400	0.27133500
H	5.08591200	1.40338000	0.81258700
C	8.19454600	1.17717100	-0.52997800
H	8.78866900	-0.22392300	-2.05443400
H	7.30925100	2.43476400	0.97986100
H	9.17679300	1.63382300	-0.44924600
C	0.68745800	0.99165800	-0.35912700
C	0.75558400	1.66048900	1.01386800
H	1.51949700	1.31531100	-0.99081200
H	-0.10077300	1.33808100	1.61546200
H	1.64869900	1.32840100	1.55820700
O	1.53485000	-2.24197100	1.65689200
C	2.08352700	-3.55071400	1.65113700
H	3.16148600	-3.42550400	1.53999100
H	1.87445700	-4.07104200	2.59418500
H	1.69757200	-4.12572500	0.80393600
H	-0.20220300	1.33956600	-0.88732800
C	0.73967100	3.18212300	0.88666400
H	1.59854600	3.54412600	0.30772600

H	-0.17379600	3.52051400	0.38396500
H	0.77261500	3.65937100	1.87132100

9°

at wb97xd/6-31+G*
Energy = -1745.448354 A.U.
Thermal correction to Gibbs Free Energy = 0.471084 A.U.
Sum of electronic and thermal Free Energies = -1744.97727 A.U.

P	-2.31435200	0.16362900	-0.93589700
C	3.18444300	-0.26325300	-0.32499100
C	-1.62657100	-0.86377400	0.44981400
H	3.13596500	0.61489400	0.30784700
C	4.33797800	-0.65251100	-0.88426600
C	-0.23139200	-1.07426400	0.58810800
C	-2.51824300	-1.57401400	1.26360100
N	0.68179200	-0.48565300	-0.34454500
C	0.18785200	-2.10859900	1.45276600
C	-2.07916000	-2.57242300	2.12142300
H	-3.58354300	-1.38786700	1.17556000
C	-0.72216500	-2.84488100	2.20516900
H	-2.79020400	-3.13487200	2.71917200
H	-0.35256100	-3.60832900	2.88223100
C	-2.65833500	1.77855600	-0.10932000
C	-2.80814300	2.89921900	-0.93562800
C	-2.75701900	1.94933000	1.27528300
C	-3.06236900	4.15739900	-0.39448700
H	-2.72080300	2.78507700	-2.01407400
C	-2.99761000	3.20904900	1.82026200
H	-2.64025300	1.09355600	1.93479700
C	-3.15279400	4.31529900	0.98731900
H	-3.17816300	5.01515900	-1.05110300
H	-3.06375700	3.32527700	2.89861700
H	-3.33880900	5.29719100	1.41336200
C	-4.01180400	-0.53272900	-1.10820700
C	-4.13006500	-1.73848400	-1.81322700
C	-5.17298900	0.08399400	-0.63311700
C	-5.37441400	-2.32647500	-2.01437500
H	-3.23725100	-2.22422200	-2.20142600
C	-6.42273300	-0.49749700	-0.84790000
H	-5.10755500	1.02196700	-0.08906800
C	-6.52673800	-1.70493300	-1.53281000
H	-5.44630300	-3.26594500	-2.55529500
H	-7.31572400	-0.00436100	-0.47338400
H	-7.50045700	-2.15815400	-1.69643200
C	1.95665000	-1.08099500	-0.52560800
O	2.03485600	-2.22969200	-0.92962400
H	4.30490800	-1.53999400	-1.51493400
C	5.64918500	-0.00431100	-0.74568600
C	6.70931700	-0.45877100	-1.54137900
C	5.89279700	1.04793700	0.15019500
C	7.97116500	0.12328600	-1.45731700
H	6.53784100	-1.27711800	-2.23657200
C	7.15176400	1.62907200	0.23683200
H	5.09647700	1.41170300	0.79314900
C	8.19595800	1.17068800	-0.56791400
H	8.77807100	-0.24280700	-2.08554200
H	7.32232500	2.44108200	0.93799900
H	9.17931600	1.62660400	-0.49717900
C	0.68982300	0.99726100	-0.33701500
C	0.75889400	1.66066200	1.03838100
H	1.52095900	1.32293700	-0.96872600
H	-0.09630200	1.33398200	1.63954600
H	1.65344600	1.32768500	1.57985100
O	1.52761200	-2.22302100	1.68692300
C	2.08469000	-3.52808200	1.69973300
H	3.16257400	-3.39709700	1.59465100
H	1.87246100	-4.03921100	2.64711100
H	1.70841500	-4.11489900	0.85626200
H	-0.20051900	1.34699700	-0.86275000
C	0.73985600	3.18277900	0.91740600
H	1.59725900	3.54883300	0.33883000
H	-0.17492600	3.52136200	0.41719300
H	0.77314400	3.65618600	1.90390800

10°

at wb97xd/6-31+G*
Energy = -1745.449169 A.U.
Thermal correction to Gibbs Free Energy = 0.47162 A.U.
Sum of electronic and thermal Free Energies = -1744.977549 A.U.

P	-2.30990200	0.16247800	-0.93978600
C	3.18503100	-0.26099600	-0.32051500
C	-1.62996000	-0.86752200	0.44753900
H	3.14170500	0.62203000	0.30596100
C	4.33484800	-0.65633300	-0.88314200
C	-0.23536200	-1.07124300	0.59716900
C	-2.52471700	-1.58237100	1.25387700
N	0.68361300	-0.47911800	-0.32774200
C	0.18325100	-2.09966600	1.46914200
C	-2.08699800	-2.57717000	2.11658200
H	-3.59020900	-1.40205100	1.15670800
C	-0.72906800	-2.84064500	2.21410000
H	-2.79995700	-3.14347200	2.70840000
H	-0.36136100	-3.60054700	2.89614200
C	-2.65750100	1.77486600	-0.10954600
C	-2.80807000	2.89753900	-0.93307400
C	-2.75668800	1.94201000	1.27542900
C	-3.06373200	4.15402900	-0.38880800
H	-2.72011400	2.78631000	-2.01177400
C	-2.99862500	3.20016600	1.82353700
H	-2.63938700	1.08464000	1.93274600
C	-3.15472400	4.30834600	0.99341600
H	-3.18016200	5.01335000	-1.04326800
H	-3.06505100	3.31357000	2.90217300
H	-3.34181500	5.28897300	1.42189000
C	-4.00665100	-0.53272300	-1.12293800
C	-4.12228700	-1.73407200	-1.83581800

C	-5.16952300	0.08074300	-0.64769800
C	-5.36585400	-2.32113200	-2.04460300
H	-3.22810300	-2.21710500	-2.22423000
C	-6.41838500	-0.49970200	-0.87007700
H	-5.10595600	1.01530800	-0.09758100
C	-6.51984500	-1.70289200	-1.56285400
H	-5.43576500	-3.25722200	-2.59160300
H	-7.31271800	-0.00917400	-0.49534200
H	-7.49291300	-2.15533200	-1.73239800
C	1.95502700	-1.07871000	-0.50804000
O	2.03017000	-2.23208000	-0.89985600
H	4.29689400	-1.54863400	-1.50669800
C	5.64774400	-0.00897500	-0.75696800
C	6.70242100	-0.47024700	-1.55598300
C	5.89814500	1.04906100	0.13017100
C	7.96545100	0.11082000	-1.48368400
H	6.52567900	-1.29314300	-2.24444900
C	7.15831300	1.62922100	0.20508900
H	5.10619000	1.41817300	0.77548000
C	8.19697400	1.16403500	-0.60291500
H	8.76803700	-0.26058000	-2.11432500
H	7.33417100	2.44580600	0.89960400
H	9.18129300	1.61921100	-0.54137100
C	0.69155600	1.00316100	-0.31632000
C	0.76300700	1.66135000	1.06130600
H	1.52077500	1.33114700	-0.94922800
H	-0.08906800	1.32846700	1.66375200
H	1.66087000	1.32950000	1.59804500
O	1.52145200	-2.20418000	1.71517100
C	2.08616500	-3.50574600	1.74334900
H	3.16401700	-3.36913600	1.64532800
H	1.86988600	-4.01010200	2.69344200
H	1.71981100	-4.10172900	0.90195600
H	-0.20022700	1.35436200	-0.83844500
C	0.73706500	3.18385100	0.94651500
H	1.58966600	3.55576300	0.36459400
H	-0.18202300	3.52046800	0.45286500
H	0.77403200	3.65355200	1.93466800

30°

at wb97xd/6-31+G*
Energy = -1745.462792 A.U.
Thermal correction to Gibbs Free Energy = 0.472376 A.U.
Sum of electronic and thermal Free Energies = -1744.990416 A.U.

P	2.22011900	0.11010300	0.99671400
C	-3.17715300	-0.20953800	0.25313200
C	1.65491000	-0.89390300	-0.45383500
H	-3.21695100	0.73455900	-0.27871700
C	-4.25954900	-0.70760300	0.86498000
C	0.28520800	-0.96868200	-0.77405100
C	2.57460700	-1.65323800	-1.18934400
N	-0.70741100	-0.32422800	0.02898000
C	-0.12421100	-1.84784600	-1.79897000
C	2.15180300	-2.53143900	-2.17702800
H	3.63204100	-1.58799100	-0.95532900
C	0.80002100	-2.63134100	-2.48204100
H	2.87614100	-3.13452400	-2.71681100
H	0.46265800	-3.29755600	-3.26929700
C	2.67769800	1.70505300	0.18200600
C	2.82990900	2.82508500	1.00932400
C	2.83813900	1.86534900	-1.19830100
C	3.14941900	4.07055900	0.47420500
H	2.69154900	2.72086200	2.08343300
C	3.14644800	3.11332700	-1.73716600
H	2.71799100	1.01192600	-1.85982200
C	3.30469800	4.21805000	-0.90331700
H	3.26595200	4.92750400	1.13179700
H	3.26119800	3.22112500	-2.81231600
H	3.54255800	5.19068600	-1.32481900
C	3.87471600	-0.62577900	1.33459400
C	3.89727900	-1.80633300	2.08954600
C	5.08900800	-0.06773400	0.92344900
C	5.10183100	-2.42745200	2.40394600
H	2.96154000	-2.24619400	2.42813100
C	6.29764300	-0.68168700	1.25156400
H	5.09731300	0.84913700	0.34094300
C	6.30756300	-1.86406400	1.98673400
H	5.09999800	-3.34697500	2.98259100
H	7.23287700	-0.23427600	0.92595200
H	7.24976900	-2.34273000	2.23863500
C	-1.91342600	-0.99954900	0.23699900
O	-1.92719300	-2.20310600	0.45535700
H	-4.14025900	-1.66577600	1.36919800
C	-5.59776100	-0.10413700	0.93235500
C	-6.58889500	-0.75583700	1.67838700
C	-5.93443400	1.09336100	0.28281500
C	-7.87457300	-0.23117000	1.77891300
H	-6.34465000	-1.68570200	2.18632300
C	-7.21694900	1.61841900	0.38105900
H	-5.19134500	1.62104500	-0.30812600
C	-8.19271900	0.95888700	1.13016800
H	-8.62680800	-0.75278600	2.36369600
H	-7.45900700	2.54609600	-0.12973400
H	-9.19451500	1.37201400	1.20508100
C	-0.70889700	1.14699500	-0.00925600
C	-0.82562800	1.75968400	-1.40275300
H	-1.50539800	1.49999300	0.65030700
H	-0.01768700	1.36522300	-2.03111700
H	-1.76436200	1.44625600	-1.87710400
O	-1.43113900	-1.79388700	-2.17188500
C	-2.07732700	-3.01654600	-2.48631700
H	-3.14731400	-2.80519900	-2.46517100
H	-1.79894500	-3.36844700	-3.48762300
H	-1.84281200	-3.77643400	-1.73416300
H	0.21110400	1.50335700	0.45698400
C	-0.72835600	3.28271800	-1.34158200
H	-1.53542100	3.71033000	-0.73361300
H	0.22644000	3.59327700	-0.90034700
H	-0.79303700	3.72354000	-2.34160800

50°

at wb97xd/6-31+G*
Energy = -1745.4706560000002 A.U.
Thermal correction to Gibbs Free Energy = 0.467591 A.U.
Sum of electronic and thermal Free Energies = -1745.003065 A.U.

P	-2.01700500	0.10554900	-0.98886500
C	3.03843500	-0.10701600	-0.27571300
C	-1.67835700	-0.84323600	0.56436100
H	3.07274300	0.96739000	-0.14242500
C	4.10905200	-0.77431000	-0.72575600
C	-0.37312000	-0.84897300	1.07732400
C	-2.66108400	-1.62151400	1.18949300
N	0.67523600	-0.13551200	0.41814500
C	-0.05787200	-1.66437700	2.18014600
C	-2.33881200	-2.42918200	2.27190900
H	-3.67527500	-1.62288800	0.80298000
C	-1.03900500	-2.45831600	2.76774900
H	-3.10462100	-3.04425700	2.73605400
H	-0.79316100	-3.08810000	3.61636400
C	-2.66251800	1.70216900	-0.32190400
C	-2.71174100	2.78763600	-1.20528000
C	-3.07288600	1.89589100	1.00188600
C	-3.17398800	4.03180500	-0.78292100
H	-2.38094300	2.65733200	-2.23344200
C	-3.52224100	3.14368800	1.43018600
H	-3.03623100	1.06950000	1.70659300
C	-3.57688100	4.21356700	0.53899500
H	-3.20895400	4.86166000	-1.48330200
H	-3.83231900	3.27819900	2.46295900
H	-3.92780700	5.18563700	0.87401700
C	-3.56945600	-0.70657300	-1.56257000
C	-3.42723700	-1.91751500	-2.25369400
C	-4.85333800	-0.18461200	-1.37874300
C	-4.54203300	-2.60186500	-2.72773500
H	-2.43397800	-2.33137800	-2.41479500
C	-5.97020200	-0.86261100	-1.86712200
H	-4.98849700	0.75463500	-0.84990800
C	-5.81870500	-2.07360500	-2.53730500
H	-4.41363600	-3.54380900	-3.25369100
H	-6.96116300	-0.44241900	-1.71772900
H	-6.68995000	-2.60199500	-2.91430800
C	1.79641200	-0.86206500	0.05602000
O	1.75819000	-2.08392100	-0.03616800
H	3.99756300	-1.85028700	-0.85271300
C	5.41758800	-0.20702300	-1.07981400
C	6.34419900	-1.02238600	-1.74328700
C	5.78739700	1.11327600	-0.78167500
C	7.59421700	-0.53456900	-2.11410400
H	6.07520800	-2.05008000	-1.97520000
C	7.03555900	1.60154000	-1.14837000
H	5.10015800	1.76337700	-0.24763000
C	7.94355100	0.78056900	-1.81866500
H	8.29531100	-1.18282200	-2.63194100
H	7.30504100	2.62576700	-0.90645400
H	8.91894200	1.16462400	-2.10332900
C	0.71388700	1.31257300	0.65554200
C	1.31397900	1.71798200	2.00175900
H	1.23020400	1.79689800	-0.17850100
H	0.74636200	1.22833000	2.80171100
H	2.34132600	1.34255800	2.08251200
O	1.20663200	-1.57693600	2.66400500
C	1.80042100	-2.76224400	3.16279400
H	2.86823100	-2.55325800	3.23993900
H	1.41290500	-3.01975900	4.15668200
H	1.63989100	-3.59108500	2.46505100
H	-0.31394700	1.67841100	0.61064700
C	1.28050000	3.23432600	2.18393300
H	1.86745000	3.74131800	1.40794900
H	0.25360600	3.61499800	2.12443100
H	1.69106300	3.52550200	3.15611400

70°

at wb97xd/6-31+G*
Energy = -1745.474224 A.U.
Thermal correction to Gibbs Free Energy = 0.468941 A.U.
Sum of electronic and thermal Free Energies = -1745.005283 A.U.

P	-1.81817200	0.55100700	-0.87364200
C	2.84265000	-0.07516900	-0.32199600
C	-1.75364800	-1.04825100	0.05497000
H	2.90493100	0.79675300	0.31906800
C	3.86169400	-0.41573600	-1.12084300
C	-0.53004000	-1.40985900	0.62520900
C	-2.83668200	-1.93273500	0.14275300
N	0.59354700	-0.53121200	0.54116700
C	-0.37338700	-2.66588500	1.23852400
C	-2.68081800	-3.16440000	0.76324900
H	-3.79402800	-1.66606300	-0.29343800
C	-1.45475600	-3.54274700	1.30685000
H	-3.52176500	-3.84990800	0.82154200
H	-1.35275500	-4.51554000	1.77466400
C	-2.25363800	1.73060800	0.47736600
C	-1.86353400	3.06444600	0.31797600
C	-2.91771700	1.36051300	1.65321600
C	-2.13505900	4.01048600	1.30525600
H	-1.33389300	3.36294900	-0.58394900
C	-3.18248200	2.30150500	2.64502400
H	-3.22065000	0.32669800	1.79907100
C	-2.79160500	3.62920100	2.47313700
H	-1.82473100	5.04229700	1.16556300
H	-3.69442400	1.99757900	3.55409600
H	-2.99593700	4.36238000	3.24846300
C	-3.42492100	0.37543500	-1.76050300
C	-3.41208900	-0.39644800	-2.93081500
C	-4.62293400	0.98006500	-1.36905000
C	-4.57292900	-0.58165900	-3.67499500
H	-2.48464500	-0.86296500	-3.25665000

C	-5.78366100	0.80800400	-2.12345300
H	-4.65696700	1.59024600	-0.47108800
C	-5.76379300	0.02329800	-3.27330100
H	-4.54606500	-1.19096100	-4.57414700
H	-6.70591500	1.28753300	-1.80655600
H	-6.66949000	-0.11248000	-3.85784900
C	1.60331600	-0.90337600	-0.32172600
O	1.47050800	-1.85882300	-1.07757800
H	3.71685400	-1.29079300	-1.75306200
C	5.15250500	0.27582900	-1.24645700
C	6.00901400	-0.08575200	-2.29484400
C	5.57245900	1.28073900	-0.36191200
C	7.23803400	0.54482300	-2.46840900
H	5.70116900	-0.86788200	-2.98461700
C	6.79992800	1.90957900	-0.53139700
H	4.94171600	1.56713000	0.47492600
C	7.63718700	1.54635300	-1.58717400
H	7.88404100	0.25170500	-3.29104200
H	7.10896000	2.68352300	0.16560000
H	8.59675600	2.03866300	-1.71679900
C	0.73269600	0.49361800	1.58016300
C	1.56978800	0.07275800	2.78875100
H	1.12549200	1.41247600	1.12992300
H	1.09873500	-0.79663400	3.25956800
H	2.56164500	-0.25797000	2.45717000
O	0.85404800	-2.93078700	1.74348600
C	1.14228200	-4.25057300	2.15174600
H	2.20152000	-4.25635500	2.41073800
H	0.55290600	-4.53681900	3.03263800
H	0.96191800	-4.96185500	1.33668700
H	-0.27644100	0.74335400	1.91605300
C	1.69966800	1.21882400	3.79035100
H	2.20405400	2.08356500	3.34138600
H	0.71478200	1.55466800	4.13692300
H	2.27836300	0.91400900	4.66832400

90°

at wb97xd/6-31+G*
Energy = -1745.474696 A.U.
Thermal correction to Gibbs Free Energy = 0.470252 A.U.
Sum of electronic and thermal Free Energies = -1745.004444 A.U.

P	-1.58311600	-1.02241200	0.30565200
C	2.63306600	-0.01978100	0.40463300
C	-1.83838200	0.76737500	0.69985600
H	2.73169800	-0.18133100	-0.66326700
C	3.59517800	-0.40518400	1.25173800
C	-0.73847200	1.61357600	0.53341300
C	-3.03773400	1.29298800	1.19358800
N	0.48960700	1.09460100	0.01706400
C	-0.82869700	2.97408100	0.86793500
C	-3.12416000	2.64131100	1.51537500
H	-3.90047400	0.64899100	1.33024600
C	-2.02955500	3.48823700	1.35821100
H	-4.05723500	3.04599700	1.89766200
H	-2.12027500	4.53515700	1.62475000
C	-1.78238600	-0.99325200	-1.52800200
C	-1.09262900	-1.96054800	-2.26610200
C	-2.54413000	-0.04036200	-2.21519000
C	-1.16274300	-1.98062000	-3.65839500
H	-0.48563500	-2.69806800	-1.74566400
C	-2.61112700	-0.05365400	-3.60590700
H	-3.07925400	0.72504300	-1.65836900
C	-1.91893900	-1.02351900	-4.33094600
H	-0.61933500	-2.73802100	-4.21655800
H	-3.20290300	0.69550800	-4.12506200
H	-1.96814800	-1.03097700	-5.41630200
C	-3.18384500	-1.74097500	0.86884700
C	-3.28986900	-2.03179900	2.23618100
C	-4.26504800	-2.03876600	0.03422900
C	-4.45693500	-2.57958300	2.75999700
H	-2.45134100	-1.82013500	2.89649200
C	-5.42912400	-2.60304700	0.55566800
H	-4.20383700	-1.83283400	-1.03046900
C	-5.53086100	-2.86898900	1.91868600
H	-4.52424100	-2.78984600	3.82382100
H	-6.25892400	-2.83175600	-0.10768000
H	-6.43954800	-3.30559800	2.32377700
C	1.39392700	0.61325500	0.93820400
O	1.17242200	0.68247600	2.14165600
H	3.41059500	-0.23754100	2.31206700
C	4.86883700	-1.04972300	0.90134500
C	5.64000600	-1.61621400	1.92490600
C	5.35317500	-1.12143200	-0.41347200
C	6.84716100	-2.25150100	1.64672200
H	5.28190900	-1.56171400	2.95011700
C	6.55937600	-1.75233100	-0.69313000
H	4.79090500	-0.66819100	-1.22511600
C	7.31031900	-2.32301400	0.33542700
H	7.42638200	-2.68849900	2.45512700
H	6.91985400	-1.79481000	-1.71713900
H	8.25347900	-2.81450800	0.11438100
C	0.74131500	1.19762100	-1.42384700
C	1.75764500	2.26678000	-1.82839500
H	1.03601200	0.21587300	-1.81586200
H	1.38134900	3.24658500	-1.51978100
H	2.69790900	2.11080800	-1.28564600
O	0.29625400	3.70733900	0.68696100
C	0.30986100	5.04177700	1.14601100
H	1.32018500	5.40850900	0.96126300
H	-0.40791800	5.66341000	0.59513600
H	0.09519600	5.09227300	2.22052000
H	-0.22131900	1.41855100	-1.89198100
C	2.01109400	2.23504700	-3.33462400
H	2.42007700	1.26667000	-3.64889200
H	1.08321000	2.39836100	-3.89617200
H	2.72377000	3.01102300	-3.63233300

110°

at wb97xd/6-31+G*
Energy = -1745.473684 A.U.
Thermal correction to Gibbs Free Energy = 0.470894 A.U.
Sum of electronic and thermal Free Energies = -1745.00279 A.U.

P	1.38365300	-1.11938200	-0.39368800
C	-2.34425100	0.17627600	-0.59680100
C	2.00663800	0.59551200	-0.71695100
H	-2.45312400	0.09077100	0.47971500
C	-3.27579300	-0.30270600	-1.42970500
C	1.08630800	1.64918600	-0.63153900
C	3.33010300	0.87277900	-1.07383300
N	-0.26244400	1.39630000	-0.23391900
C	1.50247700	2.96994900	-0.86125500
C	3.72950400	2.18122400	-1.31668900
H	4.05508100	0.06904900	-1.14814300
C	2.82822000	3.23575500	-1.20655200
H	4.76099600	2.38920000	-1.58709600
H	3.16295200	4.24917600	-1.39642000
C	1.06655300	-1.00750600	1.41878000
C	-0.00578300	-1.74785400	1.92749700
C	1.78762300	-0.18222300	2.29000700
C	-0.35181900	-1.66786100	3.27601400
H	-0.58593200	-2.37785100	1.25695500
C	1.44327700	-0.09833800	3.63630300
H	2.61299100	0.41324900	1.90673400
C	0.37049200	-0.83930100	4.13190700
H	-1.19009900	-2.24652800	3.65434400
H	2.00913600	0.55068100	4.29933300
H	0.09817000	-0.76842400	5.18134000
C	2.94095900	-2.09920200	-0.48785700
C	3.30230600	-2.56736000	-1.75872600
C	3.75406600	-2.42534600	0.60203300
C	4.46200600	-3.31571900	-1.94091600
H	2.66943400	-2.33906800	-2.61376800
C	4.90697600	-3.18868400	0.42360800
H	3.48875600	-2.08860400	1.59979300
C	5.26754000	-3.62994000	-0.84740900
H	4.73014500	-3.66278900	-2.93493900
H	5.52514800	-3.43696100	1.28214900
H	6.16785100	-4.22241200	-0.98471900
C	-1.09625000	0.77769700	-1.14421800
O	-0.80074200	0.69126300	-2.32933400
H	-3.07472900	-0.21075800	-2.49631800
C	-4.53479100	-0.96156600	-1.05434400
C	-5.28928300	-1.59021500	-2.05366800
C	-5.02022700	-0.98966000	0.26193700
C	-6.48254400	-2.24038500	-1.75073600
H	-4.92939700	-1.57199800	-3.07953400
C	-6.21237800	-1.63552600	0.56612600
H	-4.46929400	-0.49218500	1.05524500
C	-6.94768300	-2.26625700	-0.43852200
H	-7.04915400	-2.72501800	-2.54070800
H	-6.57400000	-1.64353800	1.59060300
H	-7.87985200	-2.76953500	-0.19826100
C	-0.67910000	1.80442400	1.11402500
C	-1.80971200	2.83250500	1.15585800
H	-0.94122900	0.92041800	1.71095400
H	-1.49998300	3.71604600	0.59089000
H	-2.70123800	2.43444000	0.65728100
O	0.54798600	3.92447400	-0.72506000
C	0.88631800	5.26284900	-1.01731600
H	-0.02871800	5.83855500	-0.87200000
H	1.66277200	5.63785700	-0.33815500
H	1.22091100	5.36981000	-2.05653800
H	0.20846800	2.22836500	1.59081600
C	-2.15108700	3.20407400	2.59806900
H	-2.46553000	2.32354900	3.17211900
H	-1.28499000	3.64051200	3.11036700
H	-2.96515100	3.93524800	2.63510900

130°

at wb97xd/6-31+G*
Energy = -1745.470536 A.U.
Thermal correction to Gibbs Free Energy = 0.472763 A.U.
Sum of electronic and thermal Free Energies = -1744.997773 A.U.

P	1.16080200	-1.20409300	-0.49488400
C	-2.19156900	0.50832300	-0.75240200
C	2.07483600	0.39793200	-0.71614300
H	-2.42582800	0.71068500	0.28608800
C	-3.02808200	-0.20771900	-1.51322900
C	1.36170900	1.61066400	-0.69512200
C	3.46316700	0.43019400	-0.88905700
N	-0.04534000	1.65258900	-0.45200400
C	2.06561800	2.82854400	-0.73136900
C	4.13995600	1.63945400	-0.97544900
H	4.02725300	-0.49552900	-0.92265700
C	3.45185000	2.84401500	-0.88048800
H	5.21932900	1.64776500	-1.09762600
H	3.99608300	3.78006400	-0.93066600
C	0.58626900	-0.98100400	1.24510800
C	-0.66158200	-1.51655200	1.58138800
C	1.29593300	-0.26762900	2.21928000
C	-1.18963200	-1.34585400	2.86088400
H	-1.23331700	-2.05422800	0.82846800
C	0.77229600	-0.09674900	3.49773200
H	2.25802200	0.17299700	1.96814200
C	-0.47412700	-0.63345000	3.82071100
H	-2.16127900	-1.76744400	3.10476600
H	1.33377600	0.46227400	4.24149400
H	-0.88570000	-0.49436800	4.81661300
C	2.54833700	-2.40467500	-0.32351600
C	3.00399500	-2.99179000	-1.51204700
C	3.14298900	-2.78824500	0.88262900
C	4.04804000	-3.91291700	-1.49996600
H	2.53671900	-2.72076400	-2.45646300
C	4.17691000	-3.72304400	0.89827700

H	2.79760400	-2.36298000	1.82005100
C	4.63697500	-4.28213000	-0.29181200
H	4.39302100	-4.35058700	-2.43262800
H	4.62440700	-4.01321200	1.84508200
H	5.44492900	-5.00838200	-0.27744100
C	-0.88334300	0.95458100	-1.30553900
O	-0.54396700	0.68523000	-2.45033300
H	-2.74172800	-0.36974000	-2.55144700
C	-4.29782700	-0.80340800	-1.07404600
C	-5.20117500	-1.26473900	-2.04048700
C	-4.64082600	-0.94461900	0.27949600
C	-6.41991900	-1.82775000	-1.67213700
H	-4.94341400	-1.17386600	-3.09287500
C	-5.85571600	-1.50934900	0.64911000
H	-3.94590500	-0.62492500	1.05148200
C	-6.75225100	-1.94999900	-0.32520400
H	-7.10827100	-2.17411200	-2.43775000
H	-6.10327100	-1.61327700	1.70192500
H	-7.70032800	-2.39294000	-0.03384500
C	-0.50141500	2.20589700	0.83174000
C	-1.50739300	3.34722100	0.69713200
H	-0.90344300	1.40589800	1.46712000
H	-1.05497500	4.13758400	0.09113100
H	-2.39830200	3.00577200	0.15684500
O	1.30734900	3.95172300	-0.63449900
C	1.94438300	5.20755200	-0.71731900
H	1.14949700	5.94923000	-0.62738600
H	2.66483000	5.34723700	0.09889500
H	2.45074300	5.33426700	-1.68227800
H	0.38867000	2.57236800	1.34771000
C	-1.91317800	3.88195500	2.06947400
H	-2.37094200	3.09606900	2.68304600
H	-1.04374900	4.26584500	2.61713300
H	-2.63750600	4.69770200	1.97724800

150°

at wb97xd/6-31+G*
Energy = -1745.462003 A.U.
Thermal correction to Gibbs Free Energy = 0.47301 A.U.
Sum of electronic and thermal Free Energies = -1744.988993 A.U.

P	1.07589500	-1.26108900	-0.58961400
C	-2.12151400	0.64074700	-0.83906000
C	2.08781200	0.29455200	-0.76499600
H	-2.41207000	1.03345500	0.12653600
C	-2.92342500	-0.20988900	-1.49210700
C	1.48327400	1.57242500	-0.78787400
C	3.48617900	0.20737000	-0.78135700
N	0.07008900	1.78134900	-0.67473700
C	2.30803300	2.70689300	-0.61927700
C	4.28089900	1.34072000	-0.70311400
H	3.96406200	-0.76538400	-0.79992700
C	3.69599100	2.59587800	-0.59201200
H	5.36294300	1.24687800	-0.69738100
H	4.32151100	3.47575400	-0.49792400
C	0.38726100	-0.94290000	1.09756300
C	-0.88306500	-1.45477100	1.38263700
C	1.04844300	-0.20283500	2.08663300
C	-1.48112400	-1.23414400	2.62290900
H	-1.41851500	-2.01425800	0.61972200
C	0.45508700	0.01871000	3.32624900
H	2.03039200	0.21599800	1.87930500
C	-0.81386700	-0.49390700	3.59623600
H	-2.46977200	-1.63867900	2.82311700
H	0.98105600	0.59735900	4.08095600
H	-1.27929800	-0.31623200	4.56193100
C	2.37908700	-2.52149800	-0.24980000
C	2.86393500	-3.21530400	-1.36699200
C	2.88074600	-2.85395200	1.01271300
C	3.84789900	-4.19144000	-1.23158500
H	2.46686400	-2.98474800	-2.35333400
C	3.85309300	-3.84254900	1.15234200
H	2.51024500	-2.34477100	1.89720900
C	4.34462300	-4.50824300	0.03139200
H	4.21723700	-4.71217000	-2.11073800
H	4.22846600	-4.09114500	2.14139000
H	5.10487900	-5.27645100	0.14234800
C	-0.78302300	0.96128300	-1.40551300
O	-0.43695800	0.51508800	-2.49159800
H	-2.58552800	-0.56739600	-2.46354700
C	-4.21049000	-0.72577600	-1.00713500
C	-5.04741100	-1.40773400	-1.90048600
C	-4.63126300	-0.58575600	0.32464900
C	-6.27599300	-1.91526600	-1.48716200
H	-4.72911900	-1.53499200	-2.93233300
C	-5.85613300	-1.09451400	0.73964200
H	-3.98966700	-0.08965800	1.04767400
C	-6.68535200	-1.75863600	-0.16520300
H	-6.91168600	-2.43623500	-2.19736500
H	-6.16447000	-0.97844800	1.77501200
H	-7.64137900	-2.15713400	0.16208400
C	-0.40682400	2.41851800	0.56938700
C	-1.33855300	3.60091800	0.32260800
H	-0.88062000	1.67112700	1.21701200
H	-0.80475900	4.33750800	-0.28581500
H	-2.20948600	3.28150300	-0.26260900
O	1.66114400	3.90316300	-0.56050600
C	2.42161300	5.08579900	-0.45582000
H	1.69705100	5.90080000	-0.42423200
H	3.02185600	5.09898100	0.46313300
H	3.07697700	5.21739600	-1.32588800
H	0.46938700	2.76183800	1.11900700
C	-1.80195400	4.22029500	1.63984100
H	-2.34358700	3.49006200	2.25401500
H	-0.94922000	4.58036900	2.22846200
H	-2.47017700	5.06958800	1.46452600

170°

at wb97xd/6-31+G*
Energy = -1745.449253 A.U.

Thermal correction to Gibbs Free Energy = 0.475765 A.U.
Sum of electronic and thermal Free Energies = -1744.973488 A.U.

P	-1.52200200	1.21412600	-1.00131900
C	2.12107600	-0.69249200	-0.62963000
C	-2.21207600	-0.49533900	-0.69188300
H	2.51609600	-1.27985800	0.18725300
C	2.86590200	0.27886600	-1.17285000
C	-1.50730700	-1.73150800	-0.62753300
C	-3.61367700	-0.52034500	-0.55297200
N	-0.07331100	-1.88985400	-0.59682600
C	-2.25359000	-2.88520900	-0.25044600
C	-4.31310200	-1.66304400	-0.22233700
H	-4.17106400	0.40183800	-0.65811400
C	-3.62597300	-2.85541600	-0.04599000
H	-5.39048200	-1.62699700	-0.09225300
H	-4.16451000	-3.75749200	0.21749700
C	-0.26676400	1.40245100	0.34059800
C	0.69438100	2.40587300	0.17338400
C	-0.20618700	0.60735300	1.48717300
C	1.69243400	2.60642300	1.12197700
H	0.67287300	3.02024400	-0.72373000
C	0.80073600	0.79504000	2.43421300
H	-0.94304500	-0.17812900	1.63926100
C	1.75326400	1.79432500	2.25311500
H	2.43890100	3.38040900	0.96726400
H	0.83579000	0.15960100	3.31598100
H	2.54240300	1.93879000	2.98591200
C	-2.84557600	2.29005300	-0.25904500
C	-3.55883500	3.09517600	-1.15336500
C	-3.14924000	2.38051300	1.10566000
C	-4.56007600	3.95759600	-0.70421300
H	-3.32747100	3.04502100	-2.21512700
C	-4.14342000	3.24114000	1.55891600
H	-2.60427100	1.77307700	1.82268100
C	-4.85420800	4.03108100	0.65376600
H	-5.10377700	4.57318700	-1.41568300
H	-4.36449800	3.29875900	2.62145000
H	-5.62975800	4.70394100	1.00948300
C	0.73826600	-0.90221200	-1.14221400
O	0.34076800	-0.25137300	-2.10513600
H	2.43911300	0.82364000	-2.01322100
C	4.20691800	0.68489300	-0.73370600
C	5.00848500	1.43466900	-1.60426700
C	4.71214800	0.37349100	0.53717700
C	6.28769300	1.83828900	-1.23132600
H	4.62347100	1.69594600	-2.58710400
C	5.98777700	0.77764500	0.91230800
H	4.09057900	-0.16360100	1.24800100
C	6.78261000	1.50835900	0.02811000
H	6.89614200	2.41331600	-1.92351800
H	6.36143000	0.53167900	1.90247000
H	7.77814600	1.82614900	0.32461700
C	0.47848600	-2.59596800	0.59069400
C	1.39601500	-3.75880600	0.22893200
H	0.99841000	-1.87200700	1.22573300
H	0.82833100	-4.46972500	-0.37798100
H	2.22523400	-3.40626000	-0.39609500
O	-1.56068200	-4.06155000	-0.24178800
C	-2.22995000	-5.25846900	0.08055100
H	-1.46811100	-6.03900400	0.04533900
H	-2.66316000	-5.22131600	1.08863800
H	-3.01615600	-5.48770100	-0.64975400
H	-0.34655500	-2.96162600	1.19592200
C	1.94510100	-4.43777400	1.48229900
H	2.52656300	-3.73699200	2.09469700
H	1.13297900	-4.82931700	2.10737100
H	2.60104100	-5.27503400	1.22337700

171°

at wb97xd/6-31+G*
Energy = -1745.448326 A.U.
Thermal correction to Gibbs Free Energy = 0.475813 A.U.
Sum of electronic and thermal Free Energies = -1744.972513 A.U.

P	1.52327300	-1.21306900	-1.00405600
C	-2.12148400	0.69142000	-0.63302100
C	2.21159900	0.49737300	-0.69340200
H	-2.52041600	1.28564500	0.17690400
C	-2.86304100	-0.28656300	-1.16890500
C	1.50675800	1.73403400	-0.63132800
C	3.61239700	0.52142600	-0.54582000
N	0.07217900	1.89337500	-0.60805800
C	2.25166500	2.88527300	-0.24299400
C	4.31036800	1.66204600	-0.20550700
H	4.17003300	-0.40057400	-0.65071200
C	3.62240600	2.85372100	-0.02841100
H	5.38680700	1.62479600	-0.06823900
H	4.15929400	3.75440200	0.24302400
C	0.26833700	-1.40149100	0.33830700
C	-0.69000200	-2.40803700	0.17377900
C	0.20476600	-0.60281300	1.48223700
C	-1.68832400	-2.60798400	1.12221500
H	-0.66608000	-3.02551900	-0.72114700
C	-0.80254000	-0.78991000	2.42904500
H	0.93969000	0.18479900	1.63261700
C	-1.75234300	-1.79219300	2.25053600
H	-2.43258000	-3.38450000	0.96951700
H	-0.83992700	-0.15161700	3.30865400
H	-2.54182800	-1.93615200	2.98305000
C	2.84680700	-2.28851000	-0.26064100
C	3.56308700	-3.09106200	-1.15488500
C	3.14724500	-2.38157200	1.10461500
C	4.56424700	-3.95325700	-0.70514400
H	3.33412400	-3.03907200	-2.21708800
C	4.14127100	-3.24207700	1.55846800
H	2.59986200	-1.77623000	1.82158000
C	4.85518200	-4.02927200	0.65339700
H	5.11034300	-4.56676400	-1.41658100

H	4.35978400	-3.30173400	2.62142400
H	5.63061700	-4.70202000	1.00958000
C	-0.73829800	0.89977200	-1.14534800
O	-0.34025900	0.24392800	-2.10466800
H	-2.43369000	-0.83803800	-2.00353700
C	-4.20415400	-0.69097700	-0.72856300
C	-5.00415000	-1.44728900	-1.59488800
C	-4.71085800	-0.37157600	0.53973200
C	-6.28340200	-1.84944500	-1.22053000
H	-4.61790500	-1.71482700	-2.57555600
C	-5.98654000	-0.77425900	0.91628800
H	-4.09022900	0.17048000	1.24761900
C	-6.77987500	-1.51148300	0.03617200
H	-6.89068600	-2.42960500	-1.90945400
H	-6.36135600	-0.52209300	1.90444800
H	-7.77544300	-1.82813000	0.33379100
C	-0.48334800	2.60142200	0.57740500
C	-1.40015900	3.76296300	0.21024600
H	-1.00471500	1.87809400	1.21181500
H	-0.83022300	4.47363100	-0.39492700
H	-2.22618800	3.40901100	-0.41824300
O	1.56104900	4.06323000	-0.23802600
C	2.22977500	5.25852800	0.09122800
H	1.46967900	6.04047700	0.05023300
H	2.65421800	5.21973300	1.10298400
H	3.02265900	5.48702000	-0.63206500
H	0.33886900	2.96859700	1.18519500
C	-1.95550800	4.44303100	1.46024500
H	-2.53951500	3.74260200	2.07063200
H	-1.14653700	4.83560900	2.08874900
H	-2.61061900	5.27970300	1.19736000

172°

at wb97xd/6-31+G*
Energy = -1745.447373 A.U.
Thermal correction to Gibbs Free Energy = 0.475664 A.U.
Sum of electronic and thermal Free Energies = -1744.971709 A.U.

P	1.52482000	-1.21175900	-1.00649200
C	-2.12200200	0.68975000	-0.63631500
C	2.21108200	0.49973700	-0.69454100
H	-2.52478900	1.29061100	0.16670600
C	-2.86026700	-0.29473700	-1.16489500
C	1.50586300	1.73677000	-0.63535300
C	3.61095800	0.52315100	-0.53762000
N	0.07068200	1.89674900	-0.61961800
C	2.24901900	2.88581100	-0.23608400
C	4.30701900	1.66189400	-0.18770400
H	4.16906300	-0.39859500	-0.64164100
C	3.61793200	2.85284300	-0.01078300
H	5.38240200	1.62365300	-0.04269600
H	4.15285400	3.75237700	0.26815900
C	0.27040800	-1.40042800	0.33649600
C	-0.68491600	-2.41033500	0.17498600
C	0.20375300	-0.59792900	1.47758200
C	-1.68340300	-2.60962900	1.12337500
H	-0.65851600	-3.03110900	-0.71759900
C	-0.80387400	-0.78436700	2.42424800
H	0.93660800	0.19197000	1.62602000
C	-1.75075900	-1.78985000	2.24864100
H	-2.42527400	-3.38888100	0.97298500
H	-0.84367400	-0.14299600	3.30151100
H	-2.54054800	-1.93328300	2.98092100
C	2.84851700	-2.28678200	-0.26242900
C	3.56849100	-3.08563600	-1.15703600
C	3.14517100	-2.38363700	1.10341300
C	4.56971000	-3.94764100	-0.70709700
H	3.34235100	-3.03089300	-2.21971400
C	4.13917000	-3.24409000	1.55746600
H	2.59483200	-1.78129100	1.82064700
C	4.85689100	-4.02739100	0.65204100
H	5.11872700	-4.55818300	-1.41883900
H	4.35469100	-3.30672800	2.62086500
H	5.63230000	-4.70009200	1.00837400
C	-0.73851600	0.89697400	-1.14847900
O	-0.33979700	0.23604300	-2.10402800
H	-2.42836900	-0.85269700	-1.99384200
C	-4.20138200	-0.69780700	-0.72334300
C	-4.99938700	-1.46131000	-1.58517000
C	-4.70991100	-0.37003400	0.54207100
C	-6.27859400	-1.86231900	-1.20943500
H	-4.61165100	-1.73539700	-2.56343900
C	-5.98556200	-0.77154000	0.92000500
H	-4.09060200	0.17758100	1.24684500
C	-6.77697200	-1.51595200	0.04422800
H	-6.88436100	-2.44814200	-1.89489000
H	-6.36182400	-0.51284100	1.90592500
H	-7.77250700	-1.83167500	0.34293400
C	-0.48871100	2.60720400	0.56341500
C	-1.40482300	3.76724800	0.19039400
H	-1.01157700	1.88473500	1.19739600
H	-0.83296400	4.47700300	-0.41411100
H	-2.22815800	3.41143100	-0.44061600
O	1.56056300	4.06529700	-0.23601600
C	2.22721100	5.25865700	0.10414800
H	1.46840700	6.04158000	0.05806800
H	2.64127600	5.21598600	1.12005600
H	3.02768600	5.48882800	-0.61021100
H	0.33058500	2.97609600	1.17373800
C	-1.96540600	4.44966800	1.43676400
H	-2.55137700	3.75019400	2.04637100
H	-1.15908600	4.84399800	2.06758000
H	-2.61995400	5.28542600	1.16960400

173°

at wb97xd/6-31+G*
Energy = -1745.446392 A.U.
Thermal correction to Gibbs Free Energy =

0.475394 A.U.
Sum of electronic and thermal Free Energies =
-1744.970998 A.U.

P	1.52614900	-1.21045600	-1.00904000
C	-2.12261200	0.68888400	-0.63992500
C	2.21050800	0.50197600	-0.69545600
H	-2.52980700	1.29730900	0.15507700
C	-2.85703500	-0.30294500	-1.16019000
C	1.50516100	1.73946700	-0.63899900
C	3.60935900	0.52439600	-0.52888300
N	0.06944900	1.90025300	-0.63110000
C	2.24663400	2.88609300	-0.22868700
C	4.30358400	1.66103500	-0.16902000
H	4.16778100	-0.39718500	-0.63209200
C	3.61356500	2.85137100	0.00767700
H	5.37780400	1.62153400	-0.01600000
H	4.14651100	3.74958200	0.29450700
C	0.27220000	-1.39932800	0.33455700
C	-0.68027900	-2.41237800	0.17596700
C	0.20265800	-0.59319900	1.47291500
C	-1.67897600	-2.61094600	1.12428300
H	-0.65146800	-3.03639500	-0.71429400
C	-0.80530600	-0.77891900	2.41942800
H	0.93357900	0.19882800	1.61949500
C	-1.74949200	-1.78735100	2.24659700
H	-2.41863400	-3.39272500	0.97608300
H	-0.84735300	-0.13464400	3.29445600
H	-2.53963800	-1.93015600	2.97860000
C	2.84983700	-2.28525300	-0.26428300
C	3.57343300	-3.08043900	-1.15925700
C	3.14259700	-2.38607200	1.10212700
C	4.57447500	-3.94252300	-0.70911500
H	3.35023300	-3.02275700	-2.22241100
C	4.13634700	-3.24672100	1.55638800
H	2.58937800	-1.78666200	1.81962000
C	4.85776600	-4.02620600	0.65061700
H	5.12633800	-4.55015200	-1.42115100
H	4.34878100	-3.31247500	2.62022200
H	5.63297400	-4.69906000	1.00710300
C	-0.73864300	0.89461400	-1.15159300
O	-0.33923600	0.22845500	-2.10325600
H	-2.42187700	-0.86837300	-1.98229600
C	-4.19829100	-0.70452100	-0.71770800
C	-4.99324900	-1.47703400	-1.57429000
C	-4.70975700	-0.36659600	0.54382900
C	-6.27246300	-1.87698500	-1.19745700
H	-4.60314100	-1.75904700	-2.54935600
C	-5.98542400	-0.76702000	0.92287300
H	-4.09267400	0.18817000	1.24496900
C	-6.77384900	-1.52047400	0.05216400
H	-6.87590200	-2.46987900	-1.87887100
H	-6.36399200	-0.50040100	1.90579500
H	-7.76938700	-1.83534700	0.35175300
C	-0.49405100	2.61353300	0.54915600
C	-1.40885900	3.77233500	0.16961700
H	-1.01894300	1.89221500	1.18259000
H	-0.83436600	4.48099700	-0.43373200
H	-2.22906900	3.41488400	-0.46456700
O	1.56076300	4.06735400	-0.23347000
C	2.22606200	5.25854600	0.11663400
H	1.46915500	6.04295300	0.06489700
H	2.62969400	5.21250800	1.13660400
H	3.03420000	5.48918700	-0.58890300
H	0.32217900	2.98398000	1.16229400
C	-1.97549400	4.45724000	1.41186800
H	-2.56424900	3.75891800	2.02011900
H	-1.17225300	4.85297800	2.04572900
H	-2.62888600	5.29234400	1.13988500

174°

at wb97xd/6-31+G*
Energy = -1745.4473560000001 A.U.
Thermal correction to Gibbs Free Energy =
0.476566 A.U.
Sum of electronic and thermal Free Energies =
-1744.97079 A.U.

P	-0.10829600	0.89992200	-0.56770400
C	0.89497800	-1.84051800	-0.42558200
C	-1.20024400	0.23713100	0.78488100
H	0.88265800	-1.77186700	0.65881900
C	2.03591000	-1.89907000	-1.12368700
C	-1.82614600	-1.03854100	0.74355300
C	-1.60329500	1.19129600	1.73736100
N	-1.51957400	-2.05022000	-0.22644400
C	-3.01830900	-1.16862800	1.52315400
C	-2.71236300	1.00405700	2.53269100
H	-1.07929600	2.14023900	1.78469600
C	-3.43983500	-0.17142400	2.39440100
H	-3.03253700	1.76924900	3.23304800
H	-4.32985900	-0.31906900	2.99358100
C	1.51221800	1.19204800	0.25435200
C	2.58445700	1.52831800	-0.58129200
C	1.74915600	1.06300000	1.62569200
C	3.85516200	1.75080900	-0.05976900
H	2.42488900	1.60302100	-1.65501400
C	3.02283000	1.28056000	2.14917500
H	0.94027000	0.78291800	2.29436100
C	4.07759900	1.62816000	1.30999000
H	4.67648500	1.99815500	-0.72608800
H	3.18998100	1.16988900	3.21737600
H	5.07228500	1.78445700	1.71727300
C	-0.77950100	2.61259700	-0.71193400
C	-2.01786500	2.74825700	-1.35383400
C	-0.11717000	3.76487300	-0.27931700
C	-2.59262500	4.00161200	-1.53463600
H	-2.53941800	1.86137700	-1.70895500
C	-0.68629000	5.02357000	-0.47385300
H	0.84560000	3.68464800	0.21794300
C	-1.92557000	5.14574500	-1.09615500
H	-3.55753600	4.08739500	-2.02679700
H	-0.15830300	5.90977000	-0.13193000

H	-2.36853100	6.12664700	-1.24430900
C	-0.42047900	-1.91574200	-1.11721900
O	-0.52141400	-2.09779700	-2.31667800
H	1.94556300	-1.94760100	-2.20823900
C	3.39899100	-1.84343400	-0.58565600
C	4.46495900	-1.65794300	-1.47694800
C	3.68460000	-1.93460200	0.78365100
C	5.77317700	-1.54550500	-1.01733700
H	4.25869500	-1.59012600	-2.54261000
C	4.99056800	-1.82721900	1.24424100
H	2.88067500	-2.08813300	1.49703100
C	6.04001800	-1.62754300	0.34754300
H	6.58415200	-1.39639200	-1.72477300
H	5.19161500	-1.89580900	2.30974400
H	7.06033500	-1.54341900	0.71126900
C	-2.67641600	-2.63288000	-0.97226100
C	-3.56974000	-1.59933300	-1.65478100
H	-2.24091000	-3.27908400	-1.73271000
H	-4.01423700	-0.92767400	-0.90951700
H	-2.95342300	-0.98392700	-2.32009300
O	-3.64496800	-2.37768400	1.50435100
C	-4.87465600	-2.53672600	2.17487900
H	-5.22037500	-3.53943600	1.91944400
H	-4.75191400	-2.46565400	3.26312500
H	-5.61458100	-1.80028500	1.83652100
H	-3.25100600	-3.25410800	-0.29407900
C	-4.68140900	-2.28048900	-2.45124300
H	-4.26553000	-2.92656100	-3.23299900
H	-5.30897500	-2.90280000	-1.80082900
H	-5.33054200	-1.54388000	-2.93637400

175°

at wb97xd/6-31+G*
Energy = -1745.448339 A.U.
Thermal correction to Gibbs Free Energy = 0.476615 A.U.
Sum of electronic and thermal Free Energies = -1744.971724 A.U.

P	-0.11982300	0.90002700	-0.56599600
C	0.90234800	-1.83860000	-0.43743300
C	-1.20138600	0.21968300	0.78613000
H	0.88747600	-1.77830200	0.64743600
C	2.04499400	-1.88353400	-1.13364700
C	-1.81511800	-1.06095200	0.73780800
C	-1.60839400	1.16177200	1.74888100
N	-1.51201300	-2.05918800	-0.24776500
C	-2.99917600	-1.21210800	1.52539300
C	-2.71031900	0.95557800	2.54959400
H	-1.09436100	2.11592000	1.80086700
C	-3.42575900	-0.22705400	2.40789500
H	-3.03441700	1.71180100	3.25787100
H	-4.30999200	-0.38870800	3.01196700
C	1.50155000	1.19803300	0.25223000
C	2.56875100	1.54484800	-0.58551000
C	1.74406800	1.06404000	1.62212500
C	3.83992600	1.77257200	-0.06733800
H	2.40495800	1.62361400	-1.65830600
C	3.01817600	1.28696000	2.14219800
H	0.93937900	0.77576100	2.29234500
C	4.06792200	1.64479500	1.30101000
H	4.65741500	2.02800300	-0.73532900
H	3.18976800	1.17217700	3.20925800
H	5.06308600	1.80504800	1.70556200
C	-0.80492800	2.60842900	-0.69402700
C	-2.04827300	2.73921100	-1.32727700
C	-0.14877300	3.76251700	-0.25690700
C	-2.63371400	3.98939700	-1.49523700
H	-2.56531000	1.85094000	-1.68553200
C	-0.72873100	5.01819700	-0.43858500
H	0.81763900	3.68593800	0.23383400
C	-1.97274100	5.13537300	-1.05234800
H	-3.60231600	4.07138200	-1.98075400
H	-0.20548300	5.90590400	-0.09331000
H	-2.42414400	6.11389200	-1.19045800
C	-0.41101900	-1.91634100	-1.13322100
O	-0.50692200	-2.09182400	-2.33428700
H	1.95683300	-1.92420400	-2.21870600
C	3.40685300	-1.82306000	-0.59294500
C	4.47323800	-1.62654500	-1.48137300
C	3.69081900	-1.92067300	0.77624500
C	5.78012000	-1.50990000	-1.01903400
H	4.26828400	-1.55351100	-2.54694200
C	4.99549400	-1.80919400	1.23956500
H	2.88658000	-2.08249200	1.48744400
C	6.04527800	-1.59867400	0.34575500
H	6.59138800	-1.35226400	-1.72428400
H	5.19523400	-1.88304400	2.30496800
H	7.06456400	-1.51131000	0.71160600
C	-2.67503700	-2.61106700	-1.00727300
C	-3.54517400	-1.55015200	-1.67809700
H	-2.24771800	-3.25474900	-1.77448400
H	-3.98712800	-0.88741500	-0.92322700
H	-2.91261500	-0.93073300	-2.32426700
O	-3.61003800	-2.42899100	1.49917000
C	-4.81487400	-2.62043500	2.20552500
H	-5.14496800	-3.62922800	1.95365700
H	-4.66017100	-2.55293900	3.28991400
H	-5.58180600	-1.89953800	1.89503900
H	-3.26495700	-3.23008700	-0.34036600
C	-4.65833400	-2.19721700	-2.50047400
H	-4.24351400	-2.83154900	-3.29231100
H	-5.30152100	-2.82467900	-1.87064000
H	-5.29180200	-1.44057900	-2.97540200

176°

at wb97xd/6-31+G*
Energy = -1745.4492819999998 A.U.
Thermal correction to Gibbs Free Energy = 0.476528 A.U.
Sum of electronic and thermal Free Energies = -1744.972754 A.U.

P	-0.13038300	0.90102700	-0.56369900
C	0.90786200	-1.83717300	-0.45036300
C	-1.20405800	0.20437300	0.78636300
H	0.89087500	-1.78576400	0.63492400
C	2.05186400	-1.86923600	-1.14499000
C	-1.80568900	-1.08105000	0.73118300
C	-1.61596600	1.13497200	1.75817700
N	-1.50631600	-2.06620400	-0.26908500
C	-2.98139300	-1.25311800	1.52650000
C	-2.71090800	0.91023500	2.56362900
H	-1.11204800	2.09424500	1.81473700
C	-3.41366000	-0.27984600	2.41916900
H	-3.03941800	1.65787900	3.27896500
H	-4.29199600	-0.45545700	3.02790800
C	1.49111300	1.20238800	0.25313600
C	2.55505700	1.55791600	-0.58509500
C	1.73683400	1.06365200	1.62197300
C	3.82609500	1.78953500	-0.06826800
H	2.38887300	1.64041100	-1.65723600
C	3.01083100	1.29042600	2.14062600
H	0.93486300	0.76864500	2.29249900
C	4.05729400	1.65690200	1.29906500
H	4.64112800	2.05169400	-0.73665500
H	3.18497700	1.17175400	3.20684500
H	5.05244900	1.82004600	1.70246400
C	-0.82602200	2.60619600	-0.67655500
C	-2.07218100	2.73456300	-1.30481700
C	-0.17498200	3.76070500	-0.23301700
C	-2.66511400	3.98262000	-1.46178200
H	-2.58540600	1.84606100	-1.66789900
C	-0.76261600	5.01440500	-0.40358400
H	0.79344500	3.68597400	0.25402600
C	-2.00921100	5.12905400	-1.01250700
H	-3.63568700	4.06272800	-1.94366400
H	-0.24323600	5.90247800	-0.05344200
H	-2.46655400	6.10598900	-1.14199400
C	-0.40367800	-1.91615100	-1.14970900
O	-0.49544200	-2.08491300	-2.35225200
H	1.96545600	-1.90185700	-2.23046800
C	3.41266600	-1.80507200	-0.60201300
C	4.47943300	-1.59989100	-1.48801000
C	3.69518500	-1.90755900	0.76711900
C	5.78519200	-1.47988700	-1.02335100
H	4.27563600	-1.52283100	-2.55351700
C	4.99874100	-1.79275600	1.23275400
H	2.89064000	-2.07552700	1.47653500
C	6.04886700	-1.57380500	0.34137600
H	6.59674600	-1.31559300	-1.72675100
H	5.19731000	-1.87049800	2.29810300
H	7.06727500	-1.48381400	0.70904000
C	-2.67526600	-2.58850300	-1.04018000
C	-3.52245900	-1.50249400	-1.70063300
H	-2.25655900	-3.23110100	-1.81307400
H	-3.95838000	-0.84471900	-0.93779700
H	-2.87501500	-0.88369300	-2.33254600
O	-3.57553400	-2.47803200	1.49388200
C	-4.75570200	-2.70114400	2.23200000
H	-5.06865300	-3.71624800	1.98386600
H	-4.57232500	-2.63471500	3.31197700
H	-5.54796800	-1.99747400	1.94668100
H	-3.27993200	-3.20375100	-0.38305700
C	-4.64024600	-2.11720700	-2.54135000
H	-4.23003500	-2.74448500	-3.34114600
H	-5.29801300	-2.74478800	-1.92691700
H	-5.25799500	-1.34230400	-3.00746300

177°

at wb97xd/6-31+G*
Energy = -1745.450274 A.U.
Thermal correction to Gibbs Free Energy = 0.476263 A.U.
Sum of electronic and thermal Free Energies = -1744.974011 A.U.

P	-0.14134200	0.90184500	-0.56107900
C	0.91435700	-1.83573800	-0.46346500
C	-1.20588200	0.18826000	0.78733300
H	0.89558800	-1.79367800	0.62219300
C	2.05957400	-1.85388800	-1.15651800
C	-1.79509000	-1.10188000	0.72476000
C	-1.62230600	1.10659800	1.76879500
N	-1.49952300	-2.07344900	-0.29028300
C	-2.96211900	-1.29526600	1.52732800
C	-2.70990400	0.86278100	2.57885000
H	-1.12863800	2.07089600	1.83031100
C	-3.39983200	-0.33454300	2.43080500
H	-3.04260200	1.60130600	3.30171300
H	-4.27195600	-0.52471100	3.04409700
C	1.48048500	1.20750400	0.25347100
C	2.54014900	1.57380500	-0.58551300
C	1.73068100	1.06208700	1.62079900
C	3.81133600	1.80924000	-0.07074100
H	2.37058100	1.66172400	-1.65669300
C	3.00485500	1.29259000	2.13730400
H	0.93220600	0.75879200	2.29178000
C	4.04703800	1.66966900	1.29511500
H	4.62309200	2.07985800	-0.73975100
H	3.18260500	1.16838300	3.20229500
H	5.04243200	1.83561700	1.69676100
C	-0.84878000	2.60307600	-0.65842000
C	-2.09761000	2.72806300	-1.28207200
C	-0.20427300	3.75830600	-0.20729200
C	-2.69937300	3.97333600	-1.42718400
H	-2.60586700	1.83910700	-1.65096200
C	-0.80084200	5.00934700	-0.36595200
H	0.76609300	3.68610600	0.27627300
C	-2.04993600	5.12048100	-0.97039700
H	-3.67183100	4.05080700	-1.90569000
H	-0.28646200	5.89804800	-0.01007300
H	-2.51422400	6.09532100	-1.09063800
C	-0.39546800	-1.91628200	-1.16614500
O	-0.48342300	-2.07837900	-2.37009400

H	1.97483900	-1.87782700	-2.24236100
C	3.41926500	-1.78547100	-0.61117600
C	4.48612000	-1.56875900	-1.49431200
C	3.70054200	-1.89544600	0.75762800
C	5.79063600	-1.44513100	-1.02713500
H	4.28330600	-1.48565500	-2.55955000
C	5.00289500	-1.77707400	1.22577400
H	2.89598000	-2.07231800	1.46485500
C	6.05303200	-1.54680100	0.33728300
H	6.60224400	-1.27195900	-1.72834000
H	5.20048200	-1.86091500	2.29084800
H	7.07048800	-1.45404300	0.70689600
C	-2.67444400	-2.56564500	-1.07193900
C	-3.49768000	-1.45499500	-1.72219100
H	-2.26499600	-3.20785200	-1.85019600
H	-3.92530200	-0.80023100	-0.95194000
H	-2.83550700	-0.83960600	-2.34209600
O	-3.53989300	-2.52773100	1.48706500
C	-4.69874000	-2.78013100	2.24929800
H	-4.99611300	-3.79977600	2.00083100
H	-4.49258900	-2.71681400	3.32534800
H	-5.51159100	-2.09127600	1.98665500
H	-3.29386500	-3.17578900	-0.42373800
C	-4.62188700	-2.03691800	-2.57754000
H	-4.21860700	-2.65981000	-3.38424200
H	-5.29442600	-2.66125800	-1.97595400
H	-5.22281700	-1.24414600	-3.03553700

178°

at wb97xd/6-31+G*
Energy = -1745.451268 A.U.
Thermal correction to Gibbs Free Energy = 0.476439 A.U.
Sum of electronic and thermal Free Energies = -1744.974829 A.U.

P	-0.15158200	0.90309700	-0.55888100
C	0.91957400	-1.83477300	-0.47616300
C	-1.20844200	0.17433300	0.78739600
H	0.89941600	-1.80103200	0.60975700
C	2.06574600	-1.84073300	-1.16779200
C	-1.78572700	-1.12021800	0.71817000
C	-1.62949000	1.08121900	1.77746000
N	-1.49408300	-2.07924400	-0.31013800
C	-2.94386900	-1.33405200	1.52780200
C	-2.70974800	0.81925500	2.59191800
H	-1.14599100	2.05037800	1.84334900
C	-3.38685300	-0.38519800	2.44109700
H	-3.04651500	1.54926000	3.32154600
H	-4.25265700	-0.58946100	3.05884500
C	1.47032100	1.21153100	0.25450100
C	2.52681300	1.58664900	-0.58453400
C	1.72349000	1.06091000	1.62072200
C	3.79779000	1.82553800	-0.07079000
H	2.35501000	1.67872400	-1.65500800
C	2.99746900	1.29477000	2.13613000
H	0.92761100	0.75082600	2.29166500
C	4.03647200	1.68062400	1.29397000
H	4.60713900	2.10307100	-0.73988100
H	3.17764600	1.16627200	3.20020100
H	5.03178800	1.84920100	1.69470600
C	-0.86839200	2.60112600	-0.64171400
C	-2.11932200	2.72426900	-1.26153700
C	-0.22893100	3.75625900	-0.18323200
C	-2.72793400	3.96742200	-1.39579200
H	-2.62372800	1.83550200	-1.63605600
C	-0.83244000	5.00532100	-0.33097900
H	0.74294400	3.68548800	0.29750000
C	-2.08348000	5.11446800	-0.93173000
H	-3.70187200	4.04341000	-1.87152000
H	-0.32190700	5.89398300	0.03048900
H	-2.55315100	6.08773500	-1.04350700
C	-0.38876100	-1.91656400	-1.18165700
O	-0.47357500	-2.07296800	-2.38677800
H	1.98246000	-1.85707100	-2.25389100
C	3.42440600	-1.76853600	-0.62026500
C	4.49123300	-1.54092800	-1.50068100
C	3.70462100	-1.88567500	0.74815700
C	5.79456600	-1.41374400	-1.03115500
H	4.28928400	-1.45219500	-2.56562900
C	5.00581200	-1.76375100	1.21864200
H	2.90018900	-2.07124100	1.45329500
C	6.05584500	-1.52263100	0.33291600
H	6.60613700	-1.23216300	-1.73027300
H	5.20254400	-1.85334500	2.28340800
H	7.07237500	-1.42706800	0.70436100
C	-2.67458200	-2.54376000	-1.10014400
C	-3.47474800	-1.41136000	-1.74217200
H	-2.27430300	-3.18690900	-1.88252100
H	-3.89339700	-0.75761100	-0.96599800
H	-2.79905900	-0.80143400	-2.35282100
O	-3.50552800	-2.57367500	1.48067500
C	-4.64485600	-2.85289300	2.26285400
H	-4.92723000	-3.87655400	2.01357900
H	-4.41948200	-2.79173300	3.33516600
H	-5.47571200	-2.17842900	2.01993200
H	-3.30765600	-3.14740600	-0.45884700
C	-4.60566300	-1.96342800	-2.60837900
H	-4.21009100	-2.58438500	-3.42036000
H	-5.29191600	-2.58223600	-2.01666500
H	-5.19028000	-1.15490600	-3.05992500

179°

at wb97xd/6-31+G*
Energy = -1745.452264 A.U.
Thermal correction to Gibbs Free Energy = 0.473727 A.U.
Sum of electronic and thermal Free Energies = -1744.978537 A.U.

P	-0.16098700	0.90473500	-0.55740900
C	0.92405100	-1.83398600	-0.48718300

C	-1.21076000	0.16261600	0.78704700
H	0.90248700	-1.80765300	0.59891300
C	2.07105200	-1.82871600	-1.17738800
C	-1.77729100	-1.13579000	0.71186400
C	-1.63549600	1.05889800	1.78510300
N	-1.48952400	-2.08360500	-0.32804400
C	-2.92648000	-1.36850300	1.52842700
C	-2.70834500	0.78023000	2.60406700
H	-1.16125300	2.03235100	1.85494200
C	-3.37361500	-0.43067600	2.45096500
H	-3.04835000	1.50229700	3.34009300
H	-4.23302900	-0.64801500	3.07322500
C	1.46109700	1.21553100	0.25473000
C	2.51448500	1.59885600	-0.58447700
C	1.71735700	1.05966200	1.61977500
C	3.78542800	1.84053400	-0.07191400
H	2.34034100	1.69522200	-1.65419100
C	2.99132400	1.29624200	2.13392700
H	0.92397900	0.74334300	2.29075800
C	4.02722000	1.69022500	1.29170300
H	4.59238800	2.12459500	-0.74115500
H	3.17401300	1.16343800	3.19704100
H	5.02258800	1.86088100	1.69141600
C	-0.88580400	2.59996500	-0.62721100
C	-2.13750500	2.72200700	-1.24567600
C	-0.25182100	3.75450300	-0.15965500
C	-2.75220800	3.96324500	-1.36977200
H	-2.63763200	1.83389500	-1.62736400
C	-0.86144100	5.00175100	-0.29717000
H	0.72063400	3.68463700	0.32004200
C	-2.11322200	5.10962500	-0.89664200
H	-3.72662100	4.03830700	-1.84467200
H	-0.35509000	5.88995500	0.07123700
H	-2.58764400	6.08146500	-1.00048300
C	-0.38283600	-1.91717100	-1.19535100
O	-0.46467600	-2.06942600	-2.40142300
H	1.98896200	-1.83816400	-2.26367000
C	3.42880100	-1.75320000	-0.62799400
C	4.49561100	-1.51647900	-1.50601500
C	3.70808500	-1.87644000	0.74008000
C	5.79793500	-1.38662200	-1.03445300
H	4.29438700	-1.42280200	-2.57067700
C	5.00829700	-1.75188000	1.21260800
H	2.90365700	-2.06896500	1.44335400
C	6.05825100	-1.50181700	0.32928800
H	6.60949700	-1.19800200	-1.73171400
H	5.20430200	-1.84644300	2.27708000
H	7.07402100	-1.40425100	0.70229600
C	-2.67474500	-2.52449800	-1.12423300
C	-3.45495400	-1.37434500	-1.75967100
H	-2.28272300	-3.16901800	-1.90981400
H	-3.86338300	-0.71947500	-0.97890200
H	-2.76849300	-0.77152900	-2.36530400
O	-3.47367300	-2.61426800	1.47478400
C	-4.59714600	-2.91614600	2.27148200
H	-4.86719400	-3.94248500	2.01970900
H	-4.35705300	-2.85795400	3.34075700
H	-5.44186500	-2.25327000	2.04499700
H	-3.31922300	-3.12180500	-0.48808800
C	-4.59377700	-1.90121000	-2.63118300
H	-4.20744600	-2.52301700	-3.44694800
H	-5.29134500	-2.51246100	-2.04488900
H	-5.16394100	-1.07978000	-3.07790800

-10°

at wb97xd/6-31+G*
Energy = -1745.462185 A.U.
Thermal correction to Gibbs Free Energy = 0.473598 A.U.
Sum of electronic and thermal Free Energies = -1744.988587 A.U.

P	2.27326300	-0.03092900	-0.81455300
C	-2.20678100	0.84131900	0.01105500
C	1.81987000	0.74133700	0.81589700
H	-1.66015000	0.04009000	0.50005400
C	-3.49731900	0.70598300	-0.30983100
C	0.63197000	1.51405700	0.84600100
C	2.46012100	0.40766800	2.00802800
N	-0.02106400	1.78123000	-0.40017400
C	-0.00464900	1.67488800	2.08721000
C	1.87399900	0.69833600	3.23907500
H	3.38639100	-0.15776000	1.98271300
C	0.62522900	1.29874500	3.27283700
H	2.36964000	0.41025500	4.16145600
H	0.10902900	1.48970300	4.20909900
C	0.84890400	-1.20668100	-0.88040700
C	0.16826500	-1.34639500	-2.09202500
C	0.35436900	-1.87434000	0.24860800
C	-0.99058100	-2.11853900	-2.17524900
H	0.52918800	-0.82350100	-2.97397900
C	-0.79515900	-2.65373700	0.16495700
H	0.85790400	-1.76447200	1.20617200
C	-1.47871000	-2.76758400	-1.04560500
H	-1.52163100	-2.19476800	-3.11971900
H	-1.17297300	-3.15680400	1.05104000
H	-2.39767000	-3.34445500	-1.09859300
C	3.70854400	-1.07912700	-0.33958200
C	4.95136300	-0.43618000	-0.24765800
C	3.65295400	-2.45957000	-0.12571400
C	6.10132700	-1.14612800	0.08325000
H	5.01869500	0.63394800	-0.43435400
C	4.80807300	-3.17532500	0.18854300
H	2.70693400	-2.98577500	-0.20854800
C	6.03210000	-2.52168200	0.30170800
H	7.05323500	-0.62789200	0.15890600
H	4.74704600	-4.24852200	0.34720200
H	6.92959900	-3.08093500	0.55046200
C	-1.41883000	2.01976400	-0.41966700
O	-1.90294400	3.01432600	-0.93038900
H	-3.97560000	1.50973600	-0.86834000
C	-4.30844700	-0.48574300	-0.01858400
C	-5.40788300	-0.78604800	-0.83267500

C	-4.00535400	-1.35262900	1.04096300
C	-6.16277100	-1.93646800	-0.61955600
H	-5.66257300	-0.11584200	-1.65041000
C	-4.76137700	-2.49871900	1.25887100
H	-3.17918500	-1.11743000	1.70588100
C	-5.83903300	-2.79961100	0.42523800
H	-7.00617700	-2.15709000	-1.26783100
H	-4.51564300	-3.15642900	2.08836300
H	-6.42966500	-3.69492600	0.59776900
C	0.71132000	2.78936000	-1.21796700
C	0.54175500	2.56859300	-2.71620800
H	0.36768500	3.79522100	-0.94553500
H	0.90417300	1.56538500	-2.96548300
H	-0.51954700	2.61499700	-2.97817500
O	-1.23029200	2.28653100	2.16789600
C	-1.17166700	3.70429700	2.27751600
H	-2.20514500	4.05093400	2.30820400
H	-0.64555900	3.99904200	3.19446200
H	-0.66931400	4.14148100	1.40598400
H	1.76923500	2.71746500	-0.95793700
C	1.31943800	3.61332700	-3.51467200
H	0.96747700	4.62682800	-3.28825500
H	2.39139500	3.56862900	-3.28607500
H	1.20136600	3.45235500	-4.59121000

-30°

at wb97xd/6-31+G*
Energy = -1745.473627 A.U.
Thermal correction to Gibbs Free Energy = 0.473968 A.U.
Sum of electronic and thermal Free Energies = -1744.999659 A.U.

P	2.18984000	-0.10892000	-0.78542900
C	-2.11020900	0.88173400	0.02158500
C	1.73261100	0.63907200	0.85093500
H	-1.56815400	0.13824300	0.59782800
C	-3.38376100	0.68507500	-0.33297400
C	0.63430600	1.52424400	0.85321800
C	2.30661400	0.25429100	2.06272900
N	0.04585500	1.90998200	-0.39181100
C	0.03653300	1.85590500	2.07935500
C	1.75020400	0.65830600	3.27498400
H	3.16201700	-0.41416700	2.06620300
C	0.60071700	1.43605600	3.28208500
H	2.19458500	0.33363600	4.21141900
H	0.11709800	1.72988600	4.20896800
C	0.71617700	-1.21012400	-0.93509200
C	0.02428200	-1.22520100	-2.14803300
C	0.20192300	-1.94506900	0.14234200
C	-1.16345700	-1.94547400	-2.28166600
H	0.39916800	-0.64580400	-2.98782400
C	-0.97512200	-2.67311500	0.00700500
H	0.71513700	-1.92907800	1.10106200
C	-1.66779000	-2.66560400	-1.20386200
H	-1.70327800	-1.92521300	-3.22408800
H	-1.37018500	-3.22761500	0.85359400
H	-2.60898500	-3.20109200	-1.29317900
C	3.55900900	-1.24161000	-0.30726300
C	4.81273600	-0.65504200	-0.08040600
C	3.45004800	-2.63322900	-0.23140000
C	5.91792400	-1.43329300	0.24825700
H	4.92397400	0.42488700	-0.15805100
C	4.56159500	-3.41606500	0.08118500
H	2.49650000	-3.11626100	-0.42131900
C	5.79475900	-2.82022400	0.32941100
H	6.87808200	-0.95835400	0.42988100
H	4.45851500	-4.49654200	0.13195200
H	6.65796700	-3.43168300	0.57659700
C	-1.34588300	2.04320300	-0.49452400
O	-1.85680300	2.96362200	-1.11454900
H	-3.85709800	1.42443000	-0.97780500
C	-4.17073400	-0.50527800	0.02191800
C	-5.18465000	-0.94019900	-0.84063900
C	-3.91440000	-1.24947400	1.18154200
C	-5.89583800	-2.10769400	-0.57628500
H	-5.40258300	-0.36485700	-1.73740600
C	-4.62866200	-2.41130700	1.45131600
H	-3.15546600	-0.90781800	1.87993900
C	-5.61630900	-2.85054100	0.56913600
H	-6.67096900	-2.43567500	-1.26330400
H	-4.41987600	-2.97400700	2.35727300
H	-6.17337500	-3.75880100	0.78114100
C	0.81208400	2.92370500	-1.15220100
C	0.76151000	2.71283400	-2.66227300
H	0.43095300	3.92435000	-0.90881100
H	1.17051100	1.72342700	-2.89245500
H	-0.27913400	2.73372800	-2.99790600
O	-1.11132500	2.60267800	2.11505600
C	-0.90837600	4.01035200	2.09304900
H	-1.90073500	4.46170600	2.11370700
H	-0.32944300	4.33018400	2.96894600
H	-0.39104100	4.31764000	1.17700000
H	1.84846200	2.86698000	-0.80834200
C	1.56500300	3.78897400	-3.39068500
H	1.16470500	4.78902600	-3.18540400
H	2.61765400	3.77659400	-3.08189200
H	1.53436500	3.63452800	-4.47416700

-50°

at wb97xd/6-31+G*
Energy = -1745.482320 A.U.
Thermal correction to Gibbs Free Energy = 0.47355 A.U.
Sum of electronic and thermal Free Energies = -1745.00877 A.U.

P	-2.22282800	-0.08349300	0.71184500
C	1.96800700	0.80042200	0.15852900
C	-1.60440400	0.59474500	-0.89914800
H	1.42031300	0.12576500	-0.49092000
C	3.23841500	0.55678700	0.49580900

C	-0.59331300	1.55961800	-0.81708100
C	-2.03227400	0.15378700	-2.15628700
N	-0.12403600	2.00547400	0.45962500
C	0.02954100	2.02884200	-1.98979300
C	-1.42498700	0.63690700	-3.30858700
H	-2.81870400	-0.58988500	-2.23452700
C	-0.38766800	1.56380100	-3.23662200
H	-1.75182700	0.27980800	-4.28125200
H	0.07903700	1.92236800	-4.14729500
C	-0.77602400	-1.15756600	1.09979300
C	-0.19935800	-1.05053800	2.36698500
C	-0.17713600	-2.00121400	0.15406400
C	0.95703700	-1.76420300	2.68391600
H	-0.63957300	-0.38202100	3.10246900
C	0.96804800	-2.72260600	0.47222200
H	-0.60225300	-2.07960500	-0.84417700
C	1.54300200	-2.59804300	1.73722000
H	1.40685100	-1.65252700	3.66631500
H	1.43008400	-3.36477800	-0.27216600
H	2.45603000	-3.13777600	1.97214500
C	-3.52011800	-1.26345600	0.15030500
C	-4.72071400	-0.70917100	-0.31849400
C	-3.42414700	-2.65448300	0.25118400
C	-5.77983300	-1.52133400	-0.70981200
H	-4.82568100	0.37223400	-0.38291200
C	-4.49207100	-3.46963900	-0.12529500
H	-2.51548400	-3.11221000	0.63000400
C	-5.66823400	-2.90840500	-0.61350000
H	-6.69696500	-1.07136300	-1.07982800
H	-4.39897900	-4.54859900	-0.03640700
H	-6.49699100	-3.54527400	-0.90962200
C	1.23151400	1.94553400	0.75578900
O	1.75190300	2.72616200	1.54157200
H	3.71233700	1.23643400	1.20313300
C	4.03284100	-0.59158000	0.03663800
C	5.14473700	-0.99590900	0.78624300
C	3.70600200	-1.32066700	-1.11581400
C	5.89024100	-2.11281300	0.41738100
H	5.41679300	-0.43388000	1.67660800
C	4.45228400	-2.43181300	-1.48970900
H	2.86360600	-1.01109900	-1.72828600
C	5.54415800	-2.83722700	-0.72100700
H	6.74380700	-2.41489500	1.01778200
H	4.18581300	-2.98229900	-2.38798600
H	6.12668000	-3.70584800	-1.01475700
C	-0.86664300	3.13040200	1.06024200
C	-1.10998100	2.97635800	2.55989000
H	-0.31795700	4.06049100	0.86191400
H	-1.68038300	2.05700300	2.73145200
H	-0.14911500	2.87742600	3.07200700
O	1.00980400	2.94393100	-1.80657600
C	1.80478700	3.32166400	-2.91083200
H	2.57403000	3.98069100	-2.50827500
H	2.27817600	2.44661100	-3.37333100
H	1.21474700	3.86467400	-3.65984100
H	-1.82705000	3.18590900	0.53842900
C	-1.87550300	4.17721200	3.11384700
H	-1.31260800	5.10683100	2.96725900
H	-2.84905000	4.29214600	2.62091400
H	-2.05789200	4.06451800	4.18767500

-70°

at wb97xd/6-31+G*
Energy = -1745.484639 A.U.
Thermal correction to Gibbs Free Energy = 0.473738 A.U.
Sum of electronic and thermal Free Energies = -1745.010901 A.U.

P	-2.07797200	-0.29929700	0.72782000
C	1.86577300	0.86995900	0.18560500
C	-1.55374500	0.52166500	-0.84890100
H	1.39503000	0.35540000	-0.64433800
C	3.10082000	0.55084300	0.58633700
C	-0.69324300	1.61811000	-0.73366900
C	-1.96687200	0.11196800	-2.12301800
N	-0.23408400	2.06680900	0.54390100
C	-0.24806800	2.29819600	-1.88352700
C	-1.52261900	0.78690700	-3.25261600
H	-2.63080700	-0.73939300	-2.23225600
C	-0.66347700	1.87926900	-3.14694000
H	-1.84440400	0.45901900	-4.23724100
H	-0.32936400	2.38969800	-4.04324600
C	-0.55955800	-1.27703100	1.08159100
C	-0.04064800	-1.22808800	2.37703300
C	0.12329400	-2.01698400	0.10623900
C	1.13583000	-1.90698000	2.69608200
H	-0.54658400	-0.63627800	3.13574500
C	1.28858100	-2.70413300	0.42512600
H	-0.25687400	-2.04510200	-0.91277400
C	1.79891200	-2.64643300	1.72231100
H	1.53708100	-1.84576800	3.70367100
H	1.81691100	-3.26441400	-0.34083700
H	2.72347600	-3.16372700	1.96225700
C	-3.27646600	-1.55556800	0.11067200
C	-4.52913400	-1.08046200	-0.30624700
C	-3.05798500	-2.93624800	0.11873600
C	-5.52016600	-1.95573500	-0.73736800
H	-4.72872300	-0.01058400	-0.29848500
C	-4.05725800	-3.81654200	-0.29785400
H	-2.10651700	-3.33574300	0.45587900
C	-5.28629300	-3.33095900	-0.73394000
H	-6.47952000	-1.56544800	-1.06575300
H	-3.86877500	-4.88655500	-0.28128600
H	-6.06147800	-4.01801300	-1.06139000
C	1.08110600	1.89184000	0.92848000
O	1.55514900	2.52440100	1.86608100
H	3.49247300	1.06511600	1.46302400
C	3.95675500	-0.47749900	-0.02077200
C	5.01095700	-1.01254100	0.72995500
C	3.74350600	-0.97165300	-1.31551500
C	5.80848800	-2.03360300	0.22017600
H	5.19384600	-0.63206300	1.73215300

C	4.54211300	-1.98653800	-1.82942600
H	2.94906500	-0.55446000	-1.92815400
C	5.57395500	-2.52747400	-1.06117900
H	6.61542700	-2.44125600	0.82271200
H	4.36330300	-2.35523900	-2.83586300
H	6.19703400	-3.32077700	-1.46435500
C	-0.96516500	3.18074700	1.16986600
C	-1.42793400	2.89599400	2.59803400
H	-0.31733200	4.06594200	1.15901100
H	-2.10672400	2.03687400	2.59072900
H	-0.55672700	2.62664600	3.20228000
O	0.58667200	3.33891800	-1.65561200
C	1.14222600	4.01639500	-2.76344800
H	1.80145700	4.77661000	-2.34433300
H	1.72671100	3.33356200	-3.39263600
H	0.36321100	4.50042100	-3.36582800
H	-1.83082100	3.38780600	0.53255900
C	-2.12426300	4.11572000	3.19979200
H	-1.44678300	4.97698900	3.24199000
H	-3.00173400	4.40753600	2.60877700
H	-2.46481200	3.90960800	4.21993700

-80°

at wb97xd/6-31+G*
Energy = -1745.4847 A.U.
Thermal correction to Gibbs Free Energy = 0.473389 A.U.
Sum of electronic and thermal Free Energies = -1745.011311 A.U.

P	-1.97531400	-0.44165100	0.74247800
C	1.80679400	0.93544200	0.23492700
C	-1.53354700	0.46252000	-0.81470300
H	1.39362100	0.50909000	-0.67214000
C	3.03049500	0.60705100	0.66263100
C	-0.77097700	1.62833700	-0.68571500
C	-1.93798500	0.05450400	-2.09297700
N	-0.32122500	2.08863800	0.58941100
C	-0.43087500	2.38703900	-1.82290700
C	-1.59372600	0.80429300	-3.20965000
H	-2.52495400	-0.85012900	-2.21410100
C	-0.84328500	1.97270700	-3.08847700
H	-1.91148900	0.47836200	-4.19622000
H	-0.58745000	2.54173400	-3.97516000
C	-0.41950000	-1.38034600	1.02826400
C	0.09539900	-1.40224400	2.32597700
C	0.28630600	-2.03359200	0.00827800
C	1.28987300	-2.06752500	2.60404800
H	-0.42895400	-0.87710300	3.12036000
C	1.47014100	-2.70656200	0.28536300
H	-0.09176600	-2.00650000	-1.01148500
C	1.97523500	-2.72180100	1.58585300
H	1.68662600	-2.06313900	3.61527300
H	2.01623400	-3.19888900	-0.51412900
H	2.91308100	-3.22851100	1.79477300
C	-3.13868700	-1.72022500	0.10252600
C	-4.42230700	-1.28152800	-0.25584800
C	-2.86067600	-3.08845200	0.03589500
C	-5.38721200	-2.17812700	-0.70196300
H	-4.66683700	-0.22299000	-0.19073800
C	-3.83314300	-3.99127100	-0.39587700
H	-1.88279000	-3.46063100	0.32552700
C	-5.09444600	-3.54027100	-0.77288400
H	-6.37181600	-1.81548800	-0.98410600
H	-3.59832600	-5.05139500	-0.43776800
H	-5.84885300	-4.24449800	-1.11218700
C	0.96657600	1.86903700	1.03172700
O	1.38561700	2.42094600	2.04448300
H	3.36087600	1.03568300	1.60784400
C	3.95469300	-0.31984200	-0.00437600
C	5.01571000	-0.86314500	0.73062400
C	3.80329800	-0.71088000	-1.34232800
C	5.88104700	-1.79339500	0.16141800
H	5.15012700	-0.56253000	1.76704400
C	4.66884300	-1.63536500	-1.91503500
H	3.00390600	-0.28540000	-1.94254100
C	5.70776400	-2.18654400	-1.16378500
H	6.69254300	-2.20913900	0.75221600
H	4.53662700	-1.92508700	-2.95397300
H	6.38318500	-2.90907900	-1.61307900
C	-1.06560600	3.18727400	1.22323400
C	-1.69989200	2.81994900	2.56377500
H	-0.37452600	4.02694600	1.36012100
H	-2.43171300	2.01975700	2.41365300
H	-0.91855200	2.42899200	3.22301100
O	0.30942200	3.49413300	-1.58065000
C	0.73236200	4.27724700	-2.67744000
H	1.32227500	5.08871200	-2.25111000
H	1.35849000	3.69328900	-3.36358800
H	-0.12376900	4.69511900	-3.22190400
H	-1.83914100	3.49978700	0.51423400
C	-2.36634100	4.03627000	3.20472100
H	-1.63564000	4.83075100	3.39791800
H	-3.14918800	4.45101500	2.55684200
H	-2.83185100	3.77160700	4.15992100

-90°

at wb97xd/6-31+G*
Energy = -1745.48443 A.U.
Thermal correction to Gibbs Free Energy = 0.472246 A.U.
Sum of electronic and thermal Free Energies = -1745.012184 A.U.

P	-1.82200800	-0.61028200	0.75657600
C	1.74248100	1.03705000	0.31795700
C	-1.51010400	0.39625000	-0.77073300
H	1.39703900	0.70161000	-0.65375200
C	2.95848500	0.71932000	0.77507900
C	-0.86492800	1.62989400	-0.62189200
C	-1.91209400	-0.00742100	-2.05197700
N	-0.43223100	2.10320200	0.65196600

C	-0.64445300	2.45729200	-1.74202300
C	-1.68665400	0.81063600	-3.15006000
H	-2.41064000	-0.96172700	-2.18783900
C	-1.05697800	2.04625800	-3.00810500
H	-2.00430100	0.48741700	-4.13753300
H	-0.89121500	2.66851900	-3.88031100
C	-0.22639600	-1.51249300	0.90547400
C	0.31752700	-1.65429200	2.18381300
C	0.47664500	-2.03026500	-0.19092600
C	1.53810700	-2.30447800	2.36729800
H	-0.20486200	-1.23354000	3.03928500
C	1.68869800	-2.68552200	-0.00928700
H	0.07460700	-1.91116000	-1.19460500
C	2.22265100	-2.82153300	1.27236900
H	1.95632100	-2.39487200	3.36583100
H	2.23297200	-3.07024000	-0.86692700
H	3.18200800	-3.31244400	1.40848000
C	-2.97144100	-1.89444900	0.10163100
C	-4.29020900	-1.48933700	-0.15285500
C	-2.64167300	-3.24051400	-0.07939500
C	-5.24233400	-2.39515500	-0.60751400
H	-4.57269600	-0.44948100	0.00034900
C	-3.60023300	-4.15388800	-0.52028000
H	-1.63337800	-3.58708600	0.12586000
C	-4.89895000	-3.73469000	-0.79233100
H	-6.25572900	-2.05833000	-0.80790100
H	-3.32514500	-5.19679800	-0.65236700
H	-5.64273500	-4.44668200	-1.13883600
C	0.82494800	1.85445100	1.15680900
O	1.16748500	2.31345600	2.24256100
H	3.21659400	1.05733800	1.77778500
C	3.96771400	-0.07920400	0.06689900
C	5.03885800	-0.61256200	0.79444600
C	3.89132100	-0.35839100	-1.30493400
C	5.98788300	-1.42675600	0.18265900
H	5.11480800	-0.39822900	1.85786900
C	4.83972000	-1.16720200	-1.91972300
H	3.08432900	0.06185900	-1.89845900
C	5.88881700	-1.71127200	-1.17748600
H	6.80619000	-1.83712500	0.76781800
H	4.76412600	-1.37189400	-2.98426200
H	6.62906200	-2.34335700	-1.65986300
C	-1.23221300	3.14350700	1.31026800
C	-2.07340900	2.64073200	2.48155000
H	-0.54012400	3.91665100	1.65946200
H	-2.80363700	1.90749000	2.12305400
H	-1.41573900	2.11883000	3.18439000
O	-0.01091200	3.62572700	-1.48423600
C	0.26655900	4.49502400	-2.56228800
H	0.77879800	5.35269600	-2.12602200
H	0.92251800	4.01591500	-3.29992900
H	-0.65666000	4.83088400	-3.05108400
H	-1.87678100	3.58917400	0.54522900
C	-2.78453000	3.79757900	3.18150000
H	-2.06265200	4.51756700	3.58502600
H	-3.44508400	4.33700500	2.49063900
H	-3.39773300	3.43693500	4.01396000

-100°

at wb97xd/6-31+G*
Energy = -1745.48396 A.U.
Thermal correction to Gibbs Free Energy = 0.472618 A.U.
Sum of electronic and thermal Free Energies = -1745.011342 A.U.

P	-1.57307100	0.80520900	-0.75817700
C	1.64849200	-1.21339100	-0.42950900
C	-1.47180200	-0.31303400	0.72081600
H	1.38015600	-0.98017900	0.59556700
C	2.85386600	-0.91349200	-0.92541000
C	-0.99443000	-1.61867100	0.54254300
C	-1.87561400	0.09144100	2.00190700
N	-0.59885500	-2.11289400	-0.73366500
C	-0.93659800	-2.50762800	1.63739200
C	-1.81556100	-0.78916200	3.07171000
H	-2.25151800	1.09781500	2.15671300
C	-1.34901400	-2.09173000	2.90142100
H	-2.13549800	-0.46397900	4.05774900
H	-1.30780400	-2.76281100	3.75189000
C	0.06802100	1.63478500	-0.69737200
C	0.67745500	1.93232500	-1.91887600
C	0.73928000	1.95713800	0.48886800
C	1.92973400	2.54469800	-1.95716600
H	0.17942700	1.66251400	-2.84683200
C	1.98700400	2.57007900	0.45247400
H	0.28686100	1.71948800	1.44862200
C	2.58535300	2.86407900	-0.77215700
H	2.39690600	2.75850900	-2.91443800
H	2.50527500	2.80126000	1.37860000
H	3.57107800	3.31958100	-0.79740900
C	-2.70955100	2.10810400	-0.11748000
C	-4.06676900	1.76990100	-0.01391600
C	-2.32033300	3.41205100	0.20063400
C	-5.00349800	2.69909600	0.42516900
H	-4.39234400	0.76431500	-0.27340800
C	-3.26181900	4.34999700	0.62658900
H	-1.27774300	3.70411200	0.11667900
C	-4.60235700	3.99621100	0.74653100
H	-6.04887800	2.41457000	0.50750700
H	-2.94092600	5.35987900	0.86738800
H	-5.33329400	4.72675700	1.08159300
C	0.63645400	-1.87918600	-1.29409400
O	0.89299200	-2.24517700	-2.43725700
H	3.02880800	-1.14529200	-1.97520000
C	3.95490800	-0.26469600	-0.20189400
C	5.01853700	0.27438100	-0.93618600
C	3.97501600	-0.13148600	1.19341200
C	6.05418800	0.95660900	-0.30438600
H	5.01927900	0.17234600	-2.01878900
C	5.00965800	0.54551400	1.82782900
H	3.17471400	-0.56047100	1.78961300
C	6.05045600	1.09963300	1.08142400

H	6.86519300	1.37498500	-0.89405100
H	5.00784900	0.63921500	2.91045100
H	6.85825400	1.62864500	1.57929500
C	-1.51947800	-3.01558500	-1.43008000
C	-2.53881800	-2.30973800	-2.32047900
H	-0.90694800	-3.69137000	-2.03296700
H	-3.15947700	-1.64049600	-1.71231100
H	-2.00428400	-1.67857200	-3.03774300
O	-0.44975000	-3.74079900	1.36121800
C	-0.35495400	-4.68010700	2.41155600
H	0.05740900	-5.58409800	1.96300200
H	0.31915800	-4.32621700	3.20169700
H	-1.34108400	-4.90167000	2.83883400
H	-2.03214400	-3.61331900	-0.66810100
C	-3.42210500	-3.31990300	-3.05070800
H	-2.82201900	-3.97526300	-3.69306400
H	-3.97014300	-3.95531400	-2.34345600
H	-4.15845600	-2.81442400	-3.68418100

-110°

at wb97xd/6-31+G*
Energy = -1745.483569 A.U.
Thermal correction to Gibbs Free Energy = 0.472299 A.U.
Sum of electronic and thermal Free Energies = -1745.01127 A.U.

P	-1.07094600	-1.01613900	0.71409300
C	1.35225800	1.60176500	0.53300100
C	-1.38685900	0.12172400	-0.71838100
H	1.18951800	1.42343500	-0.52528500
C	2.54144400	1.38478900	1.10559000
C	-1.29404800	1.50798100	-0.52677600
C	-1.73750300	-0.36633400	-1.98664500
N	-1.02328600	2.09157500	0.74275200
C	-1.54156200	2.38626300	-1.60590000
C	-1.98677600	0.50353000	-3.03628900
H	-1.83206900	-1.43659300	-2.14265300
C	-1.88823400	1.88277300	-2.85707100
H	-2.26271700	0.11164000	-4.01122500
H	-2.08106600	2.54700300	-3.69186500
C	0.63241700	-1.62792800	0.36746000
C	1.36127500	-2.08993000	1.46909200
C	1.23057300	-1.66569200	-0.89596800
C	2.65015100	-2.59379700	1.31016500
H	0.91983000	-2.04218200	2.46188200
C	2.52203900	-2.16254600	-1.05475300
H	0.69180300	-1.29814900	-1.76492500
C	3.23388300	-2.63106500	0.04643300
H	3.20452700	-2.94164200	2.17742500
H	2.97913800	-2.17042500	-2.04015100
H	4.24788300	-2.99963300	-0.07754500
C	-2.10044600	-2.46023900	0.20395200
C	-3.49042800	-2.32762200	0.33092500
C	-1.58307800	-3.67867600	-0.24453400
C	-4.34147500	-3.37455600	-0.00605100
H	-3.91171500	-1.39189500	0.69288000
C	-2.43554100	-4.73469400	-0.56925400
H	-0.50946600	-3.81013600	-0.34403100
C	-3.81465000	-4.58510900	-0.45706900
H	-5.41636400	-3.24953000	0.09169900
H	-2.01558300	-5.67557600	-0.91460400
H	-4.47681100	-5.40744600	-0.71314900
C	0.20622500	2.06719300	1.35893400
O	0.34611400	2.44497800	2.51811300
H	2.60961800	1.54241500	2.18125800
C	3.75461100	0.90670600	0.43089300
C	4.79210600	0.37317500	1.20582900
C	3.91212600	0.93867900	-0.96153600
C	5.93811900	-0.14463500	0.61065600
H	4.68584100	0.34857000	2.28766600
C	5.05836100	0.42807500	-1.55861800
H	3.13351300	1.37075200	-1.58368400
C	6.07302500	-0.12317900	-0.77590400
H	6.72719900	-0.56283300	1.22948000
H	5.16368200	0.46257200	-2.63961000
H	6.96869800	-0.52154900	-1.24449400
C	-2.14826200	2.70181200	1.45415300
C	-2.99873500	1.70031700	2.23196700
H	-1.72745300	3.44366300	2.13727100
H	-3.41209400	0.95656400	1.53832500
H	-2.35588400	1.15905000	2.93352800
O	-1.40760100	3.70525900	-1.32795200
C	-1.64896200	4.64045900	-2.35814300
H	-1.48892200	5.62127000	-1.90992400
H	-0.94909600	4.50348600	-3.19208100
H	-2.68035000	4.57126400	-2.72647400
H	-2.76105000	3.23004100	0.71554900
C	-4.13491900	2.39944700	2.97619600
H	-3.74275900	3.12610900	3.69739000
H	-4.79378300	2.93774500	2.28323900
H	-4.74777400	1.67905600	3.52801900

-130°

at wb97xd/6-31+G*
Energy = -1745.481589 A.U.
Thermal correction to Gibbs Free Energy = 0.473197 A.U.
Sum of electronic and thermal Free Energies = -1745.008392 A.U.

P	-0.66254000	1.05278000	-0.62536000
C	1.09000700	-1.76417400	-0.66475100
C	-1.27703200	-0.03232700	0.75126300
H	1.01888800	-1.77728500	0.41893600
C	2.24993600	-1.53253700	-1.29002200
C	-1.50032700	-1.40194900	0.52864800
C	-1.58297000	0.51262900	2.00790400
N	-1.32680400	-2.03103400	-0.73649800
C	-2.04933200	-2.19338600	1.56614400
C	-2.11684100	-0.27277900	3.01537300
H	-1.42441800	1.57214200	2.18326700

C	-2.35470000	-1.62936300	2.80128600
H	-2.35903700	0.16739100	3.97844400
H	-2.77269000	-2.23216400	3.59936000
C	1.03992300	1.49711200	-0.07883500
C	1.83551000	2.14976300	-1.02958600
C	1.58965000	1.21952000	1.17474900
C	3.13799600	2.53410000	-0.72715200
H	1.43162800	2.35314600	-2.01916800
C	2.89994800	1.59070000	1.47323700
H	1.00252900	0.69808700	1.92508200
C	3.67600500	2.25259400	0.52722900
H	3.74035900	3.03713600	-1.47861000
H	3.31786500	1.34911500	2.44646000
H	4.70076300	2.52825600	0.75782100
C	-1.58044100	2.60827400	-0.24113800
C	-2.89389000	2.70350800	-0.71933700
C	-1.04284400	3.69169800	0.46129300
C	-3.66074100	3.83944400	-0.47766500
H	-3.31964200	1.88096800	-1.28964100
C	-1.80565300	4.83580400	0.69239800
H	-0.02190400	3.64618900	0.83047700
C	-3.11704900	4.91086800	0.22976900
H	-4.67944400	3.89294100	-0.85177900
H	-1.37157800	5.66931300	1.23811500
H	-3.71053700	5.80245500	0.41194400
C	-0.14868900	-1.99343400	-1.45512700
O	-0.12448800	-2.22174800	-2.65911300
H	2.22509400	-1.48725700	-2.37811000
C	3.54877800	-1.28987700	-0.65031100
C	4.56977500	-0.69060700	-1.39845000
C	3.81021700	-1.62329600	0.68616600
C	5.80533300	-0.40601300	-0.82571500
H	4.38208600	-0.43086500	-2.43741100
C	5.04563100	-1.34665700	1.25884500
H	3.04412800	-2.11361800	1.28013700
C	6.04645900	-0.73080900	0.50718400
H	6.58091700	0.06769800	-1.42111200
H	5.23162500	-1.61609300	2.29497700
H	7.01237800	-0.51641300	0.95616000
C	-2.54943100	-2.37774500	-1.47031100
C	-3.25996200	-1.16892400	-2.07562700
H	-2.25559000	-3.07297900	-2.25966500
H	-3.54744600	-0.47989300	-1.27023200
H	-2.56008600	-0.63550400	-2.72765200
O	-2.22468100	-3.50588000	1.27450000
C	-2.77390300	-4.35130500	2.26271800
H	-2.82757500	-5.34072200	1.80786200
H	-2.13214800	-4.39487000	3.15176300
H	-3.78202700	-4.02728100	2.55099100
H	-3.21350200	-2.90721200	-0.78104600
C	-4.50198700	-1.58922700	-2.85939600
H	-4.23534300	-2.25575700	-3.68780500
H	-5.21679600	-2.12022000	-2.21837900
H	-5.01566500	-0.71976800	-3.28309800

-150°

at wb97xd/6-31+G*
Energy = -1745.475556 A.U.
Thermal correction to Gibbs Free Energy = 0.474585 A.U.
Sum of electronic and thermal Free Energies = -1745.000971 A.U.

P	-0.44052900	1.01397900	-0.57879000
C	0.99733100	-1.79577500	-0.69256200
C	-1.21178800	-0.00645600	0.77007100
H	0.95043300	-1.90218400	0.38761300
C	2.15292700	-1.57182800	-1.32891100
C	-1.59538200	-1.34475000	0.55212200
C	-1.53911800	0.61175300	1.98778000
N	-1.42075800	-2.04057300	-0.68140000
C	-2.39477400	-1.98797500	1.53295300
C	-2.29313400	-0.04064400	2.94549800
H	-1.23464900	1.63904700	2.16080100
C	-2.73594200	-1.34125700	2.71573700
H	-2.55553700	0.46196600	3.87180500
H	-3.33643500	-1.84207600	3.46614200
C	1.23818900	1.39199500	0.07449200
C	2.09269600	2.09101100	-0.78856200
C	1.72721500	0.99318400	1.31992500
C	3.39443000	2.39934200	-0.40811700
H	1.73553600	2.39194400	-1.77140800
C	3.03761000	1.28715800	1.69520900
H	1.09253800	0.43431100	2.00153600
C	3.87329300	1.99346200	0.83646900
H	4.04282500	2.93983900	-1.09223000
H	3.40778700	0.95056300	2.65954900
H	4.89731800	2.21012900	1.12599900
C	-1.30870500	2.62011200	-0.30203600
C	-2.56519500	2.77032900	-0.90279300
C	-0.78458400	3.68906900	0.43241700
C	-3.29307800	3.94681800	-0.74947700
H	-2.97637400	1.95893800	-1.49889900
C	-1.50675600	4.87281300	0.57546300
H	0.19419200	3.60042600	0.89627800
C	-2.76429500	5.00310200	-0.00917100
H	-4.26858800	4.04323800	-1.21817300
H	-1.08404400	5.69408200	1.14800700
H	-3.32643800	5.92584800	0.10427400
C	-0.27376600	-1.90681100	-1.45754800
O	-0.29973700	-2.02437700	-2.67478800
H	2.10380900	-1.42976400	-2.40787500
C	3.47857200	-1.45268100	-0.70981000
C	4.51224500	-0.84928100	-1.43706600
C	3.75508400	-1.90980000	0.58661600
C	5.77683100	-0.68253600	-0.88153000
H	4.31257400	-0.49326300	-2.44480200
C	5.01905200	-1.75088400	1.14127500
H	2.97807200	-2.40519400	1.16186200
C	6.03381900	-1.13123800	0.41174100
H	6.56232100	-0.20373900	-1.45957800
H	5.21621900	-2.11559400	2.14566300
H	7.02196100	-1.00907100	0.84667700

C	-2.65694300	-2.28168600	-1.45171600
C	-3.30217300	-1.00369000	-1.98463000
H	-2.38155200	-2.93377200	-2.28248600
H	-3.57264300	-0.35517600	-1.14057000
H	-2.56967200	-0.46291000	-2.59401600
O	-2.73942700	-3.27409600	1.26710700
C	-3.51934700	-3.97852700	2.20889300
H	-3.67640800	-4.96849400	1.77966600
H	-2.99424800	-4.07805500	3.16733100
H	-4.49041700	-3.49281200	2.36882300
H	-3.35256900	-2.82433100	-0.80993200
C	-4.54783600	-1.31497600	-2.81227600
H	-4.29635900	-1.93541300	-3.68019600
H	-5.29558800	-1.85542300	-2.21853400
H	-5.01639600	-0.39705000	-3.18296400

-170°

at wb97xd/6-31+G*
Energy = -1745.462483 A.U.
Thermal correction to Gibbs Free Energy = 0.477421 A.U.
Sum of electronic and thermal Free Energies = -1744.985062 A.U.

P	-0.28281600	0.95038900	-0.55924700
C	0.97766400	-1.79980300	-0.58492000
C	-1.21529000	0.06406100	0.78321200
H	0.93872500	-1.85219800	0.49968000
C	2.13361700	-1.67648400	-1.24823900
C	-1.68811600	-1.26095700	0.63734200
C	-1.63360300	0.83566700	1.88173800
N	-1.43369500	-2.09314300	-0.50178500
C	-2.72333000	-1.68116500	1.52166700
C	-2.60050400	0.38466700	2.75691100
H	-1.24075000	1.84015400	2.00180900
C	-3.16527000	-0.87182900	2.56122300
H	-2.93673300	1.00987600	3.57856000
H	-3.93892500	-1.21967800	3.23514400
C	1.36264000	1.29009800	0.18978800
C	2.33737200	1.81522500	-0.66752900
C	1.71450400	1.01390400	1.51333200
C	3.62640900	2.07134600	-0.21077500
H	2.08757900	2.01238200	-1.70807300
C	3.00833900	1.26151900	1.96910500
H	0.98262400	0.58990200	2.19483300
C	3.96709600	1.79155600	1.11081300
H	4.37212500	2.46844700	-0.89361600
H	3.26895500	1.02718400	2.99774500
H	4.97896100	1.96851900	1.46334700
C	-1.09159400	2.60656100	-0.46674200
C	-2.35203000	2.72462600	-1.06762400
C	-0.51695000	3.73987600	0.11630900
C	-3.03323700	3.93747700	-1.06176000
H	-2.80494300	1.85640500	-1.54156200
C	-1.19312000	4.95976100	0.10978600
H	0.46226200	3.67426900	0.58262300
C	-2.45349900	5.06115900	-0.47308700
H	-4.01305900	4.00885800	-1.52596400
H	-0.73199800	5.83140500	0.56647100
H	-2.97992500	6.01154200	-0.47504700
C	-0.31073100	-1.89589600	-1.32475200
O	-0.36278800	-2.00868200	-2.53866000
H	2.07103600	-1.59929700	-2.33316400
C	3.47689200	-1.58772500	-0.66445600
C	4.53965200	-1.17820900	-1.48122000
C	3.74644800	-1.88178200	0.67941100
C	5.82668300	-1.04411200	-0.97102100
H	4.34629200	-0.95161400	-2.52710800
C	5.03246400	-1.75541300	1.18992800
H	2.94605200	-2.21840500	1.33159100
C	6.07693400	-1.33080500	0.36915900
H	6.63486600	-0.71910800	-1.62030900
H	5.22196300	-1.98908700	2.23398800
H	7.08166000	-1.23296500	0.77095300
C	-2.64268300	-2.39536600	-1.31700400
C	-3.31771100	-1.15123600	-1.89381700
H	-2.30847900	-3.04110900	-2.12929500
H	-3.65957900	-0.50392700	-1.07513000
H	-2.58264700	-0.58458000	-2.47644200
O	-3.16241500	-2.95995800	1.37053600
C	-4.16360100	-3.45122100	2.23417300
H	-4.36283100	-4.47088700	1.90265100
H	-3.81859700	-3.47029100	3.27578800
H	-5.08465800	-2.85904900	2.16071000
H	-3.33894600	-2.95939100	-0.70157800
C	-4.50497700	-1.52891500	-2.77780500
H	-4.18184300	-2.14383600	-3.62563600
H	-5.25410100	-2.09997700	-2.21517100
H	-4.99817600	-0.63777100	-3.18047800

-180° and **180°**

at wb97xd/6-31+G*
Energy = -1745.445403A.U.
Thermal correction to Gibbs Free Energy = 0.472688 A.U.
Sum of electronic and thermal Free Energies = -1744.972715 A.U.

P	-0.23585800	1.13731800	-0.48020100
C	-1.50842900	-1.61730900	-0.11821900
C	0.49631800	0.53226200	1.13221000
H	-1.56156000	-1.42118500	0.94919000
C	-2.60480100	-1.80571000	-0.86015700
C	1.04611000	-0.77223200	1.24156300
C	0.75837500	1.48089600	2.13154000
N	0.88661000	-1.79792500	0.24355200
C	2.12070400	-0.92321900	2.15876700
C	1.71202900	1.25147400	3.10824400
H	0.28160800	2.45324700	2.08392800
C	2.43295100	0.06507800	3.08907800
H	1.93157100	2.01118100	3.85252500
H	3.21830300	-0.09962500	3.81702000

C	1.34680000	1.18821800	-1.42884700
C	1.27477500	0.89751800	-2.79438400
C	2.60120300	1.43582100	-0.85578500
C	2.42746500	0.86983900	-3.57730900
H	0.31322300	0.65877400	-3.24067600
C	3.75354300	1.40402600	-1.63544600
H	2.68076900	1.64533300	0.20807900
C	3.66862700	1.12140000	-2.99869200
H	2.35531400	0.63315100	-4.63499800
H	4.72032900	1.59498700	-1.17725400
H	4.56973600	1.08904300	-3.60505200
C	-0.58207700	2.90439400	-0.10977800
C	-1.84926900	3.19403600	0.41388100
C	0.30053400	3.96013800	-0.35839700
C	-2.21359700	4.50342500	0.71525400
H	-2.55801000	2.38586300	0.58412600
C	-0.07030300	5.27327000	-0.07396200
H	1.28212400	3.75992700	-0.77763900
C	-1.32303900	5.54764100	0.47030100
H	-3.19735900	4.70949000	1.12765700
H	0.62463900	6.08359900	-0.27681000
H	-1.60840000	6.57178100	0.69394100
C	-0.14966900	-1.74187300	-0.71459200
O	0.02619500	-1.99549800	-1.89408900
H	-2.45999200	-2.03621600	-1.91468100
C	-3.99417000	-1.72146100	-0.39136700
C	-5.01883600	-2.22107200	-1.20640800
C	-4.34531800	-1.14204700	0.83757600
C	-6.34967500	-2.16492000	-0.80223600
H	-4.76398000	-2.66335300	-2.16659600
C	-5.67380200	-1.08351200	1.24208500
H	-3.57525700	-0.72106000	1.47809900
C	-6.68190700	-1.59725000	0.42554100
H	-7.12744900	-2.56269100	-1.44799900
H	-5.92599800	-0.62922800	2.19632300
H	-7.71961500	-1.54851200	0.74300000
C	2.16124600	-2.20267500	-0.43326400
C	2.32865800	-3.71494200	-0.46867700
H	2.13637800	-1.80500900	-1.45087000
H	2.29222600	-4.10085400	0.55582700
H	1.48787600	-4.15654500	-1.01581600
O	2.70130600	-2.15305000	2.20058200
C	3.78320300	-2.38045800	3.07508400
H	4.11759100	-3.39805400	2.86871100
H	4.60816100	-1.68192600	2.88468500
H	3.47279900	-2.30787100	4.12521900
H	3.00333700	-1.73854800	0.07185400
C	3.64487000	-4.10636800	-1.13861500
H	3.69077800	-3.73079100	-2.16769700
H	4.50337100	-3.69264300	-0.59417200
H	3.76397000	-5.19450400	-1.17365200
